# Supramolecular Materials and Strategies for Bioorthogonal Chemical Transformations

**DOI:** 10.1021/acs.chemrev.5c00047

**Published:** 2025-08-01

**Authors:** Annechien A. H. Laporte, Joost N. H. Reek

**Affiliations:** † Homogeneous, Supramolecular, and Bio-Inspired Catalysis, van ’t Hoff Institute for Molecular Sciences (HIMS), 1234 Universiteit van Amsterdam (UvA), Science Park 904, 1098 XH Amsterdam, The Netherlands

## Abstract

Bioorthogonal reactions play a key role in controlled chemical transformations in living systems and are therefore applied to a diverse area of biological and medical applications. However, these applications can be limited by poor selectivity, slow kinetics under biological conditions, and intrinsic incompatibility between the introduced materials and the cellular environment. An emerging strategy for greater functional control over bioorthogonal transformations is the employment of supramolecular strategies or constructs. Herein, we focus on synthetic supramolecular systems that (i) improve biocompatibility by shielding reactive species within protective supramolecular constructs from harsh biological environments; (ii) allow for integration of subcellular targeting moieties; (iii) reduce toxicity; (iv) accelerate reaction rates through molecular preorganization; (v) explore entirely new tools, such as catalysis regulated by controlled stimuli at a functionalized surface. Through rational integration of these supramolecular strategies, bioorthogonal reactions could achieve enhanced precision, faster kinetics, and targeted reactivity within specific tissues, cells, or organelles, subsequently paving the way for further applications in chemical biology and therapeutic interventions.

## Graphical abstract

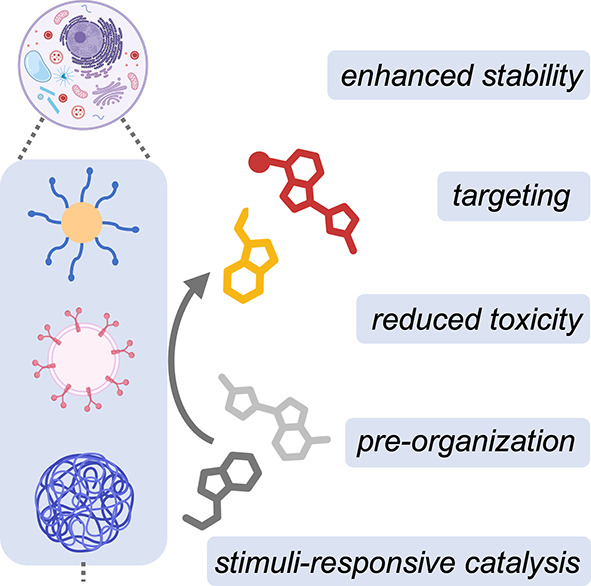

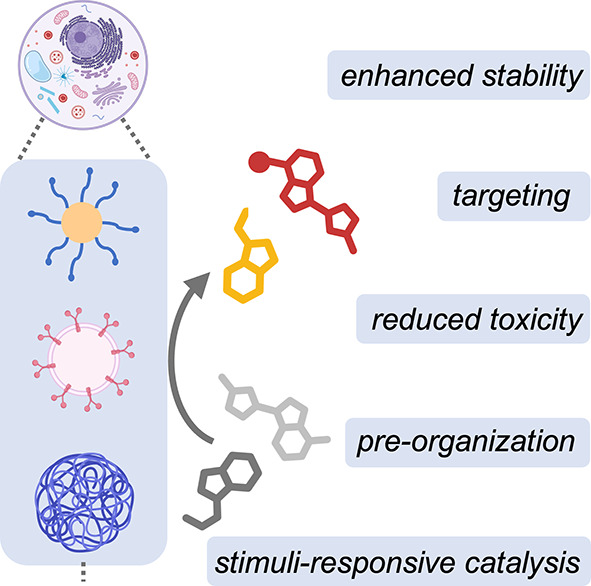

## 1 Introduction

The ability to control chemical transformations within living organisms with atomic precision and high selectivity is challenging, yet it provides the basis for the development of novel therapeutic strategies. These chemical transformations often face limitations that are related to substrate specificity and product selectivity. Moreover, the introduced synthetic reaction components can interfere with the cellular environment and may suffer from slow reaction kinetics under biological conditions. Supramolecular strategies and materials are an emerging tool in the literature to overcome these intrinsic limitations. In this review, we will provide an overview of recent literature that applied supramolecular materials and strategies to enhance bioorthogonal reactions.

Supramolecular chemistry, or chemistry beyond the molecule, was coined by Lehn^1^ for the field that evolved after the initial discovery that molecules can be organized in well-defined complexes by noncovalent interactions.^2^ The landmark examples by Lehn,^3,4^ Pedersen,^5^ and Cram^6,7^ involved the binding of cations in well-defined host molecules that provided sufficient interaction to form a host–guest complex between the host and guest. The intensive research on host–guest supramolecular systems provided detailed insight into how molecules interact, and the underlying principles are now well understood.^8–11^ Frequently used interactions include hydrogen bonding and π–π stacking, but nowadays the palette of interactions is much wider.^12,13^ One of the key concepts in supramolecular chemistry is self-correction, which implies that the thermodynamic product is obtained —though kinetic intermediates might form during the assembly process—as a result of reversible noncovalent bonds.^1,14^ The reversible character of coordination bonds is complemented with directionality, making them particularly suitable for the formation of large supramolecular structures that can be applied in a biological context. As such, coordination-driven self-assembly has developed as a powerful tool. Whereas the early examples of supramolecular structures were of relatively small size, currently well-defined structures of 10–20 kDa—comparable to the size of small proteins—can now be constructed through different approaches.^15–18^

The field of supramolecular chemistry rapidly diversified and extended to many other fields including polymer chemistry,^19–31^ catalysis^11,32–39^ and health sciences.^40–45^ Because supramolecular interactions play a key role in nature—with deoxyribonucleic acid (DNA) base pairing as the archetypical example—biology has served as a source of inspiration to the field.^46^ For example, the operational modes of enzymes served as a blueprint for the development of a variety of supramolecular catalyst systems, utilizing natural features like substrate preorganization, cavity effects, tuning of the acid dissociation constant (p*K*_a_), and substrate-selective catalysis based on functionality or based on size.^35,47,48^ Whereas supramolecular catalyst systems have mostly been studied in the context of understanding mechanistic aspects of enzymes and to achieve unrivaled catalyst performance for synthetic conversions, they now provide the basis for novel supramolecular strategies for chemical transformations in living cells. The well-organized chromophores in natural light-harvesting systems have served as blueprints for the development of assembly strategies to organize light-absorbing molecules aiming at efficient conversion of light to chemical energy.^35,49^ The well-understood aggregation behavior of chromophores now also forms a fundament for aggregation-induced changes in reactivity as a supramolecular tool for *in vivo* chemical conversions. The essential compartmentalization of cells and organelles has inspired the utilization of lipid bilayers to synthesize a diversity of amphiphilic molecules that organize in a plethora of larger aggregates, such as micelles and vesicles and liposomes.^50^ Similar strategies have been applied for amphiphilic polymers.^51–53^ The use of aggregated amphiphilic molecules and functionalized polymers play a key role in the context of this review. Although most of the early developments in supramolecular chemistry occurred in organic solvents, contemporary supramolecular chemistry can occur in aqueous environments.^10,48,54–57^ These fundamental studies performed in aqueous media are essential for the (future) design of supramolecular systems for application in biological settings. Since natural systems are, by definition, out of equilibrium, current studies are directed at understanding complex artificial chemical systems, referred to as systems chemistry^58^ and out-of-equilibrium systems.^59,60^ We anticipate that also this fundamental knowledge will benefit the development of supramolecular systems for biological applications in the future.

In parallel to the development of supramolecular chemistry, scientists have integrated synthetic chemical reactions with living systems.^47^ Controlled chemical reactions in living systems allow for the manipulation of complex biological processes of choice without interfering with native bioprocesses, which is at the heart of the development of novel therapies.^61,62^ Over the last decades, these noninterfering reactions have become known as bioorthogonal reactions.^63,64^ The field of bioorthogonal chemistry has gained significant interest due to the work of Bertozzi,^65^ Meldal,^66^ and Sharpless,^67^ who were awarded the Nobel Prize in Chemistry in 2022. Whereas this field has also substantially developed, it is still facing many challenges.^68^ Considering that supramolecular chemistry has largely been inspired by biological systems, it is a logical assumption that supramolecular concepts could be useful to tackle challenges in bioorthogonal chemistry. Although the use of supramolecular chemistry to facilitate the application of bioorthogonal reactions is still in its infancy, several interesting proof-of-concepts have been reported.

Traditionally, bioorthogonal reactions have been grouped into ligation (bond-forming) and cleavage reactions (Figure 1).^69^ Most of the noninterfering functional groups on molecules are normally not present in biological systems, and thus labeled as new-to-nature.^70^ Extensive research has been performed to expand the toolbox of bioorthogonal reactions, as these reactions have demonstrated great potential for biomedical applications, such as prodrug activation,^71^ protein activation,^72–75^ and cell visualization.^76–78^ For example, several bioorthogonal reactions have demonstrated the ability to produce therapeutically active molecules in target tissues or cells, which improved local target concentrations and thereby reduced off-target effects.^62^ Transition metal complexes (TMCs) have been employed as catalysts to expand the chemical toolbox and to enhance reaction rates.^79–84^

**Figure 1:**
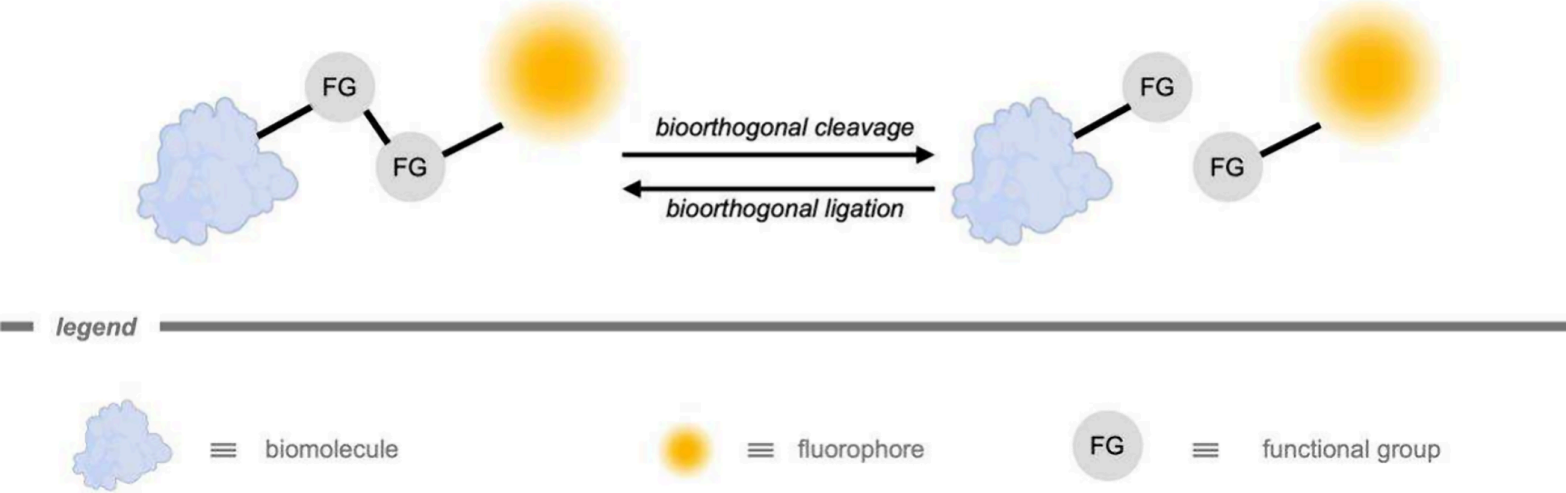
General principle of a bioorthogonal ligation reaction between two bioorthogonal functional groups (FG), and its counterpart bioorthogonal cleavage using a bioorthogonal reagent. The functional groups can be linked to biomolecules or fluorescent probes.^69^ The functional groups can be attached to a wide variety of moieties (e.g., drugs, polymers, and vesicles).

Although the potential of bioorthogonal reactions has already been demonstrated, we are still in the early stages of exploring the full range of possibilities this field offers. Intracellular biomolecules such as glutathione (GSH) tend to interfere with bioorthogonal reactions. In case of TMC-mediated reactions, anions such as chloride can compete for coordination and inhibit or alter activity,^85,86^ and catalyst poisoning from cell components is a widespread challenge in the field.^87,88^ Another key challenge is the selectivity of chemical reactions in living organisms.^89^ The development of selective and stable catalysts under physiological conditions is essential to unlock the full potential of bioorthogonal chemistry in living systems.^61,71^ Moreover, the development of catalysts for more complex reactions *in vivo* is particularly interesting for the synthesis of relevant molecules in living cells.^71,90,91^

The integration of supramolecular structures offers several opportunities to fully harness the potential of bioorthogonal reactions.^92^ First, supramolecular structures often make use of a second (or even third) coordination sphere around a catalytic center, which in turn can improve key properties such as activity and selectivity, while protecting the catalyst from deactivation by the biological environment (Figure 2i).^93^ Second, supramolecular hosts can provide transport of reaction components across membranes or provide targeting abilities in complex biological systems, allowing for activity in only specific cell or tissue types (Figure 2ii).^94^ Third, alternative catalytic pathways provided by supramolecular structures can allow for the utilization of reaction components with reduced toxicity (Figure 2iii). Fourth, supramolecular structures can preorganize substrates using noncovalent interactions, improving both reaction kinetics and product selectivity (Figure 2iv).^95^ Finally, the dynamic nature of the noncovalent bonds allows for selective release of reaction components, as well as the “gatekeeping” of catalytic surfaces (Figure 2v).^96^ Similar design principles have been applied to artificial metalloenzymes,^97–102^ and supramolecular photosensitizers (PSs),^103,104^ which are reviewed elsewhere. Supramolecular biomolecule–biomolecule interactions have also been employed to preorganize substrates for (bioorthogonal) ligations, and are reviewed elsewhere.^105–108^

**Figure 2:**
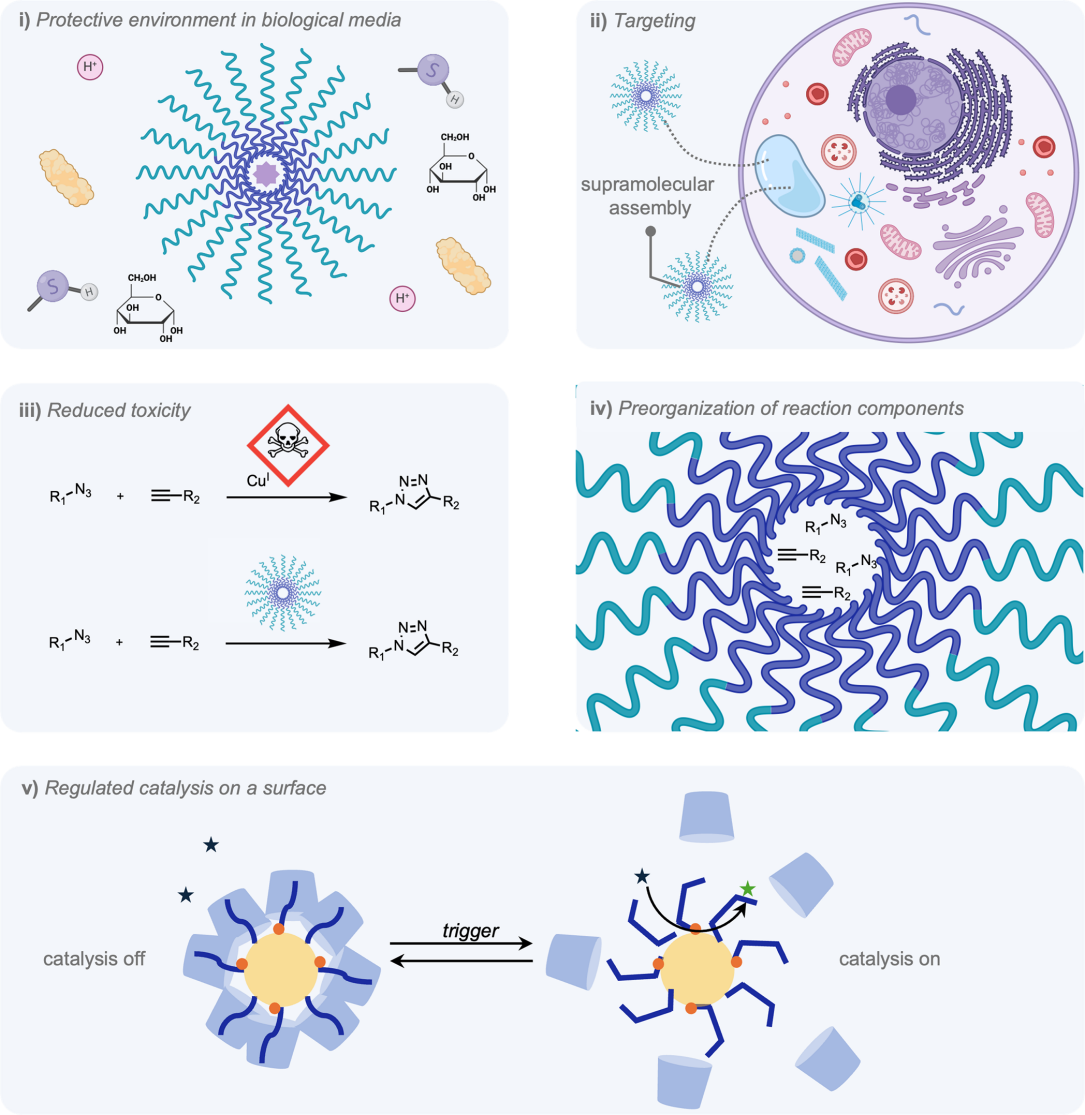
Functions of supramolecular structures in bioorthogonal reactions that will be discussed throughout this review. i) Supramolecular assemblies that provide a protective environment around reaction components, improving their stability in biological systems. ii) Supramolecular structures for targeting of specific cells or organelles, as well as selective release. iii) Supramolecular assemblies for the mitigation of toxic reaction components in biological media. iv) Supramolecular assemblies for the preorganization of reaction components. v) Supramolecular structures for regulated catalysis on a surface (“gatekeeping”).

Both the field of supramolecular chemistry as well as the application of bioorthogonal reactions have been thoroughly reviewed over the past decade. In this review, we will provide an overview of recent examples of supramolecular strategies that have been employed to optimize bioorthogonal reactions. The sections are structured according to the function of the supramolecular structure: (i) providing a protecting environment around reaction components, (ii) introducing selective release or targeting, (iii) reducing toxicity of reaction components, (iv) enhancing reaction rates through preorganization, and (v) regulating catalysis by gatekeeping sites on a surface. A wide variety in nomenclature has been reported to describe the activity of supramolecular systems in biorelevant contexts (e.g., nanoreactors, nanozymes, nanocatalysts, nanocarriers, and nanomedicines), yet these names often provide limited information in terms of structure and composition. For clarity and overview, we have avoided the use of these terms in this review and referred to the structural (molecular) systems instead.

## 2 General Principles

### 2.1 Key Bioorthogonal Reactions

Key examples of bioorthogonal reactions are the Staudinger ligation reaction (Figure 3A), Cu^I^-catalyzed alkyne–azide cycloaddition (Figure 3B), strain-promoted [3+2] cycloaddition (Figure 3C), inverse-electron demand Diels–Alder reaction (Figure 3D), bioorthogonal cleavage reactions (Figure 3E) (e.g., allyl carbamate cleavage, and depropargylation), as well as various metal-catalyzed and photoinduced reactions.^109^ The reaction rate of the bioorthogonal reaction is of great importance because reaction components are typically present at low concentrations (100 μM–10 nM).^61^ Low concentrations of reaction components are often required because of solubility reasons, or inherent toxicity of the components. Moreover, transport mechanisms inside living organisms as well as the metabolism of the biological system can lower the concentrations of the reaction components at the target location. To boost reaction rates, catalysts can be employed, which can be either natural (e.g., enzymes), or non-natural (“new-to-nature catalysts”).^96^ Although catalysts can have a major impact on the reaction mechanism as well as yields and selectivity, they add a layer of complexity to the system. Therefore, the development of both catalytic and stoichiometric reactions are highly relevant and will be discussed throughout this review.

**Figure 3:**
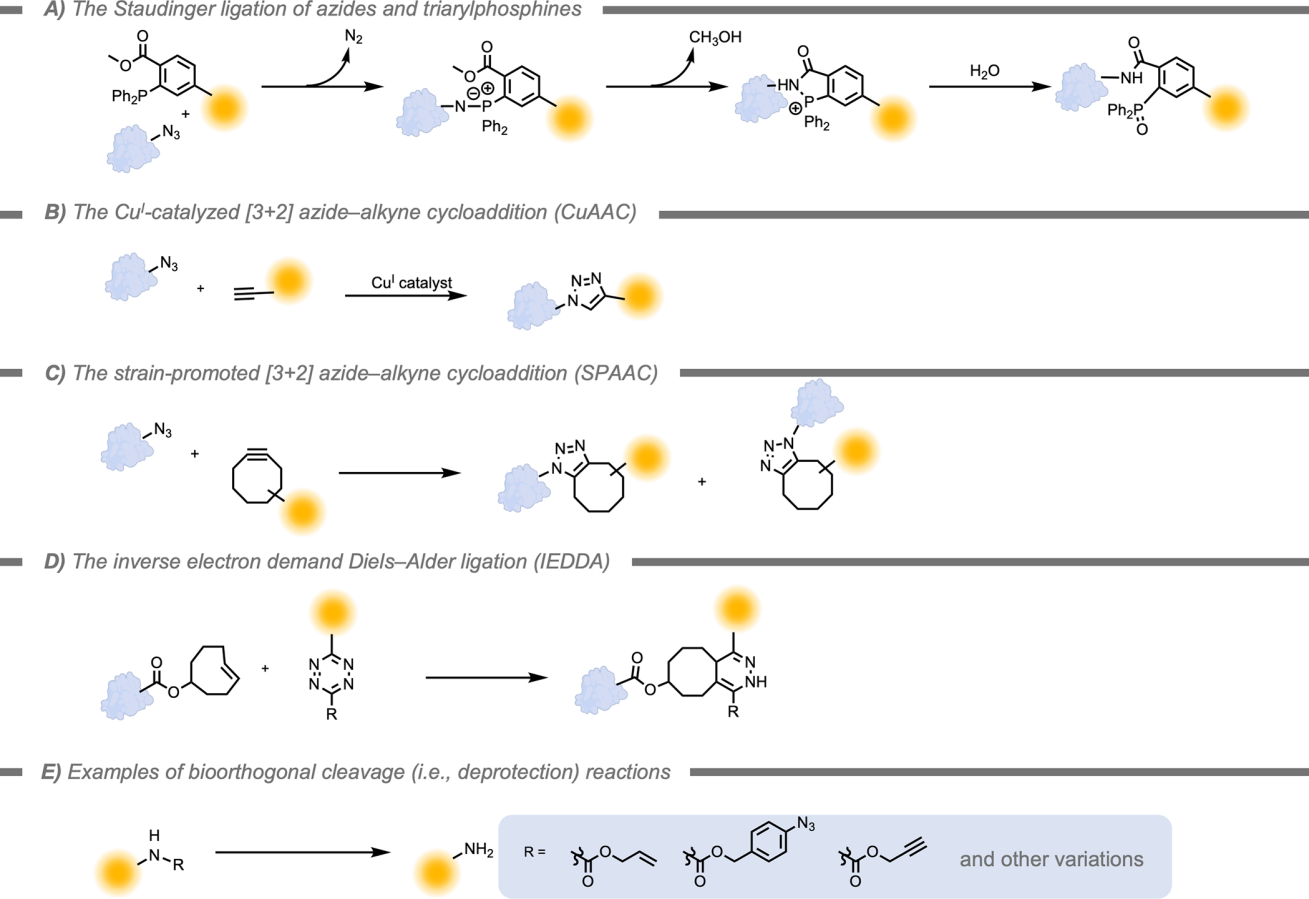
A) The Staudinger ligation reaction of azides with triarylphosphines. B) The copper-catalyzed [3+2] azide–alkyne cycloaddition (i.e., CuAAC, or the “click” reaction). C) The strain-promoted [3+2] azide–alkyne cycloaddition (i.e., SPAAC). D) The inverse electron demand Diels–Alder ligation (i.e., IEDDA). E) A general example of a bioorthogonal cleavage (i.e., deprotection) reaction to restore the activity of a probe molecule containing a primary amine.^110^

Ideally, bioorthogonal reactions should satisfy the following principles: they must be (i) highly selective toward specific functional groups, (ii) be performed in an aqueous environment at (iii) physiological pH, have (iv) fast reaction rates at room temperature while occurring at (v) low reactant concentrations (vi) in the presence of biomolecules.^111^ Bioorthogonal substrates and catalysts that satisfy all principles have arguably not been identified yet. However, optimization of reaction types in biological settings according to these bioorthogonality principles can allow for highly controlled and precise chemistry in biological applications.

In 2000, Bertozzi and co-workers^112^ demonstrated the functionality of azides as a chemical reporter group with the implementation of the Staudinger ligation, a reaction that was already developed in 1919 (Figure 3A).^113^ Azides are not present in natural systems and therefore a highly relevant bioorthogonal functional group. Moreover, azides are soft electrophiles and therefore often orthogonal in reactivity to most functional groups found in living systems.^114^ Because of the small dimensions of the azide group, an azide functionalization only minimally changes a modified molecule in terms of sterics.^64^ However, the Staudinger ligation reaction is not without its own challenges. It suffers from relatively slow kinetics, requiring high concentrations of a triarylphosphine.^64^ As the speed of bioorthogonal reactions is an essential parameter and the nucleophilic attack of the phosphine to the azide is the rate-limiting step, attempts were made to increase the electron density on the phosphine substrate.^115^ This inevitably resulted in undesirable oxygen-sensitivity of the phosphine component. Therefore, other bioorthogonal reactions of azide substrates were developed.^64^

Later, the Cu^I^-catalyzed [3+2] azide–alkyne cycloaddition (CuAAC) was discovered independently by the Meldal^66^ and the Sharpless groups (Figure 3B).^67^ This reaction makes use of the formation of a Cu acetylide to activate terminal alkynes for the reaction with azides. It proceeds at much (10^7^ times)^116^ faster kinetics than the uncatalyzed Huisgen 1,3-dipolar cycloaddition reaction,^117^ and the rate was further improved by ligands coordinating to the Cu center. The CuAAC reaction has become known as a reference “click reaction” due to its simplicity, as well as efficiency and selectivity.^118^ Although initially the CuAAC seemed suitable for protein labeling in living organisms, the toxicity of Cu for living cells has limited its widespread use.^111^

The limitations of Cu catalysts pushed the development of Cu-free strategies for bioorthogonal click chemistry. For example, Bertozzi and co-workers have developed a strain-promoted azide–alkyne cycloaddition (SPAAC) between biomolecules functionalized with azides and cyclooctynes on synthetic substrates, which proceeds under physiological conditions (Figure 3C).^119^ Although the SPAAC reaction overcomes the inherent toxicity associated with Cu chemistry, it has its own limitations in terms of substrate flexibility as well as relatively slow kinetics compared to the CuAAC reaction.^120^ For an overview of the kinetics of various bioorthogonal reactions, the reader is referred to several reviews.^120,121^

The inverse-electron demand Diels–Alder reaction (IEDDA) consists of the [4+2] cycloaddition of 1,2,4,5-tetrazines and various dienophiles (Figure 3D).^111^ This reaction satisfies several bioorthogonality principles: it is highly selective and can be faster than the CuAAC reaction, without requiring a catalyst. For an in-depth review of the IEDDA reaction and its uses in chemical biology, the reader is referred to Oliveira et al.^111^ as well as Png et al.^122^

Additionally, various bioorthogonal cleavage reactions have been developed (Figure 3E). Most examples focus on the deprotection of functional groups on primary amines, although the restoration of biologically active hydroxy groups has also been reported.^123^ A wide variety of protecting groups have been established, such as allyl, propargyl, and azide groups, sometimes connected through ether or carbamate linkers.^110^ Removal of protecting groups to restore the activity of probes or drugs has been demonstrated by different strategies, including transition metal catalysis,^70,124,125^ light-driven decomposition,^126–129^ enzymatic deprotection,^130^ the use of reactive oxygen species (ROS),^131,132^ and via radiotherapy.^133,134^ For a comprehensive overview of bioorthogonal cleavage reactions, the reader is referred to several reviews.^69,123,135,136^ In this review, the focus is on the application of supramolecular strategies on bioorthogonal chemical conversions, which in most examples to date involve metal-mediated conversions (Figure 3B and 3E) or the IEDDA reaction (Figure 3D). Additionally, we will discuss miscellaneous transformations that have been explored in biorelevant context (e.g., Heck coupling, Suzuki–Miyaura coupling, and hydroarylation).

### 2.2 When Is a Reaction Bioorthogonal?

The term “bioorthogonality” has been broadly used and requires some definition. Different aspects of bioorthogonality can be defined for a given chemical transformation. First, the chemical functional groups that are to be converted (e.g., azide or alkyne) should not react or interfere with biomolecules and the biological system in general. The (catalytic) chemical conversion should be selective to the target chemical functional groups (e.g., the SPAAC reaction should selectively lead to a triazole) and leave the naturally occurring functional groups untouched. Whereas the designed chemical functional groups and the target chemical conversion can be bioorthogonal, the generated molecular compound may exert a certain biological function. Moreover, the installed bioorthogonal functional groups can in principle be attached to nonbioorthogonal species (e.g., sugars, DNA, and aptamers). Importantly, the conditions under which the reaction proceeds (e.g., solvent, pH, temperature, and catalyst) should be biocompatible, and should be evaluated on their own in terms of bioorthogonality.^137^

To consider the reaction conditions bioorthogonal, the reaction should be evaluated at least under biologically relevant or biomimetic conditions (Figure 4). First, a reaction should proceed in an aqueous environment at a pH around 7, or at the specified pH at the target location. For example, the pH can be significantly lower in endosomes and some tumor microenvironments.^138^ The reaction must have adequate reaction rates at 37 °C (when related to human applications) under aerobic conditions. Moreover, high concentrations of salts, small untargeted molecules (e.g., sugars, fatty acids, amino acids, and nucleotides), and macromolecules (e.g., DNA, ribonucleic acid (RNA), phospholipids, and proteins) are present in biological systems. A reaction should be evaluated in the presence of all these biomolecules to establish whether a reaction is truly orthogonal to functional groups and molecules present in living organisms.

**Figure 4:**
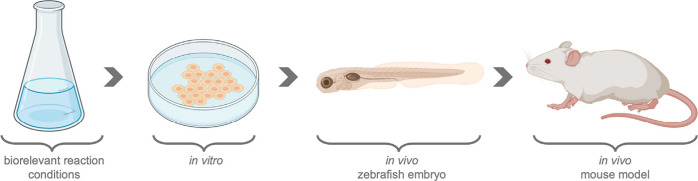
Model systems to evaluate bioorthogonal reactions with increasing complexity.

More informative are *in vitro* studies that investigate effects in living cells to determine whether a reaction occurs.^139^ The diffusion through cell membranes or cell walls must occur under cell conditions. Typical *in vitro* studies are cell viability,^140^ cell death,^141^ Ames,^142^ and human ether-a-go-go related gene (hERG) ion channel inhibition assays.^143,144^ One step further are *in vivo* assays, where reactions or substrates are evaluated in living organisms, such as zebrafish embryos,^145^ or mice. Typical assays are the maximum tolerated dose studies,^146^ monitoring the effect on blood gas, blood pH, and the heart rate, as well as the determination of the absorption, distribution, metabolism, and excretion (ADME) properties.^143^ Currently, most studies are limited in their evaluation of the bioorthogonality of the reaction conditions, partly because it is time-consuming and requires diverse expertise.

## 3 Supramolecular Structures for Improving the Stability of Non-natural Catalysts and Increasing Reaction Efficiencies in Biological Media

To expand the scope of bioorthogonal reactions, a wide variety of TMCs have been explored over the past decade.^124,147–151^ The use of TMCs for bioorthogonal processes is of significant interest as they allow for high reactivity, selectivity, and the development of new-to-nature chemical conversions. However, their use in biological media has proven to be challenging, as they are generally poorly soluble in aqueous media and have low stability and reactivity in biological media.^152^ These challenges have so far limited the widespread application of TMCs in biological systems. To solve these challenges, TMCs have been incorporated into protective scaffolds, which can allow for enhanced catalytic activity in biological media by providing isolation from the outside environment.^153^ Additionally, supramolecular systems containing TMCs have been designed to modify properties such as stability, solubility, and toxicity. For example, Hor and co-workers^154^ developed a water-soluble supramolecular catalyst for aqueous Suzuki–Miyaura cross-coupling reactions after modification of a water-insoluble Pd TMC.

### 3.1 Polymer Scaffolds to Protect Transition Metal Catalysts

In 2015, Tekinay, Guler, and co-workers^155^ reported a supramolecular polymer-based catalyst for bioorthogonal click reactions with lower cytotoxicity and improved catalytic activity than the molecular catalysts (Figure 5). Their catalytic system consisted of self-assembling peptide amphiphile (PA) molecules for the construction of a one-dimensional nanofiber (PA-Cu^II^ nanofibers). These self-assembling peptide amphiphiles are a class of supramolecular polymers developed by Stupp and co-workers and have been used in tissue engineering.^156,157^ The authors synthesized a peptide amphiphile molecule with binding affinity for copper ions by coordination via the imidazole groups on histidine residues. Assembly of the catalytic sites in a PA-nanofiber structure increased the catalytic conversion of the histidine-Cu^II^ complex from 61% to 95% under the same reaction conditions. The authors attributed the improved catalytic conversion to a positive cooperativity effect between the active sites in the nanostructured environment, as well as formation of a favorable hydrophobic environment for the reactants due to assembly of hydrophobic residues (e.g., glycine, alanine, valine) in the nanofiber.

**Figure 5:**
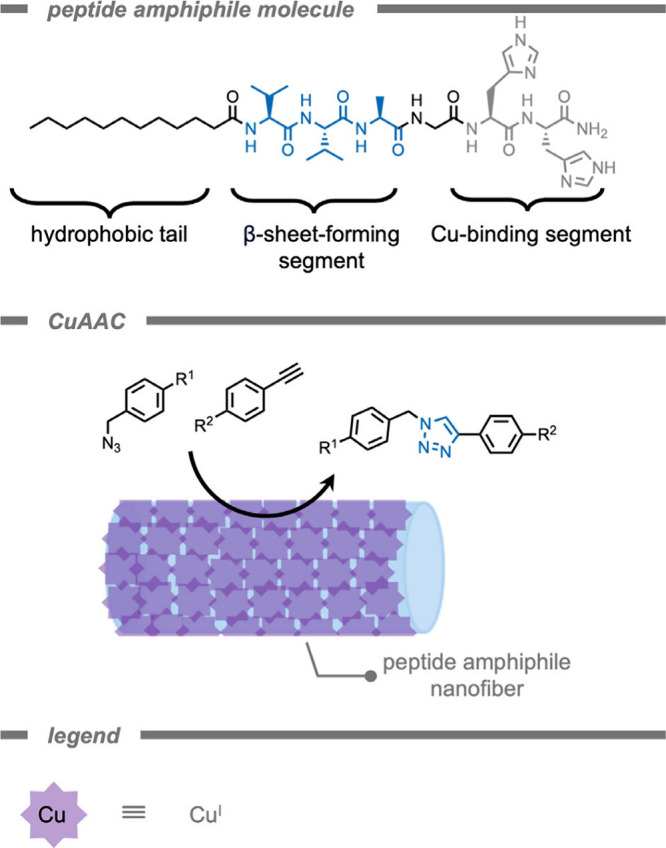
A supramolecular peptide amphiphile-based nanofiber catalyst for bioorthogonal Cu^I^-catalyzed [3+2] azide–alkyne cycloaddition.^155^

The use of Pd in biological systems allows for a wide scope of coupling and cleavage reactions.^158^ Whereas Pd has been reported as an efficient catalyst for coupling reactions in organic solvents, Pd on its own is often limited in its catalytic stability in biological conditions. To tackle this challenge, Palmans and co-workers^159^ incorporated a Pd^II^ complex into a synthetic polymer scaffold to protect the Pd from the cellular environment (Figure 6). This scaffold was generated from single chain polymeric nanoparticles (SCNPs), which form from an individual polymer chain containing hydrophobic and hydrophilic groups. These nanoparticles (NPs) collapse in aqueous environments into a compact structure.^160^ SCNP scaffolds are not only utilized for catalyst protection but can also preorganize substrates with the active site in the NP’s hydrophobic microenvironment. After complexation of Pd^II^ to the polymers, Pd^II^ was reduced to Pd^0^ by carbon monoxide (CO) to form Pd^0^@SCNPs.^161^ As this reduction is not bioorthogonal, these particles would need to be prepared in a stable manner before any potential administration to a biological system. The authors demonstrated the catalytic ability of Pd^0^@SCNPs for depropargylation reactions, leading to fast kinetics due to preorganization effects. Bare Pd^0^ NPs did not show any reactivity under these conditions. Also, intramolecular Heck coupling and Suzuki–Miyaura coupling reactions were performed in water, leading to quantitative yields to the desired product. For a comprehensive review on the effect of polymeric scaffold materials on TMC stability and activity for bioorthogonal catalysis, the reader is referred to Palmans and co-workers.^162^

**Figure 6:**
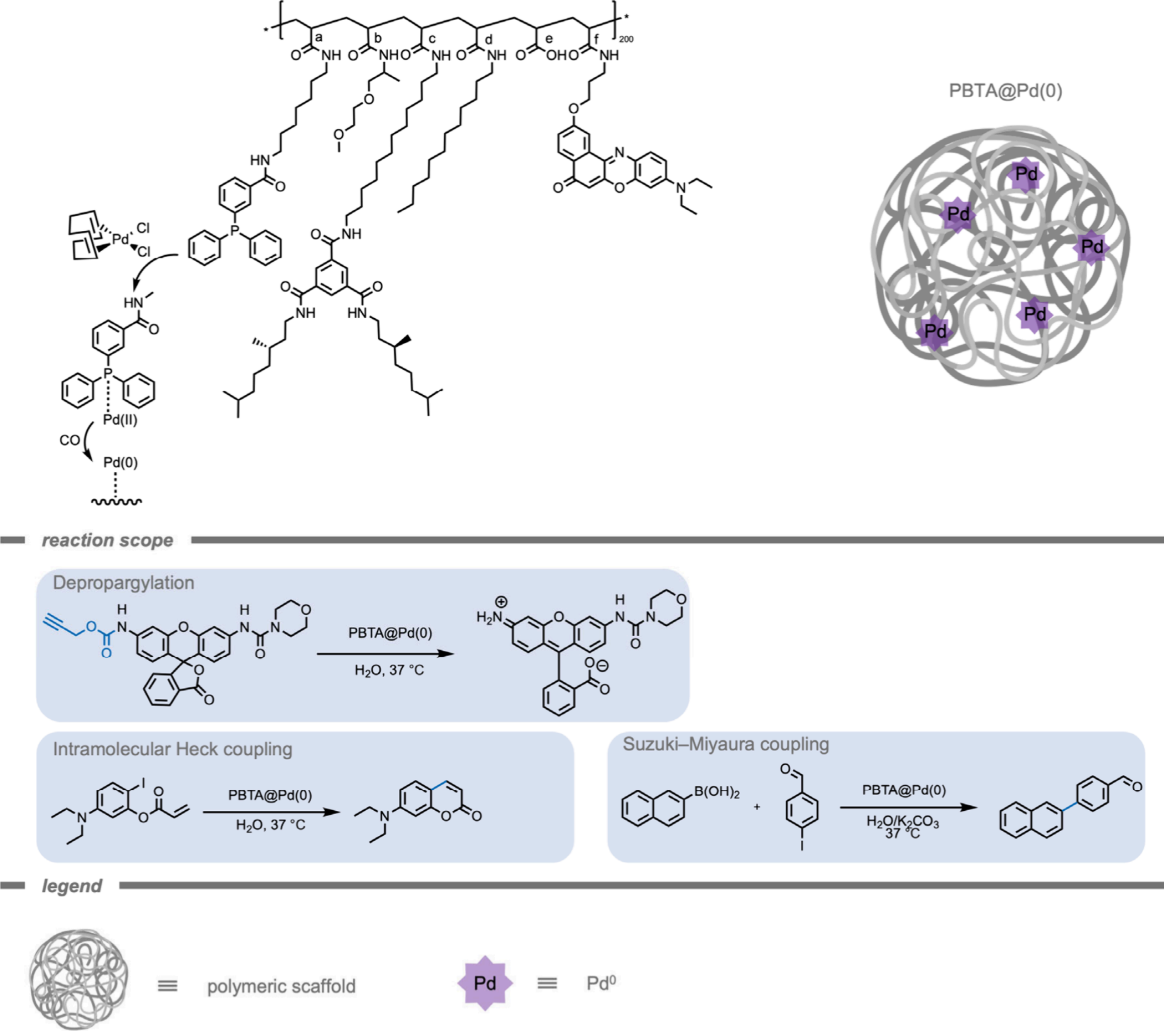
A single chain polymeric nanoparticle scaffold to protect a Pd(0) catalyst from the biological environment.^159^

In 2023, Hueso, Sebastian, Santamaria, Unciti-Broceta, and co-workers^163^ reported encapsulated AuPd-alloy NPs for the depropargylation of paclitaxel inside tumor cells. AuPd NPs exhibited the highest catalytic activity for the deprotection of propargyl-protected rhodamine 110 in PBS and serum at 37 °C, compared to NPs consisting of Au, Pt, and Ru. However, cancer cells pretreated with AuPd NPs were unable to convert propargyl-protected paclitaxel into its toxic form, indicating that these NPs were unable to enter the cells or were rapidly deactivated in the cellular environment. The encapsulation of AuPd NPs within poly­(lactic-*co*-glycolic acid) resulted in improved cellular uptake and efficient catalytic deproparylation of paclitaxel, which ultimately led to strong inhibition of cancer cell proliferation.

A polymer-based supramolecular catalytic system was reported by Rotello and co-workers^164^ for the treatment of bacterial biofilms (Figure 7). The authors encapsulated iron­(III) tetraphenylporphyrin chloride ([Fe­(TPP)­Cl]) TMCs in a hydrophobic pocket of the self-assembling polymer scaffold to solubilize and stabilize the catalyst. Moreover, the overall cationic charge of the NP could facilitate challenging penetration into bacterial biofilms. [Fe­(TPP)­Cl] was chosen as the catalyst due to its established catalytic efficiency for the reduction of aryl azides to the amine product in the presence of endogenous thiols.^165^ The supramolecular catalytic system demonstrated superior activity for probe deprotection in bacterial biofilms compared to free catalyst. In the presence of encapsulated [Fe­(TPP)­Cl], pro-resorufin was deprotected in biofilms as indicated by bright fluorescence in the red channel. Biofilms treated with pro-dye and free [Fe­(TPP)­Cl] exhibited negligible fluorescence. Free [Fe­(TPP)­Cl] only penetrated the biofilm in low concentrations, indicating that the polymer scaffold acted as a vehicle to load hydrophobic TMCs into biofilms.

**Figure 7:**
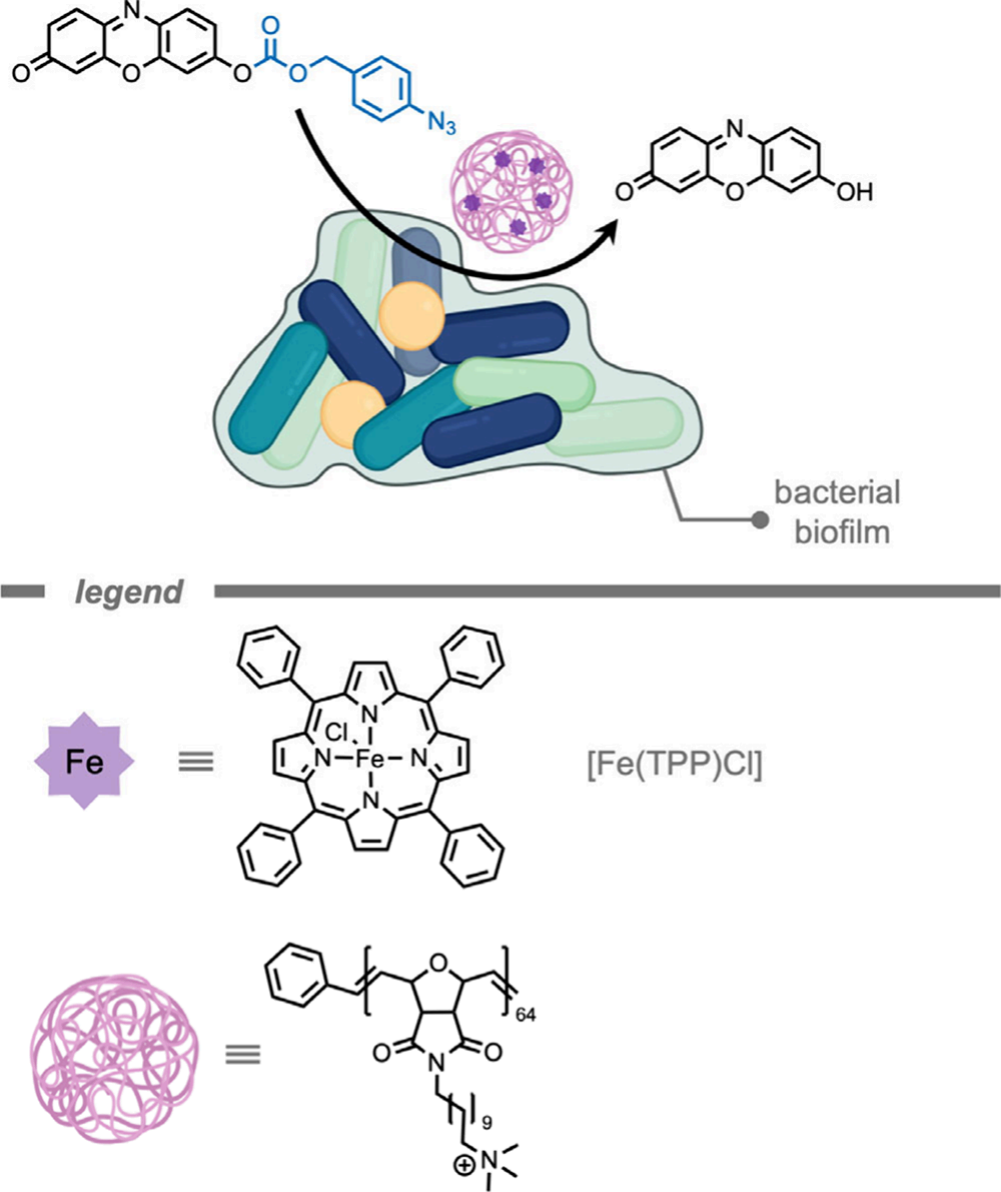
A supramolecular polymer-based catalytic system for the treatment of biofilms while stabilizing the [Fe­(TPP)­Cl] catalyst in a hydrophobic pocket.^164^

Rotello and co-workers^166^ also successfully demonstrated the use of polymer-based supramolecular catalytic systems for the efficient activation of anticancer drugs *in vitro* (Figure 8). Their system outperformed free Cp*Ru­(cod)Cl (Cp* = pentamethylcyclopentadienyl, cod = cycloocta-1,5-diene) TMCs 2-fold in PBS and 3-fold in 10% serum. This improved performance was attributed to the hydrophobic interior of the polymeric scaffold, which provided a protective environment to shield the TMCs from deactivation by serum proteins. This work emphasizes the potential of these polymer-based supramolecular systems to stabilize and enhance catalytic performance in complex biological environments and underlines their potential in targeted anticancer therapy.

**Figure 8:**
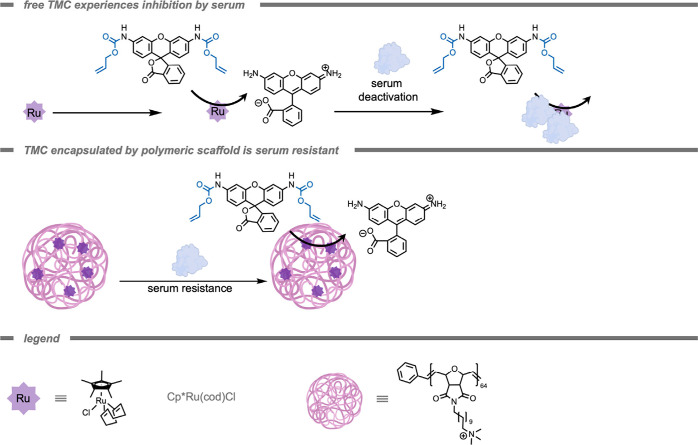
A polymer-based supramolecular catalytic system for serum-resistant probe deprotection and anticancer drug activation *in vitro.* ^166^

In 2021, Zimmerman and co-workers^167^ also highlighted the impact of a polymeric supramolecular scaffold on catalytic efficiencies (Figure 9). Their study demonstrated a significant increase in the activity of Ru-based catalysts for allyl carbamate deprotections when encapsulated in a polymeric scaffold. The encapsulation enhanced the Ru-L9 catalyst’s initial rate from 6 nM/s to 100 nM/s, with the conversion increasing from 30% to 84%. Beyond this, they reported the versatility of their scaffold by also improving the catalytic activity of the CuAAC reaction, through encapsulation of a Cu catalyst. The noncovalently cross-linked SCNP allowed for selective binding of both substrates and catalysts within its structure, significantly increasing local concentrations of reaction components, and improving the reaction kinetics as a result. This increase in reaction rate was especially pronounced for Cu catalysts with hydrophobic or anionic ligands. For instance, while the free Cu-L5 complex exhibited a minimal initial rate of 0.8 nM/s and a conversion of just 1%, encapsulation within the SCNP significantly increased these values to 88 nM/s and 90%, respectively. In conclusion, these examples show that polymeric supramolecular scaffolds can perform as versatile platforms to stabilize non-natural reaction components like TMCs under biologically relevant conditions to enhance their activity in bioorthogonal reactions.

**Figure 9:**
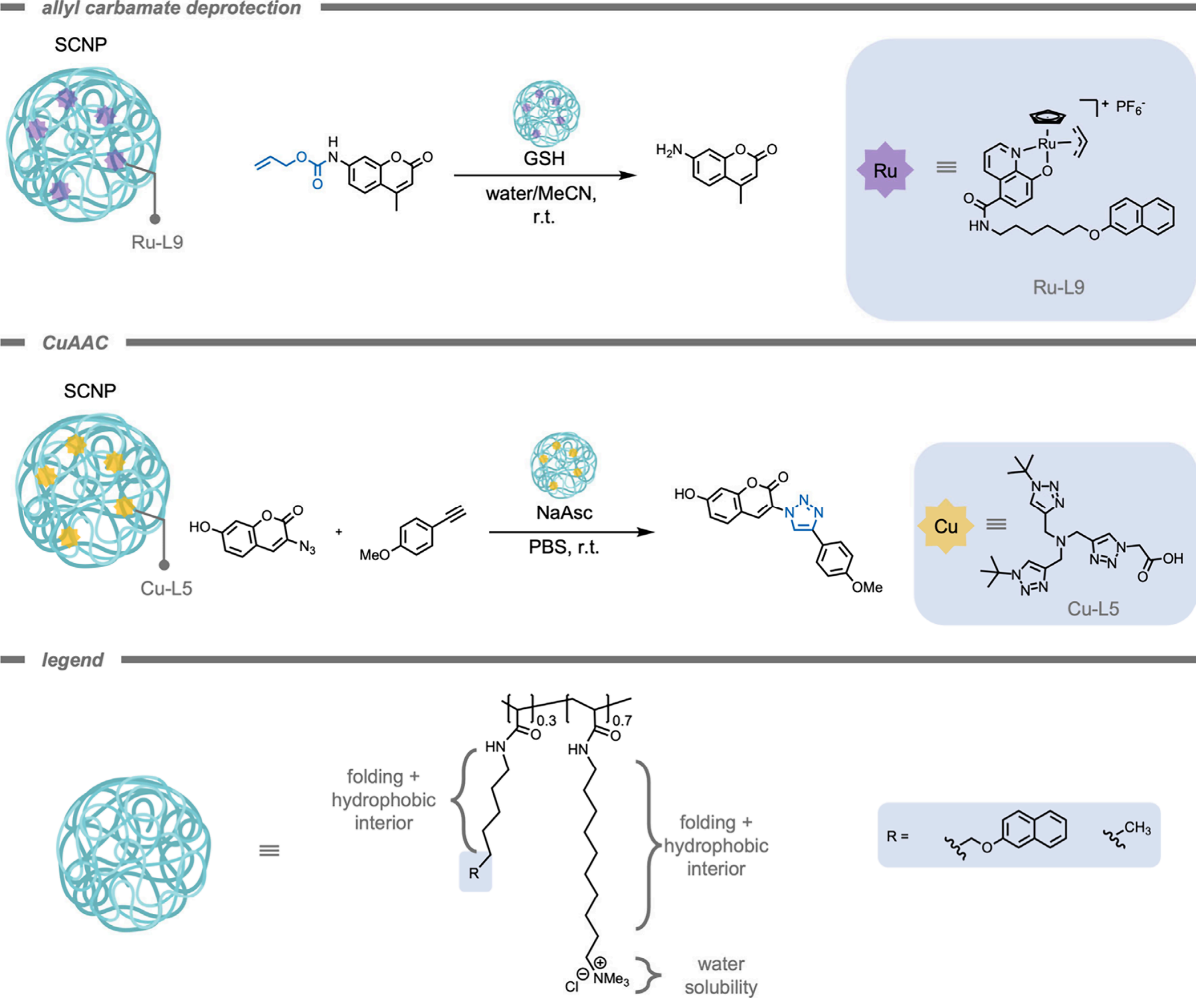
A single chain polymeric nanoparticle-based catalytic platform for Ru-catalyzed allyl carbamate deprotection or Cu^I^-catalyzed [3+2] azide–alkyne cycloaddition under biologically relevant conditions.^167^

### 3.2 Gold Nanoparticles to Protect Transition Metal Catalysts

AuNPs have been applied in diagnostics,^168^ food safety,^169^ environmental biosensing,^170^ as well as *in vitro* and *in vivo* catalysis.^171^ In the context of this review, surface-modified AuNPs have been used as a scaffold to facilitate TMC-mediated catalytic reactions in the protected environment on the surface. Stabilizing surface coatings are required for biorelevant applications, since inorganic NPs are colloidally unstable and tend to form large agglomerates.^172^ Since the catalytic reaction of AuNPs occurs on the surface, the effects of surface modification on catalytic activity and cellular uptake have been investigated.^173^ For example, in 2020 Rotello and co-workers^152^ demonstrated the protection of TMCs in self-assembled monolayer-coated AuNPs (Figure 10). The AuNP scaffolds consist of three key structural features: (i) a hydrophobic aliphatic monolayer, (ii) a hydrophilic tetra ethylene glycol spacer, and (iii) a terminal quaternary ammonium to improve solubility in aqueous media. The authors stated that encapsulation of a Cp*Ru­(cod)Cl catalyst in the hydrophobic environment preserved catalytic activity for the deprotection of the allyl carbamate group at various pH values and in complex biological media, whereas the free catalyst demonstrated a drop in activity when varying the pH from 4.1 to 7.4. In aqueous media (pH 7.4), the free TMC was completely deactivated whereas ∼60% of catalytic activity was retained for the encapsulated TMCs after 4 h.

**Figure 10:**
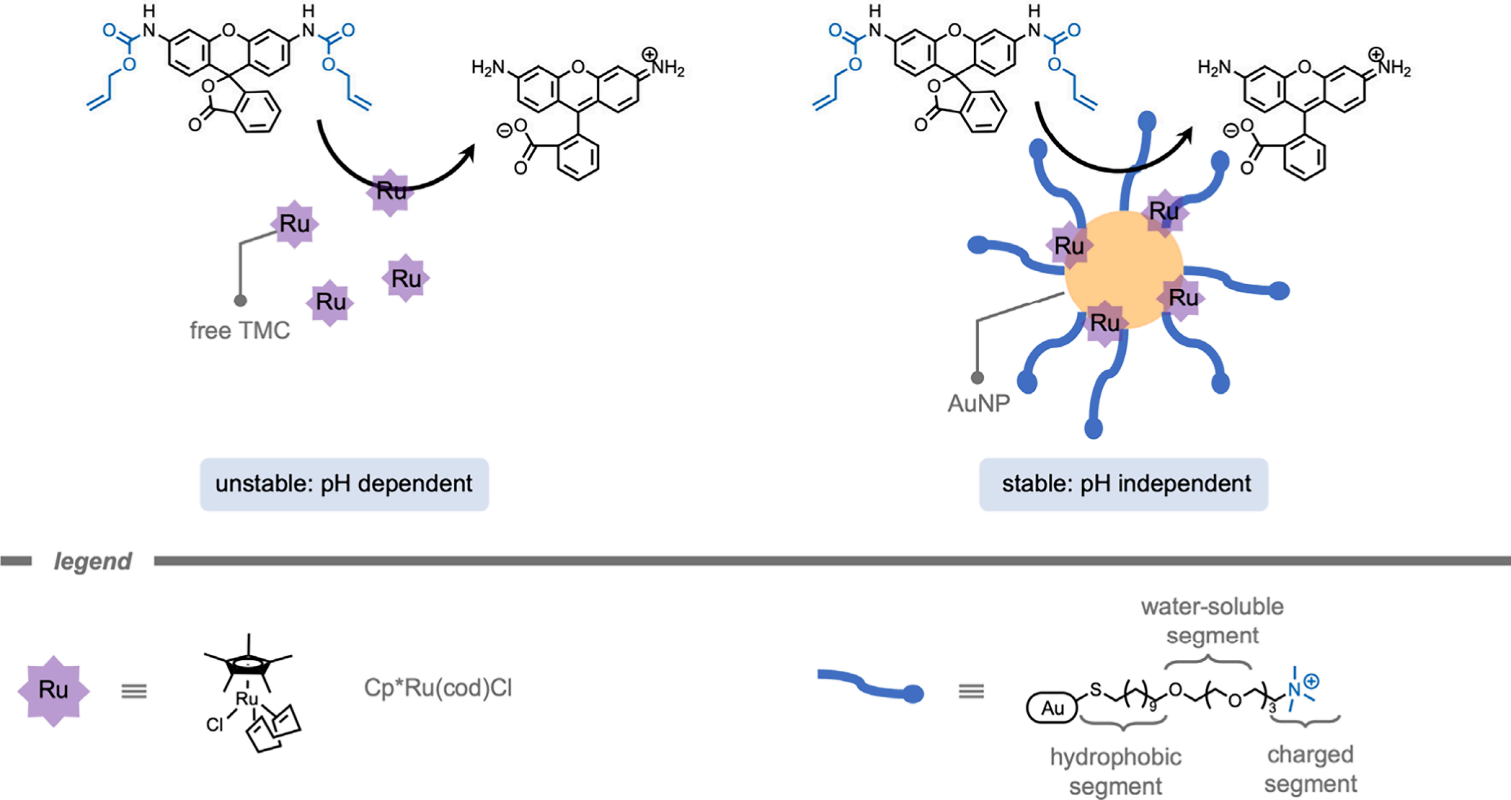
Protection of transition metal complexes in the hydrophobic environment of self-assembled monolayer-coated gold nanoparticles.^152^

In 2024, Rotello and co-workers^174^ demonstrated the use of TMCs encapsulated in monolayer-coated AuNPs for macrophage repolarization through prodrug activation (Figure 11). Macrophage repolarization (from M2 to M1) is studied as an immunotherapeutic approach to cancer treatment as several cancer types can hijack macrophages to facilitate their proliferation, angiogenesis, invasion, as well as therapy resistance. The Pd­(dppf)­Cl_2_ ([1,1′-bis­(diphenylphosphino)­ferrocene]­dichloropalladium­(II)) catalysts were encapsulated into the hydrophobic layer of the NP. The hydrophobicity of the ligands kept the TMCs encapsulated in the hydrophobic layer for an increased time. The authors attributed the internalization of the resulting supramolecular NPs by macrophages to endocytosis. Subsequently, the prodrug was deprotected inside the macrophages. The deprotected imiquimod drug re-educated M2-like macrophages to the anticancer M1-type. A key aspect of macrophage-mediated anticancer activity is their engulfment of cancer cells through phagocytosis, and re-educated macrophages should exhibit enhanced phagocytosis activity. Macrophages treated with pro-imiquimod and Pd­(dppf)­Cl_2_@AuNP demonstrated a 4-fold increase in phagocytosis of green fluorescent protein (GFP)-expressing osteosarcoma cancer cells, reaching the same level as administration of imiquimod. Current therapeutic use of imiquimod requires topical delivery as systemic treatment results in nonspecific inflammation reactions. According to the authors, their prodrug design could help minimize off-target effects, enabling an intravenous injection as administration for *in vivo* studies. The Pd­(dppf)­Cl_2_@AuNPs (400 nM, containing 11.6 μM Pd catalyst) were much less immunogenic compared to the free Pd catalyst (11.6 μM) within 24 h, presumably because of the shielding of the catalyst from the environment. Moreover, the Pd­(dppf)­Cl_2_@AuNP-based deprotection of pro-imiquimod resulted in approximately 4-fold elevated phagocytosis of cancer cells compared to controls, reflecting the engulfment of cancer cells by re-educated macrophages.

**Figure 11:**
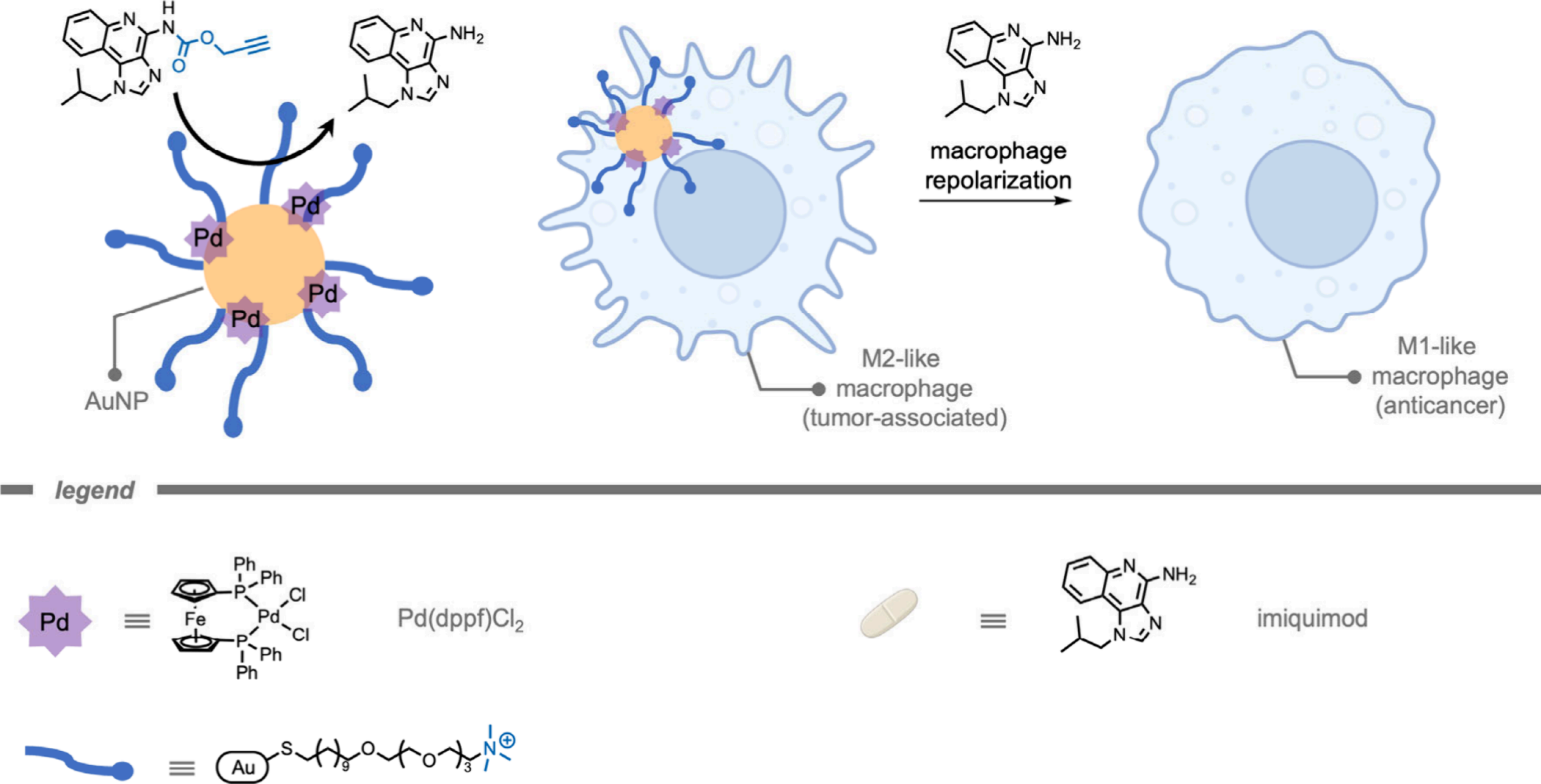
A monolayer-coated gold nanoparticle with encapsulated transition metal complexes for prodrug activation in M2-like macrophages.^174^

### 3.3 Miscellaneous Supramolecular Strategies to Protect Transition Metal Complexes

Next to the incorporation of TMCs in rigid scaffolds (e.g., AuNPs, polymers), the development of artificial membraneless organelles was recently demonstrated for the incorporation of hydrophobic TMCs. Although challenging due to the hydrophilic nature of polyelectrolyte-based coacervates, Landfester, Caire da Silva, and co-workers^175^ developed dipeptide-based coacervates. These coacervates possessed enhanced stability in and compatibility with biological environments, while also creating a hydrophobic microenvironment (Figure 12). Coacervates are spontaneously formed through liquid–liquid phase separation. The dipeptide-based coacervates are based on the self-association of polypeptides, which occurs through multivalent, weak interactions (π–π, cation−π, and hydrogen bonding). As coacervates are liquid phases without a membrane, free diffusion of reaction components can occur.^176^ The authors demonstrated the effect of the hydrophobic microenvironment on catalyst activity for the deprotection of allyl carbamate-protected rhodamine 110 in aqueous solution using [Cp*Ru­(cod)­Cl]. After ∼20 min, the encapsulated TMCs demonstrated a ∼10-fold increase in fluorescence compared to the reaction carried out with the free TMCs. Also, the *in vitro* catalysis in HeLa cells demonstrated a similar trend, with increased fluorescence for the encapsulated TMCs.

**Figure 12:**
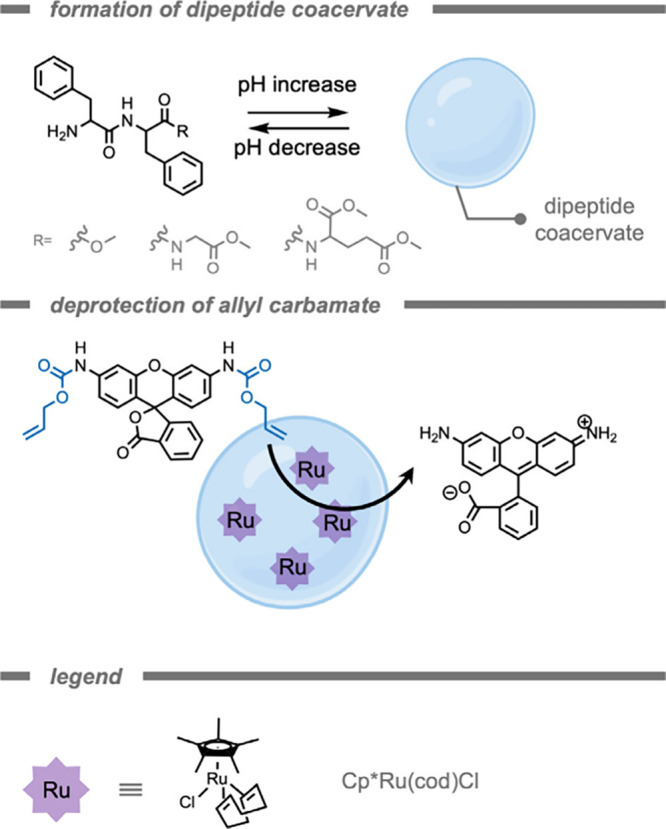
Dipeptide coacervate droplet for bioorthogonal probe deprotection while protecting the Ru transition metal complex in a hydrophobic pocket.^175^

An alternative strategy to the use of NP scaffolds was reported in 2023 by Do and co-workers^177^ (Figure 13). They created a macrocyclic catalyst for the reduction of C=O groups. This catalyst contained two Cp*Ir units with backbone amide functional groups that were capable of binding external Lewis acids. The combination of the macrocyclic catalyst with alkali ions led to the formation of organized supramolecular structures that exhibited improved tolerance toward GSH (up to 1 mM) and improved selectivity toward small aldehyde substrates. When the alkali ions were replaced with noncoordinating tetraalkylammonium cations, no higher-order structures were observed, and the catalysts were unable to discriminate between sterically hindered and unhindered aldehyde substrates. Preliminary cell studies indicated an IC_50_ of 85 μM in NIH-3T3 cells, underlining the importance of performing catalysis at low catalyst concentrations. This work suggests that *in situ* coordination self-assembly could be a promising strategy to enhance selectivity for bioorthogonal reactions.

**Figure 13:**
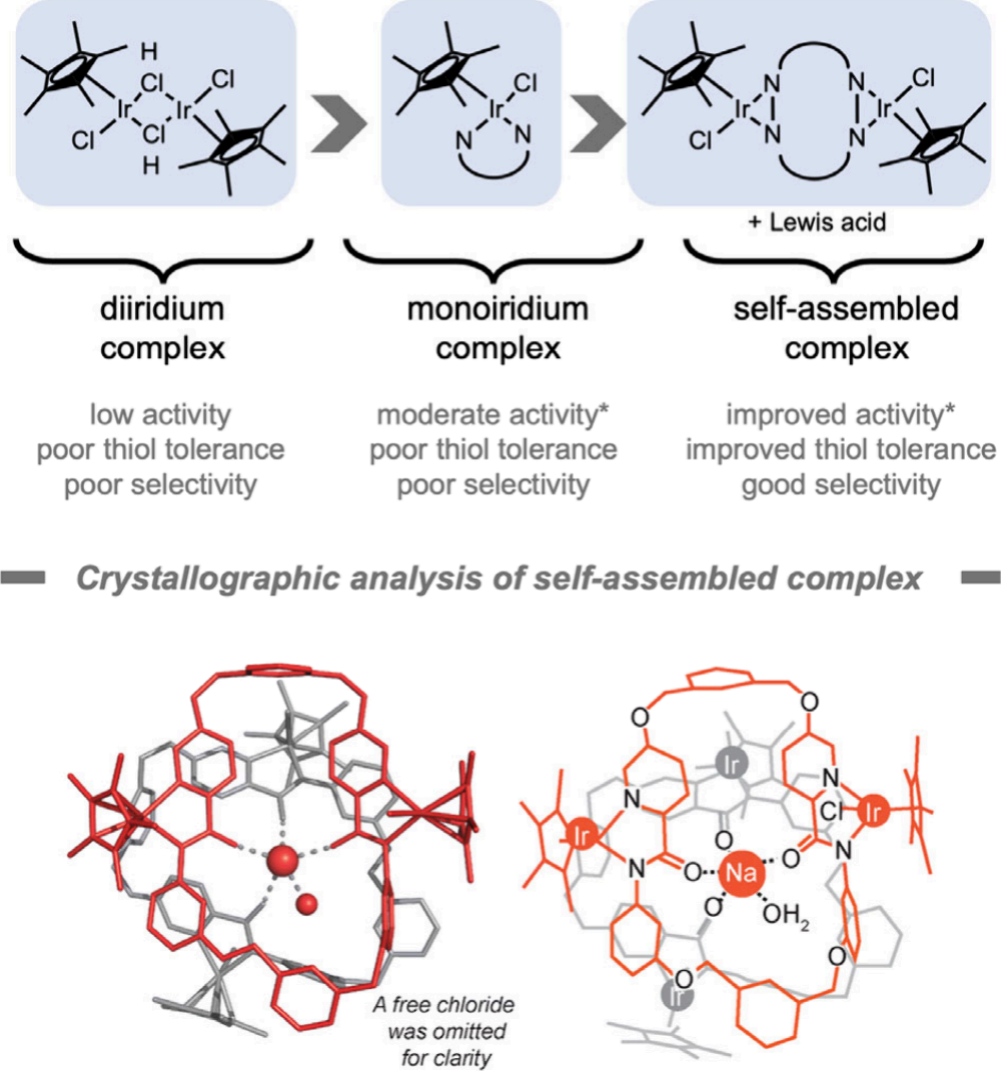
Optimization of iridium-containing catalysts for bioorthogonal reactions.^177^ *Under biorelevant conditions. Reproduced in part with permission from ref 177. Copyright 2023, Royal Society of Chemistry.

Reek and co-workers^178^ demonstrated the use of supramolecular cages—sometimes referred to as metal–organic cages (MOCs)—to improve the reactivity of an Au TMC under biorelevant conditions (Figure 14). MOCs are distinct, individual coordination complexes in which metal ions or clusters are interconnected by organic linker molecules.^179^ Rational design of the building blocks can lead to single-unit cage structures with different geometries depending on the symmetry of the building blocks used. The authors were inspired by work of Tanaka and co-workers^180^ in which docking of a NHC–Au­(I) complex (NHC = N-heterocyclic carbene ligand) inside the hydrophobic cavity of albumin—to yield an artificial metalloenzyme—prevented catalyst poisoning by GSH leading to improved reactivity for anticancer drug synthesis. To generate a physical barrier between the gold center and the surrounding environment, the authors selected a small tetrahedral K_12_[Ga_4_L_6_] MOC developed by Raymond and co-workers.^181^ Once catalysts are encapsulated inside these cages, they are known to be able to perform size-selective catalysis in which small molecules may still access the active site, but larger biomolecules are hindered from reaching the Au center. The authors encapsulated a catalytically active Au TMC, [Au­(PMe_3_)]^+^ inside the negatively charged cage. Almost identical yields for an intramolecular hydroarylation reaction were obtained in water for free TMC [Au­(PMe_3_)­Cl] and encapsulated complex [Au­(PMe_3_)]^+^⊂cage (69% and 70%, respectively). In the presence of biorelevant additives substantial reactivity remained for the complex only when encapsulated. When the reaction was performed in a mixture of phosphate-buffered saline (PBS)/water, the encapsulated TMC (yield 63%) outperformed the free TMC (yield 22%). The retained activity of the encapsulated TMC was attributed to the MOC structure preventing chloride coordination to the Au center. Moreover, addition of poisoning GSH (0.5 equiv) decreased the yield to a larger extent for free TMC (2%) than for the encapsulated complex (13%). The encapsulated complex also outperformed the free TMC in cell culture medium. This work demonstrated that catalyst protection can be achieved by encapsulation in a supramolecular MOC. According to the authors, further work should focus on improving the stability or activity of the complex [Au­(PMe_3_)]^+^⊂cage at low substrate concentrations (10 μM) as well as design a delivery route to deliver this noncell-permeable MOC.

**Figure 14:**
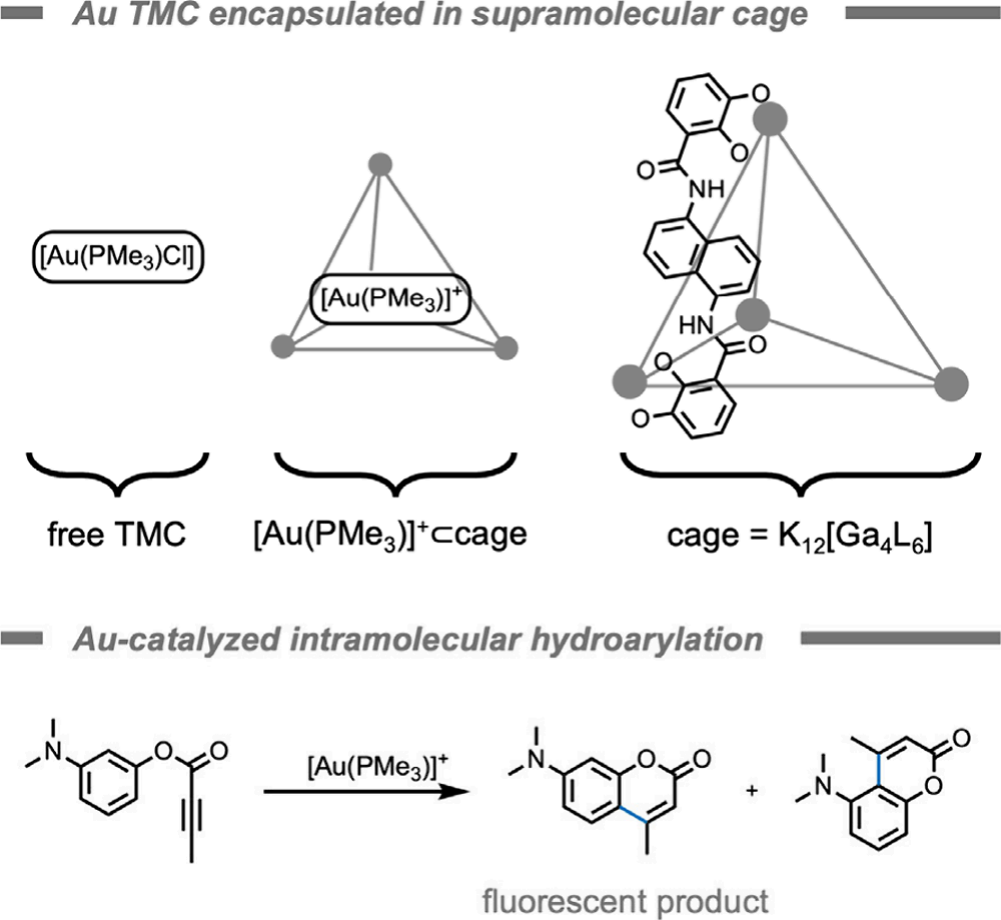
Encapsulation of an [Au­(PMe_3_)­Cl] transition metal complex within a K_12_[Ga_4_L_6_] supramolecular metal–organic cage enhances the activity for Au-catalyzed intramolecular hydroarylation reactions in the presence of biorelevant additives.^178^

In analogy to MOCs, metal–organic frameworks (MOFs), also consist of metal nodes connected by organic linkers, but instead of assembling into discrete complexes, extended coordination polymers can be formed.^179^ Assembly toward MOCs or MOFs can be guided by selection of the topicity (number of different coordination sites), denticity (number of donor interactions with a single metal node), and the relative orientation of the organic linker molecules.^179^ In the context of this review, MOFs have also been investigated as self-assembled objects to advance bioorthogonal reactions. Just like for MOCs, MOFs are structures that are based on reversible coordination chemistry,^182^ and the reversible nature of the coordination bonds can play a crucial role in their functionality within the context of bioorthogonal chemistry.^183^ Their highly tunable micro- or mesoporous structures allow for the encapsulation of specific substrates, and the reversible nature of their bonds may allow for selective delivery strategies. Moreover, it is possible to anchor isolated metal sides within the MOF structure to allow for controlled catalytic reactions. As such, MOFs represent an important class of supramolecular materials in the field of bioorthogonal chemistry. In 2023, Tian, Deng, and co-workers^184^ reported the deprotection of propargyl carbonate-protected RNA molecules within a Pd-containing MOF as a heterogeneous catalyst (Figure 15). The propargyl carbonate group was used to protect the −OH group in the ribose backbone of RNA from decomposition by RNA hydrolase as well as inhibit the activity of the RNA. The Pd-MOF-626 achieved complete conversion of the propargyl carbonate-protected RNA 90-fold more efficiently than free TMC Pd­(NO_3_)_2_. This effect was reported in terms of the required catalyst concentration to achieve 50% and 95% conversion, indicated as EC_50_ and EC_95_, respectively. The much lower EC_50_ and EC_95_ of the Pd-MOF-626 (51.84 nM, and 74.83 nM,) indicated higher catalytic efficiency compared to those of Pd­(NO_3_)_2_ (3252 nM, and 4488 nM).

**Figure 15:**
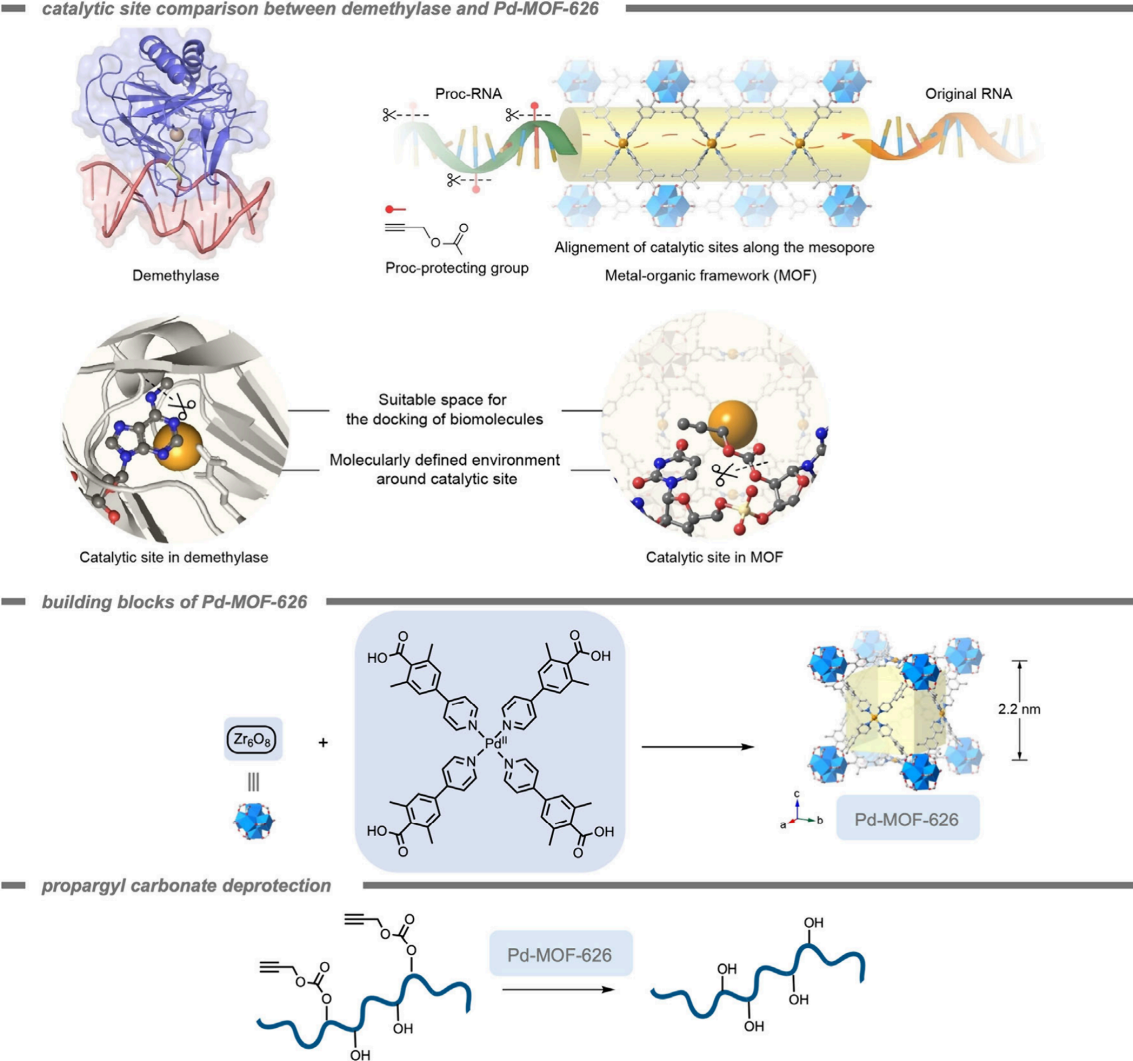
A Pd-containing metal–organic framework as a heterogeneous catalyst for deprotection of propargyl carbonate-protected ribonucleic acid (RNA) to restore their original activity and protect them from RNA hydrolase decomposition.^184^ Reproduced in part with permission from ref 184. Copyright 2023, Wiley Publishing.

Mascareñas, del Pino, and co-workers^185^ reported a core–shell zeolitic imidazolate framework-8 (ZIF-8) MOF-cloaked Pd NP for the performance of bioorthogonal depropargylation reactions. The MOF cloak preserved the catalytic site from deactivation and provided a diffusion-controlled access to the active site. Their catalytic system was demonstrated inside HeLa cells for the deprotection of bispropargyl carbamate-protected cresyl violet 5. Although the Pd NPs without the MOF shell also promoted the intracellular deprotection, the efficiency was considerably lowered.

Although the various supramolecular systems in this section have been demonstrated to improve the stability of incorporated non-natural TMCs, it is often unclear what changes occur to the different components of the NP or protective scaffold upon exposure to the biological environment. A certain degree of biotransformation upon *in vivo* administration might occur for the supramolecular systems described in this review, and more studies are required to investigate the effect of the biological system on the properties of the supramolecular particles.^186^ For a comprehensive overview on the biodegradability, integrity, and fate of NPs upon *in vivo* administration, the reader is referred to several reviews.^172,186^

## 4 Supramolecular Structures for Selective Release and Targeting

### 4.1 Host–Guest Chemistry

The active release of pharmaceutically relevant molecules by external stimuli has been a popular strategy to transport molecules through a biological environment while protecting them from the environment. This can be achieved through reversible encapsulation of the active compound within a supramolecular host, sometimes referred to as host–guest chemistry. Cyclodextrins (CDs), and cucurbit­[n]­urils (CB­[n]­s) are frequently used host molecules for this purpose. CB­[n]­s are a family of cyclic glycoluril-based molecular containers that can form stable complexes with a wide variety of guest molecules (e.g., drugs, dyes, peptides, perfluorinated hydrocarbons, and amino acids).^187^ CDs are a family of cyclic oligosaccharides often utilized in molecular recognition and material assemblies due to their superior molecular binding affinities in both aqueous solution and solid state.^188,189^ This encapsulation strategy can also be applied to catalysts or reagents, modulating their reactivity. Next to CDs and CB­[n]­s, a wide variety of host molecules that can bind guests have been reported in literature.^9,143,190–194^ Although many of these host–guest systems have been employed in biorelevant applications,^195^ in this section only those examples that involve selective release of components for bioorthogonal reactions will be discussed. In addition to the selective release of molecules, supramolecular systems can also be employed to selectively take up specific molecules in complex media. Supramolecular hosts (e.g., CB­[n]­s, CDs) have been demonstrated to form tight complexes with toxins or drugs *in vivo* and act as sequestration agents.^143^ Interestingly, supramolecular hosts have also been reported to turn the activity of proteins on and off.^196^ However, also these types of approaches are beyond the scope of the current review.

Host–guest chemistry can be utilized to control the reactivity of catalysts. In 2020, Eelkema and co-workers^197^ demonstrated the use of a supramolecular CB host to control the reaction rate of a catalyst (Cu^I^–NHC) in the CuAAC reaction (Figure 16). By placing a catalyst in a well-defined supramolecular environment, a second coordination sphere is generated to control the properties of the catalyst.^93,198^ In this example, the NHC ligand binds with high affinity to CB[7], encapsulating the Cu–NHC catalyst within the CB[7] host. As a result, the Cu^I^ center is not catalytically active. The authors attributed this effect to the inaccessibility of the Cu^I^ center to the substrates or its inability to form a catalytically active species from the precursor. However, when a competing guest was added that binds stronger to the host, the Cu catalyst was released and its catalytic activity was switched on (quantitative yield in 3 h compared to no observed conversion for CB[7] inhibited catalyst). By employing chemical signals and host–guest chemistry rather than light-triggered processes to control the activation of the CuAAC reaction, phototoxicity and side effects can be mitigated. These can arise in light-activated click reactions, since high-energy light can generate ROS, directly damage biomolecules, or activate other reaction pathways in complex media. The temporary deactivation of the Cu^I^ species through this supramolecular structure could also result in reduced toxicity of the Cu species, further reducing side effects.

**Figure 16:**
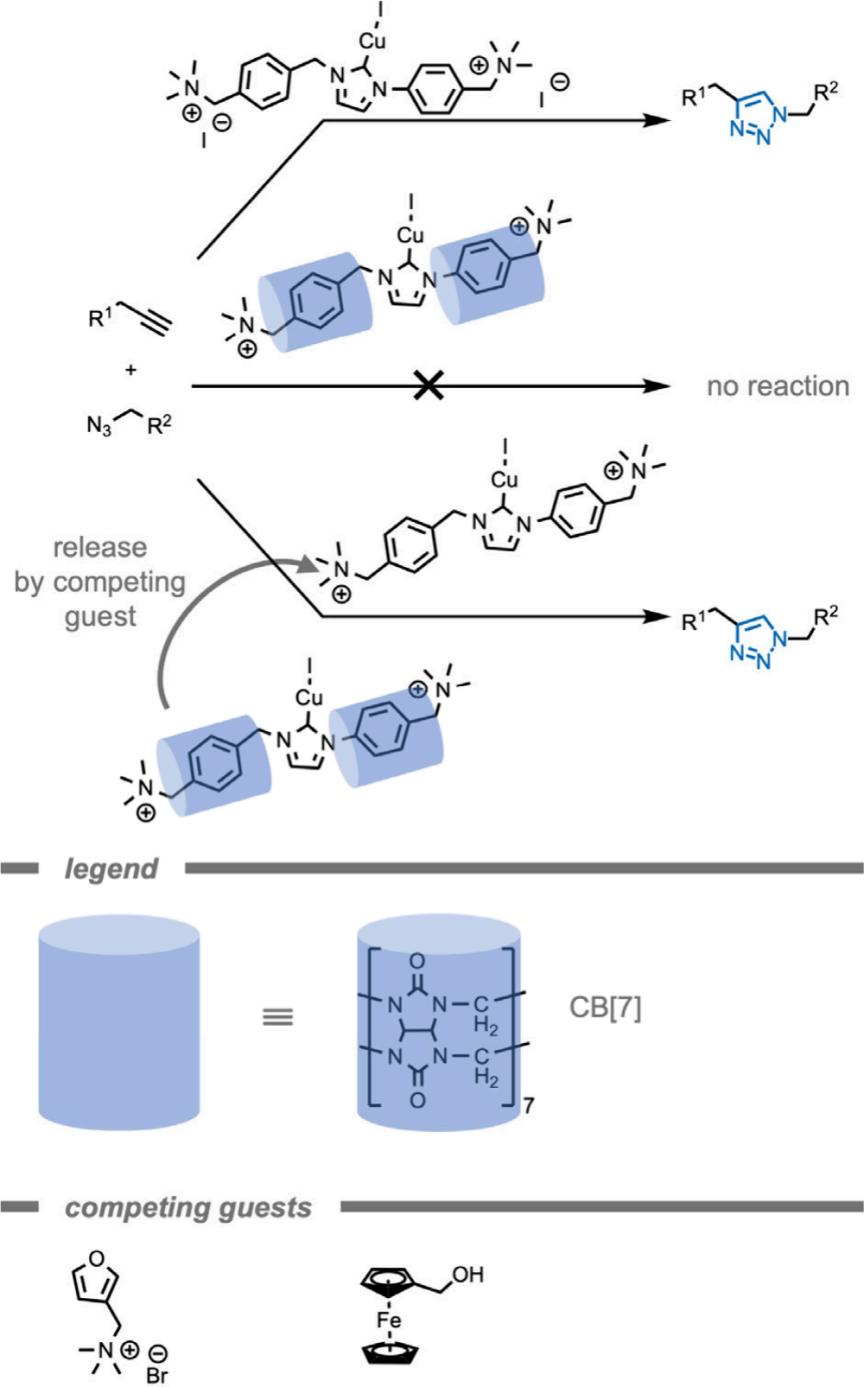
Structure of the curcurbit[7]­uril (CB[7]) host, a Cu^I^–(N-heterocyclic carbene) catalyst, and two chemical signal molecules.^197^ Subsequent release of the Cu^I^ catalyst from the CB[7] host by a strongly binding chemical signal molecule enables a high control over the catalytic activity.

The reactivity of tetrazine in IEDDA reactions has been mediated by *in situ* generation of tetrazine through photooxidation, photodeprotection, or electrochemical oxidation. However, these methods can suffer from phototoxicity and limitations in terms of *in vivo* operation.^199^ As an alternative method to control tetrazine reactivity, Liu and co-workers^200^ demonstrated the use of a supramolecular antinaphthotube host (Figure 17). These host molecules bind the tetrazine guest with binding affinities of *K*_d_ = 10^–7^ M, and the binding results in inhibition of the reactivity of the tetrazine. The tetrazines were released upon guest–guest exchange with 2-pyrimidines, which bind with roughly equal strength, and were therefore added in excess to fully release the tetrazine guest and restore the reactivity for the tetrazine-mediated IEDDA reaction. The authors also demonstrated this principle for tetrazine residues on proteins, extending the application from *in vivo* synthesis of small molecules to protein functionalization. The biocompatibility of this supramolecular tetrazine regulation was also shown in nude mice with subcutaneously grafted tetrazine-labeled HEK293 (human embryonic kidney) cells and decorated with antinapthotube. Subsequently, a TCO-labeled fluorophore was injected in the tail vein. From the obtained fluorescence after the IEDDA reaction, high supramolecular protecting and deprotecting efficiencies (94%, and 78%, respectively) were calculated.

**Figure 17:**
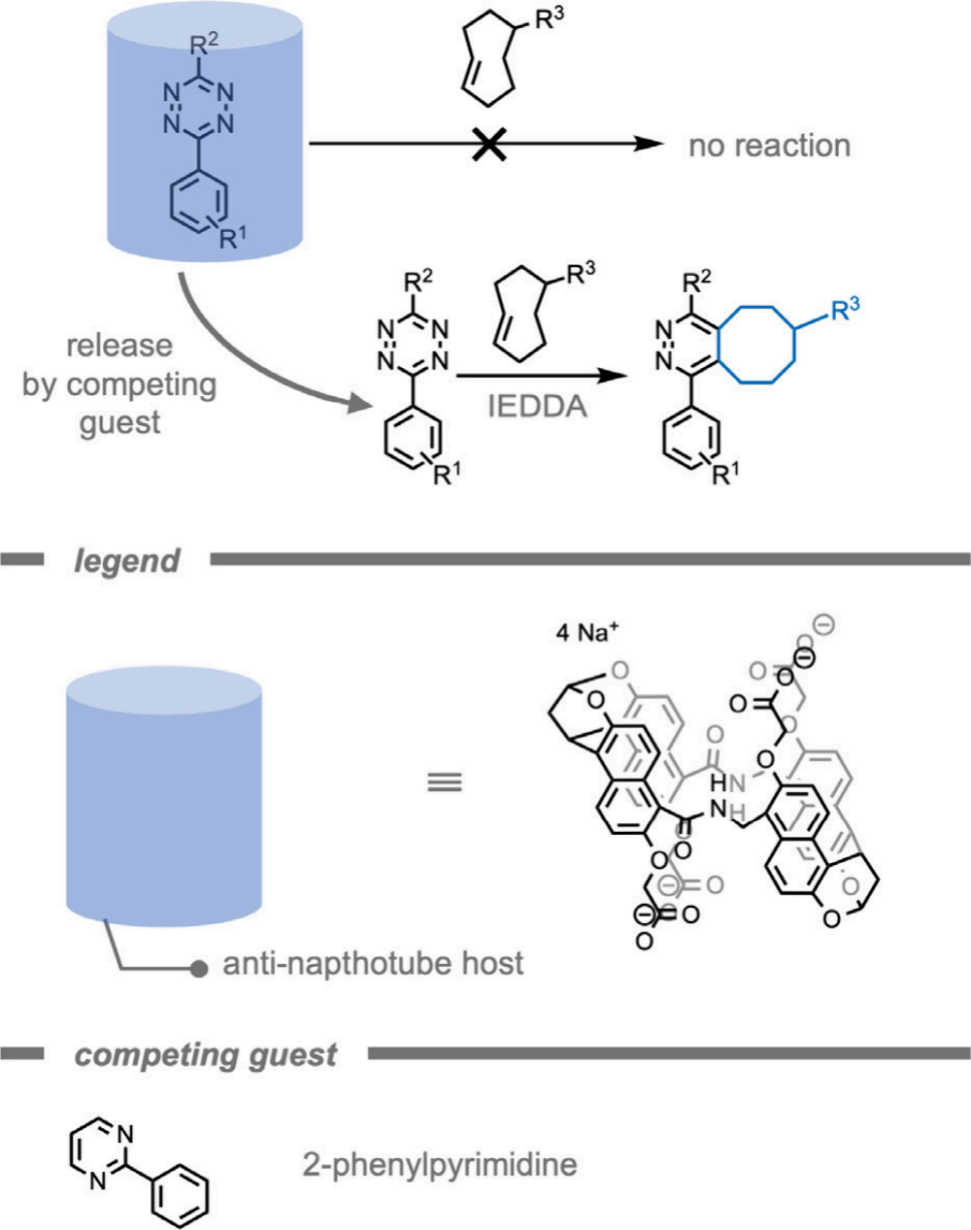
Structure and schematic depiction of the antinaphthotube host that strongly binds tetrazine, protecting it from further reactions.^200^ Subsequent guest–guest exchange of tetrazine by 2-phenylpyrimidines to restore the reactivity of the tetrazine for the tetrazine-mediated inverse-electron demand Diels–Alder reaction.^200^

### 4.2 Carrier Systems

To perform bioorthogonal reactions at the site of interest, carrier systems can be essential to deliver the reaction components as cargo to the proper location. Various natural extracellular vesicles have been investigated, such as exosomes, macrophages, microvesicles, and various types of cells.^62^ These systems can prolong the blood circulation time, have generally low immunogenicity, and have inherent cell-targeting abilities. Synthetic vesicles created through supramolecular assembly (e.g., liposomes, dendrimersomes, polymersomes, and lipid NPs) are inspired by these natural extracellular vesicles and can serve as confined environments for supramolecular catalysis.

Living stem cells have been demonstrated as effective carriers of TMCs. One challenge is to effectively attach catalysts to the surface of carrier cells while preserving the inherent catalytic activity and the ability of cells to interact with biological systems. Liu and co-workers^201^ have made use of layer-by-layer deposition of polymers on the cell surface through the strong noncovalent host–guest interactions between CB[7] and adamantane (Ada) (Figure 18). One polymer layer contained CB[7] groups, whereas the subsequent polymer layer contained Ada, creating a multilayer polymer system connected through CB[7]-Ada interactions. The resulting polymeric layer preserved the original cell characteristics (e.g., cell tropism, migration, and differentiation). Moreover, the authors incorporated a [Fe­(TPP)­Cl] into the polymeric coating on the stem cell by using a star copolymer (Ada–BASP). This copolymer adopts a spherical particle morphology with a hydrodynamic radius of 25 nm and can encapsulate bioorthogonal catalysts inside its hydrophobic compartments. A layer of Ada–BASP with [Fe­(TPP)­Cl] encapsulated was used in the layer-by-layer assembly methodology. The applicability of these systems was subsequently demonstrated for the catalytic reduction of an azide-protected rhodamine fluorophore. The encapsulated [Fe­(TPP)­Cl] TMC retained its catalytic activity within the system, highlighting the effectiveness of this technique to establish catalytic delivery systems.

**Figure 18:**
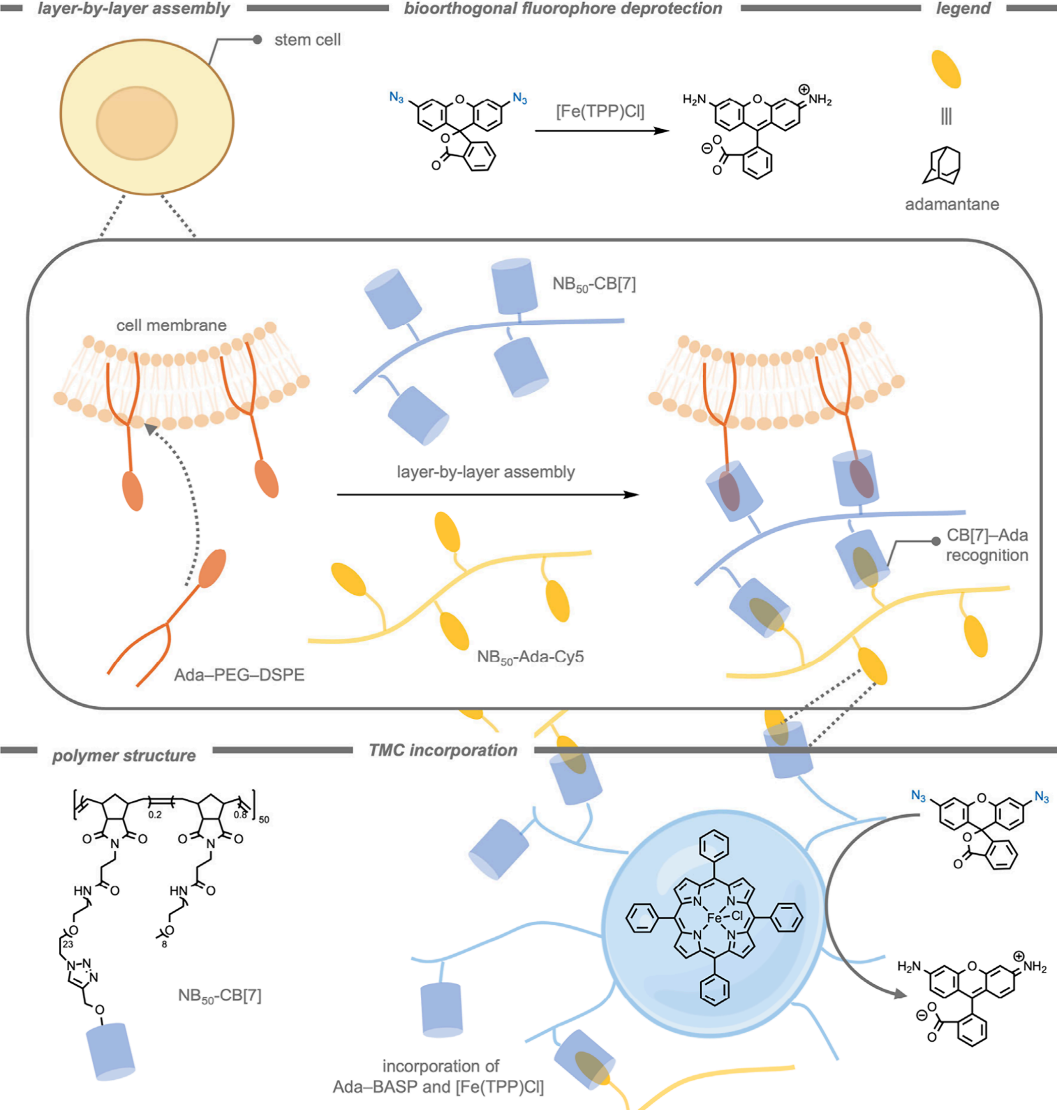
Layer-by-layer assembly of polymers on a cell surface through cucurbit[7]­uril–adamantane recognition, with [Fe­(TPP)­Cl] as bioorthogonal catalyst within the polymers for azide reduction.^201^

A significant challenge in the development of novel therapeutics is the balance between high efficacy and high safety. This challenge generally exists because most potent drug molecules are not only active at the site of interest but also elsewhere in the body, which typically results in serious side effects. To combat off-target activity of active drug molecules, various targeting strategies have been developed including the use of antibody–drug conjugates^202^ and the application of nanosized delivery vehicles (e.g., NPs, liposomes, and MOFs).^203^ A promising strategy involves the initial delivery of a prodrug to the site of interest, followed by a bioorthogonal prodrug activation on location.^61^

Santamaría, Unciti-Broceta, and co-workers^204^ investigated the applicability of cancer-derived exosomes loaded with ultrathin Pd nanosheets for bioorthogonal catalysis (Figure 19). Exosomes are naturally occurring supramolecular assemblies in the shape of membrane-enclosed vesicles. They are released by cells into the extracellular space where they facilitate the regulation of diverse biological functions and mediate the intercellular transfer of biomolecules. It is believed that exosomes play a crucial role in the tumor microenvironment in the communication between cancer cells,^205,206^ and therefore the authors investigated the possibility of using these exosomes as “Trojan horses” to deliver cargo into a specific type of cancer cell. Since the biochemical composition (i.e., the variety of proteins and receptors) of the exosome is reflective of the donor cell, they often target cells that are similar in composition to their origin cell.^207^ The Pd nanosheet-loaded exosomes proved to be highly catalytically active under biological conditions, preferentially target their progenitor cancer cell types, and were able to activate a propargyl ether-protected anticancer drug. The use of exosomes to deliver synthetic catalysts into specific cancer cells would allow for exosome-directed catalyst prodrug therapy in the future.

**Figure 19:**
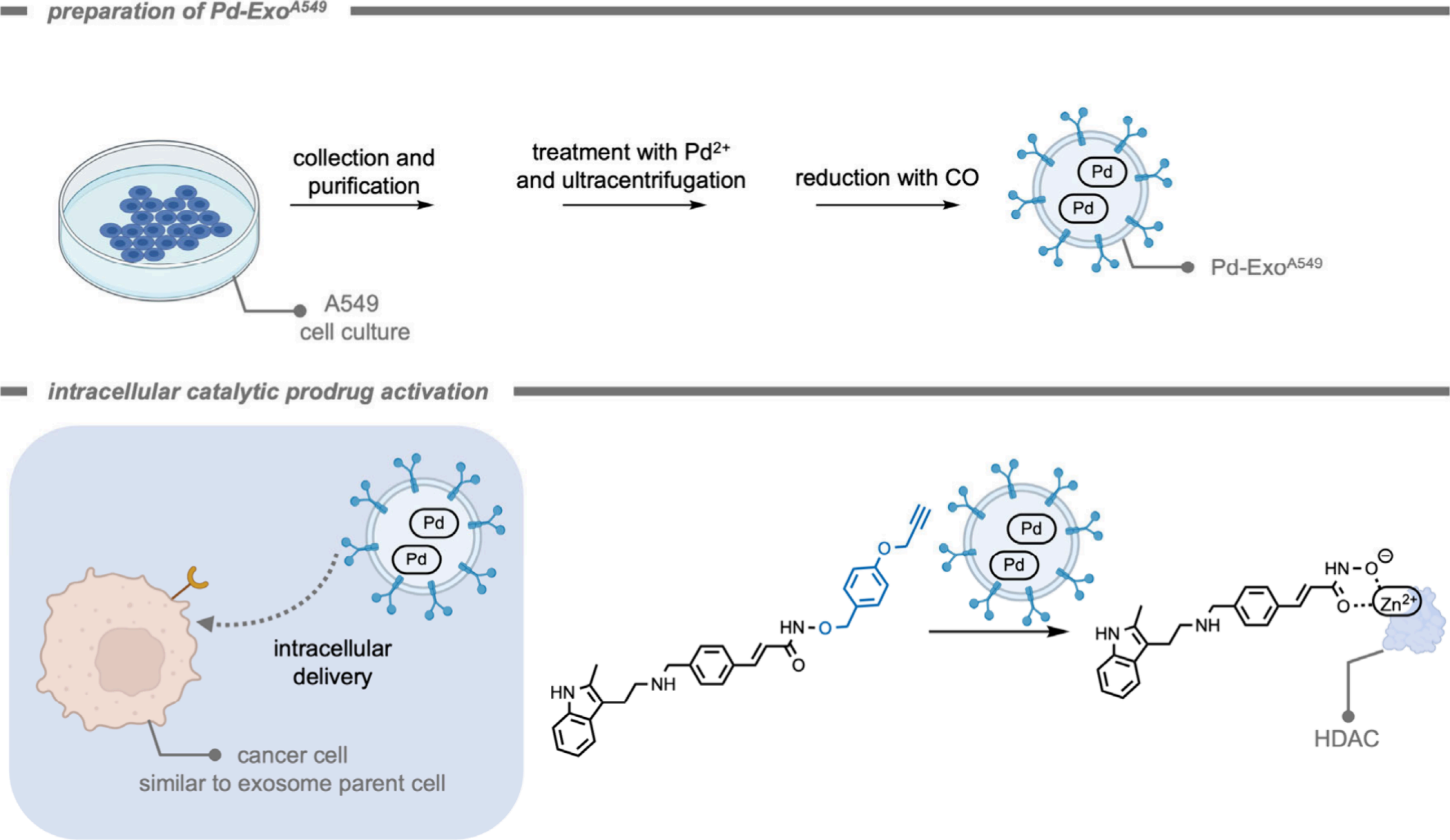
Exosomes loaded with Pd nanosheets for cell-specific targeting and intracellular catalytic deprotection of a pan-histone deacetylase (HDAC) inhibiting drug.^204^ The Zn-chelating hydroxamate group is essential for HDAC inhibition, and protection of this group inactivates the drug.

In 2024, Unciti-Broceta, Santamaría, and co-workers^208^ reported a catalytic system consisting of ultrathin catalytic Pd nanosheets inside cancer-derived extracellular vesicles. These extracellular vesicles acted as therapeutic vectors to carry out the deprotection of propargyl-protected paclitaxel in animal models (Figure 20). The proposed mechanism of the Pd-catalyzed step generates CO_2_ and hydroxyacetone as byproducts.^150,209^ Generally, as a consequence of their size, nanosized agents with longer circulation times leak preferentially into tumor tissue since the vasculature of tumors is characterized by leaky vessels and are then retained there as a result of poor lymphatic drainage.^210^ This passive targeting phenomenon is known as the enhanced permeability and retention (EPR) effect.^211^ The accumulation of extracellular vesicles inside tumor tissue after 48 h (2.25%) was significantly higher compared to controls of polyethylene glycol (PEG)-coated Pd nanosheets delivered solely through the EPR effect (0.91%). Moreover, a significantly lower accumulation of Pd was observed in liver tissue for the extracellular vesicles after 48 h and 1 week (70% and 68%, respectively) compared to the PEGylated Pd nanosheets (95% and 94%, respectively). This improved delivery to target tissues also translated to enhanced antitumoral efficiency *in vivo*. Tumor-bearing mice were treated with pro-paclitaxel and both Pd catalysts. The extracellular vesicles demonstrated improved tumor suppression in 21 days (∼120% tumor growth) compared to the PEGylated Pd nanosheets (∼200% tumor growth) and the pro-paclitaxel control (∼190% tumor growth). Although PEGylation can result in NPs with improved colloidal stability and increased *in vivo* retention times, cellular uptake is often reduced.^212^ Reduced cellular uptake has been attributed to the formation of a protein corona within the PEG layer, and these adsorbed proteins are therefore largely hidden from the interface.^213^ Instead of having interactions between the protein corona and cells, these PEGylated NPs mostly interact with cells through their PEG surfaces, leading to relatively low cellular uptake. For more insight on the effect of PEGylation on NPs in terms of cellular uptake and protein interactions, the interested reader is referred to Nienhaus, Parak, and co-workers.^213^

**Figure 20:**
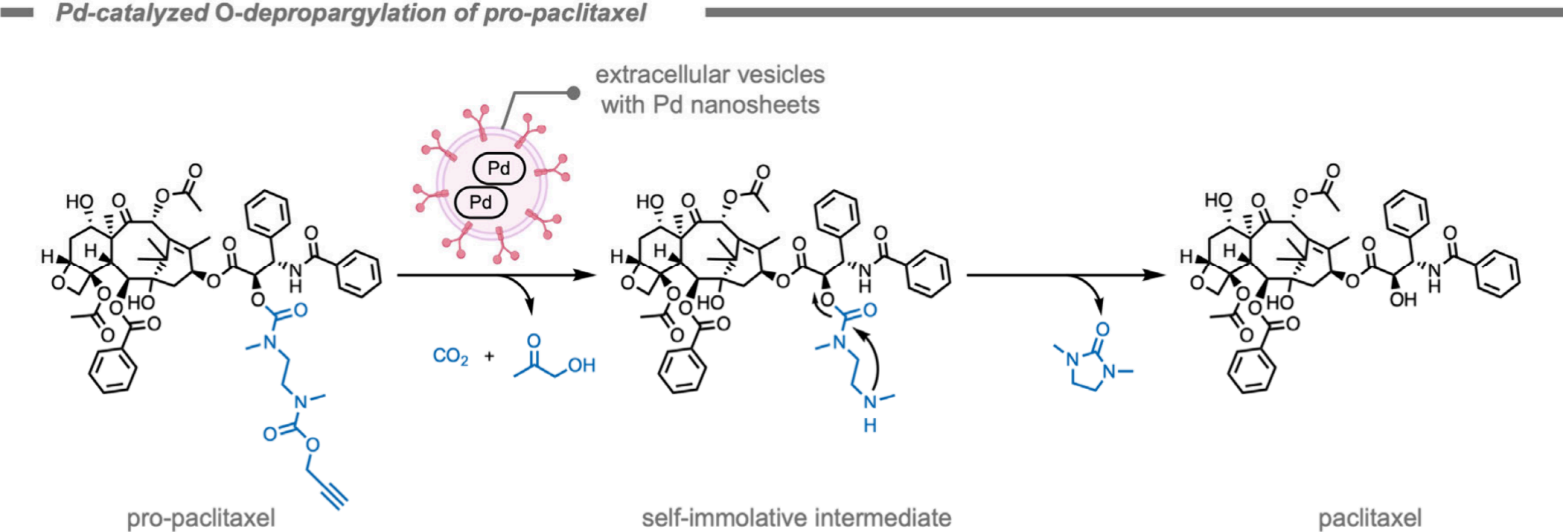
Pd-catalyzed *O*-depropargylation of pro-paclitaxel by extracellular vesicles loaded with Pd nanosheets to generate paclitaxel and the nontoxic byproduct 1,3-dimethyl-2-imidazolidinone.^208^

An example of cancer cell-targeted imaging was demonstrated in 2016 by Chen, Tseng, Lin, and co-workers,^214^ who introduced the use of supramolecular NPs for pretargeted oncologic positron emission tomography imaging through bioorthogonal reactions (Figure 21). These supramolecular NPs were self-assembled from four building blocks: a *trans*-cyclooctene (TCO)-grafted CD polyethylenimine polymer, polyethylenimine polymers, Ada-grafted polyamidoamine, and Ada-grafted PEG. The resulting NPs had a relatively uniform size of approximately 100 nm. Because the supramolecular NP is a dynamic structure, they can disassemble after accumulation to release the active TCO moiety. Subsequently, a small molecule containing a tetrazine moiety and a positron-emitting radioisotope (^64^Cu) was injected to react with the TCO moiety in the tumor. The TCO group can subsequently ligate to the tetrazine group on an injected radiolabeled reporter molecule (^64^Cu–tetrazine). After rapid clearance of the small radio-labeled molecule, high-contrast positron emission tomography imaging of the tumor was achieved.

**Figure 21:**
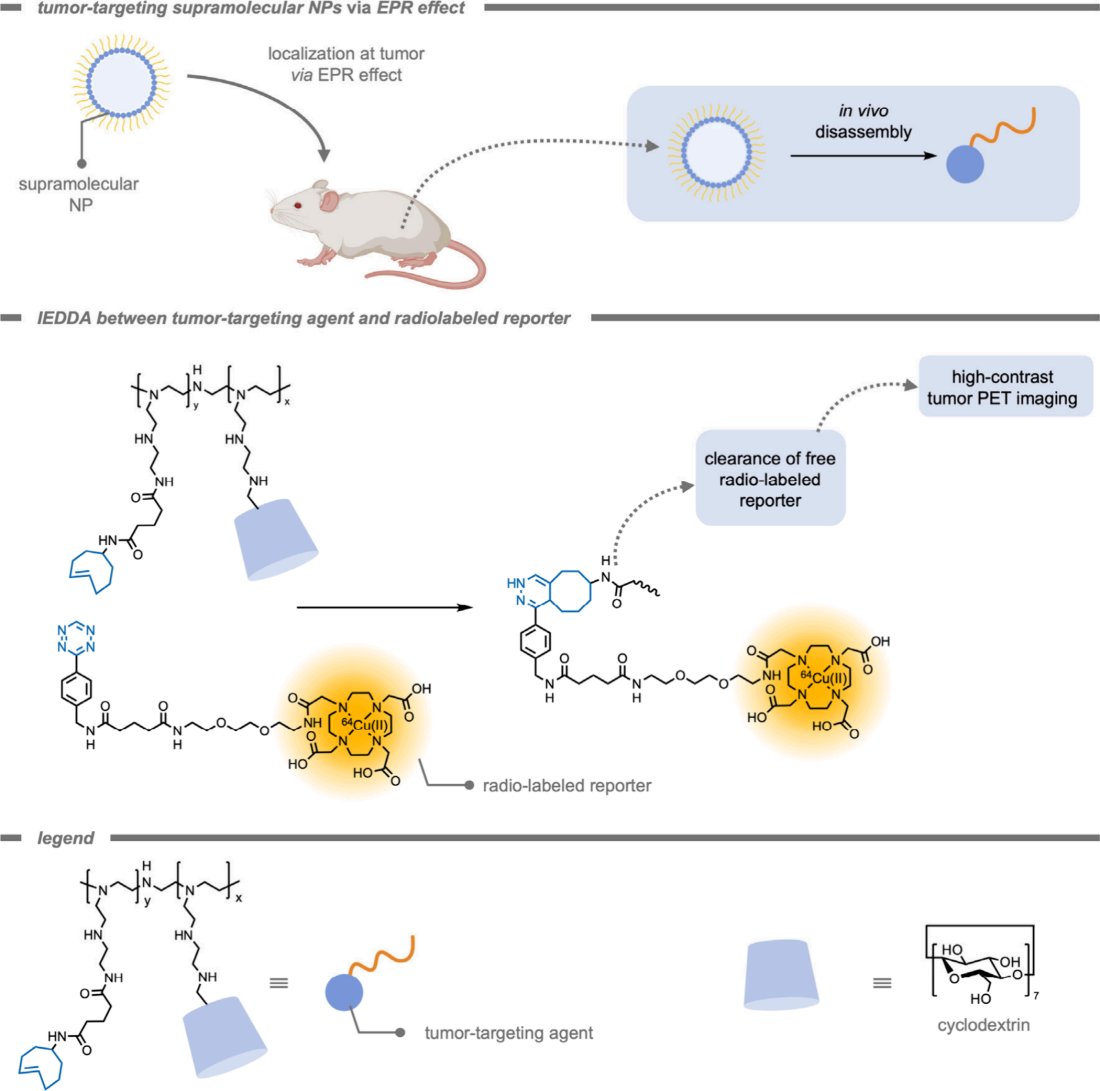
Supramolecular nanoparticles containing a trans-cyclooctene motif for an inverse-electron demand Diels–Alder reaction within cancer cells, resulting in improved positron emission tomography resolution^214^

In 2017, Du, Wang, and colleagues^215^ developed an immunostimulatory polymersome that could deliver two different cargos each instructed to their own corresponding target cells (Figure 22). The authors developed tumor microenvironment-sensitive cluster NPs to load both drug BLZ-945 and a Pt-based prodrug. The hydrophobic BLZ-945 molecule was encapsulated in the hydrophobic domain of the NP, while the Pt-based prodrug was covalently conjugated to the NP. The pH-sensitive (pH < 6.8) NPs rapidly disassembled into smaller particles upon exposure to the acidic tumor microenvironment.^216^ The authors proposed that this property could lead to rapid release of BLZ-945 in the perivascular regions, where it would be preferentially taken up by tumor-associated macrophages. The Pt-based prodrug-conjugated small particles would penetrate deeper into the tumor tissues to release the active Pt drug intracellularly by reduction of the Pt­(IV) to the Pt­(II) cisplatin species. The authors demonstrated that this concurrent delivery system improved tumor growth suppression (∼95% compared to ∼25.4% for clusters only loaded with BLZ-945), more effectively inhibited metastasis, and prolong the survival of tumor-bearing mice by 96.1% compared to the monotherapy controls.

**Figure 22:**
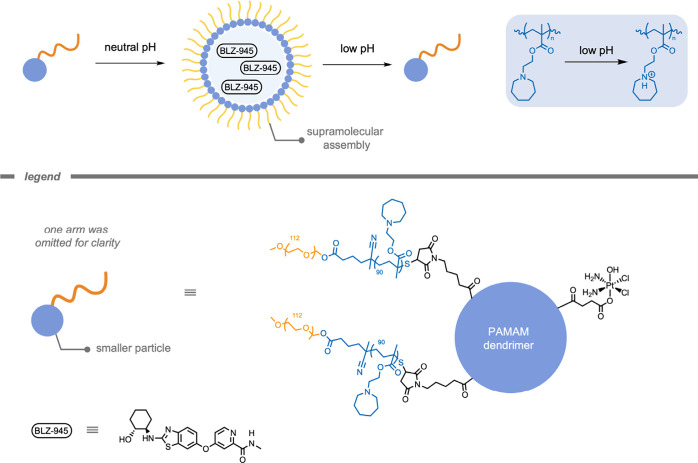
A pH-sensitive supramolecular polymersome system to spatially deliver two therapeutics to their own corresponding target cells.^215^

An alternative strategy to equip cells with catalysts for prodrug deprotection was demonstrated by Glorius, Jose, and co-workers (Figure 23).^217^ Based on previous work in which they reported cholesterol-based imidazolium salt lipid analogues that are incorporated into mammalian membranes,^218^ they integrated an artificial lipid mimetic NHC–Pd complex into a bacterial (*E. coli*) membrane that subsequently enabled the bacteria to perform propargyl ether deprotection reactions in the surrounding medium. Integration of the artificial lipid Pd complex seemed to rely on the cholesterol backbone, as NHC–Pd incubated cells (1 mM) did not show any depropargylation activity on a coumarin derivative in medium. Furthermore, the authors used the same TMC for a cascade reaction by combining it with an enzymatic laccase.

**Figure 23:**
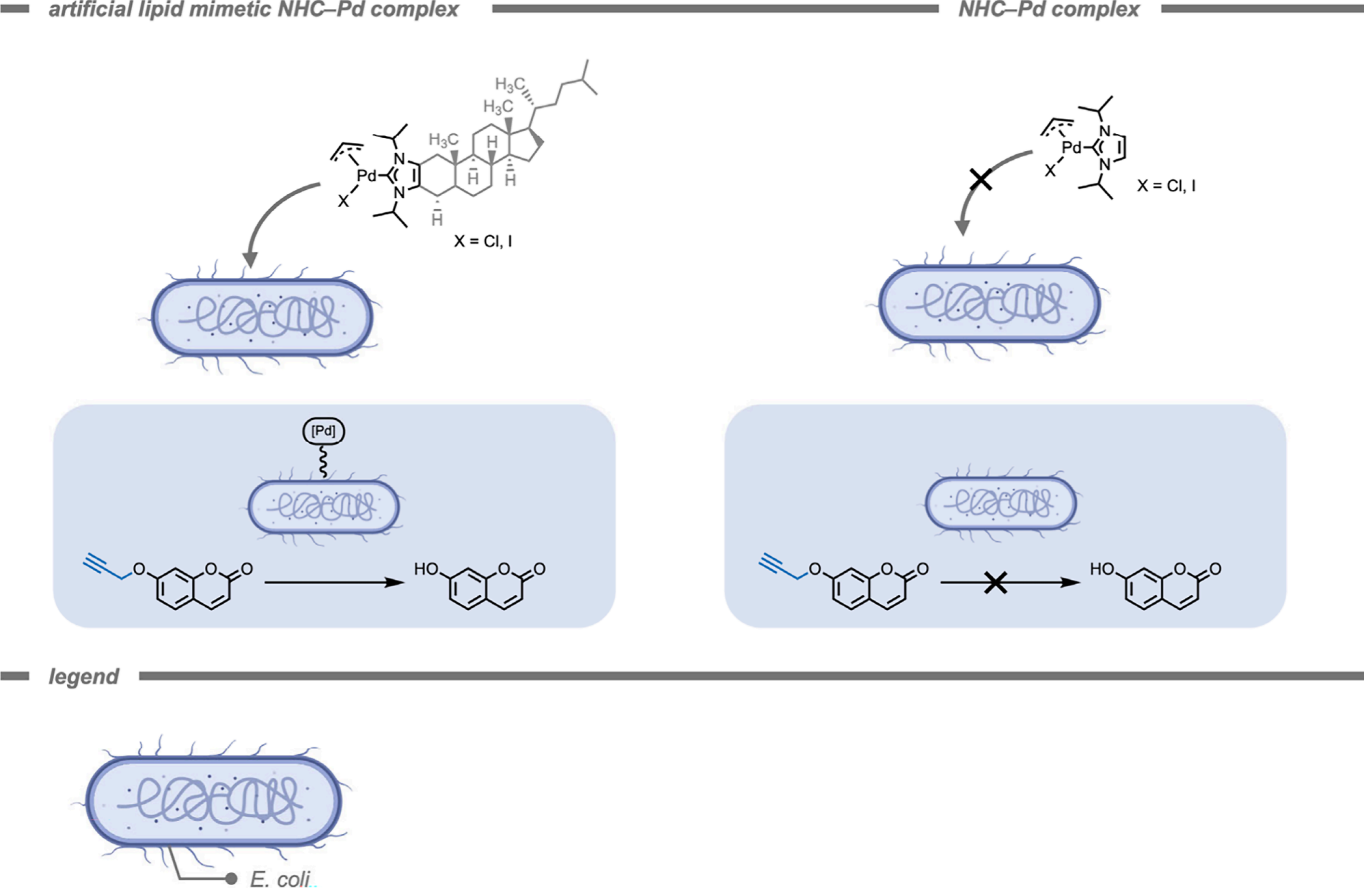
Incorporation of artificial lipid mimetic N-heterocyclic carbene (NHC)–Pd complexes into *E. coli* membranes led to successful propargyl ether deprotection in medium, whereas no deprotection was observed for NHC–Pd complexes due to lack of incorporation into the membranes.^217^

While this review was in submission, Dankers and co-workers^219^ reported the bioorthogonal release of a TCO-protected prodrug from a supramolecular polymer material by reaction with a tetrazine to reduce cell viability of breast cancer cells.

### 4.3 Enzyme-Instructed Self-Assembly

While numerous studies have reported the development of supramolecular carrier systems through synthetic procedures prior to administration, an alternative approach involves leveraging *in situ* supramolecular assembly within biological systems. For instance, enzyme-instructed self-assembly (EISA) has been utilized to selectively accumulate substrates for bioorthogonal reactions within cancer cells.^220^ During an EISA process, small, protected tripeptide building blocks undergo enzymatic deprotections, upon which they start to self-assemble. As this process is highly dependent on the activity and presence of specific enzymes, EISA can be employed to target abnormal enzyme activities commonly observed in tumor cells. For example, Chen, Gao, and co-workers^221^ employed an EISA supramolecular structure to accumulate the starting materials selectively within cancer cells prior to the bioorthogonal deprotection reaction (Figure 24). The authors targeted cancer cells that overexpressed phosphatase using a phosphorylated tetrazine-bearing tripeptide moiety. Over time, the authors found significant accumulation of the EISA-supramolecular structures containing tetrazine. After the cell culture was washed, a TCO-containing prodrug was administered, which was selectively activated in cancer cells through the IEDDA reaction and left intact in healthy cells. The authors stated that the selectivity of cancer cells over healthy cells was enhanced 10–20-fold using this strategy compared to administration of the drug itself.

**Figure 24:**
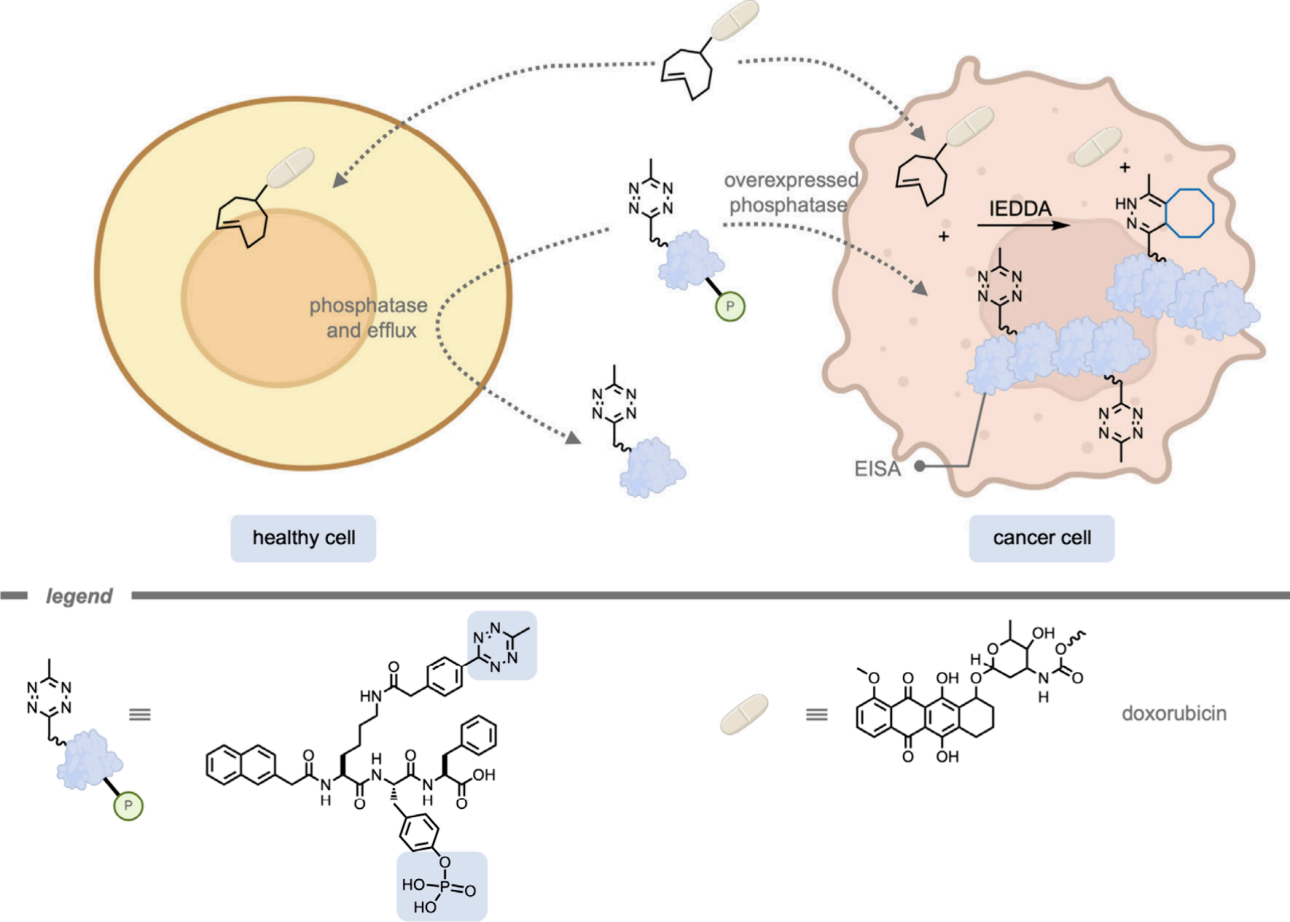
An enzyme-instructed self-assembled tetrazine-bearing structure selectively formed in cancer cells with upregulated phosphatase activity for trans-cyclooctene-protected prodrug activation.^221^

A similar example by Lin, Gao, and co-workers^222^ demonstrated the IEDDA activation of various prodrugs in cancer cells after initial targeting (Figure 25).^68^ This work also made use of the EISA-supramolecular assembly from phosphorylated tetrazine-bearing tripeptides specifically forming in cancer cells with upregulated phosphatase activity. Various TCO-protected drugs (e.g., doxorubicin, paclitaxel, monomethyl auristatin F methyl ester, and trichothecene mycotoxin) were deprotected using this strategy to demonstrate its general applicability. Through this supramolecular assembly containing tetrazine-bearing tripeptides, the authors demonstrated that a combination of TCO-prodrugs could be activated simultaneously, and switching of drug identities can help combat multidrug resistance.

**Figure 25:**
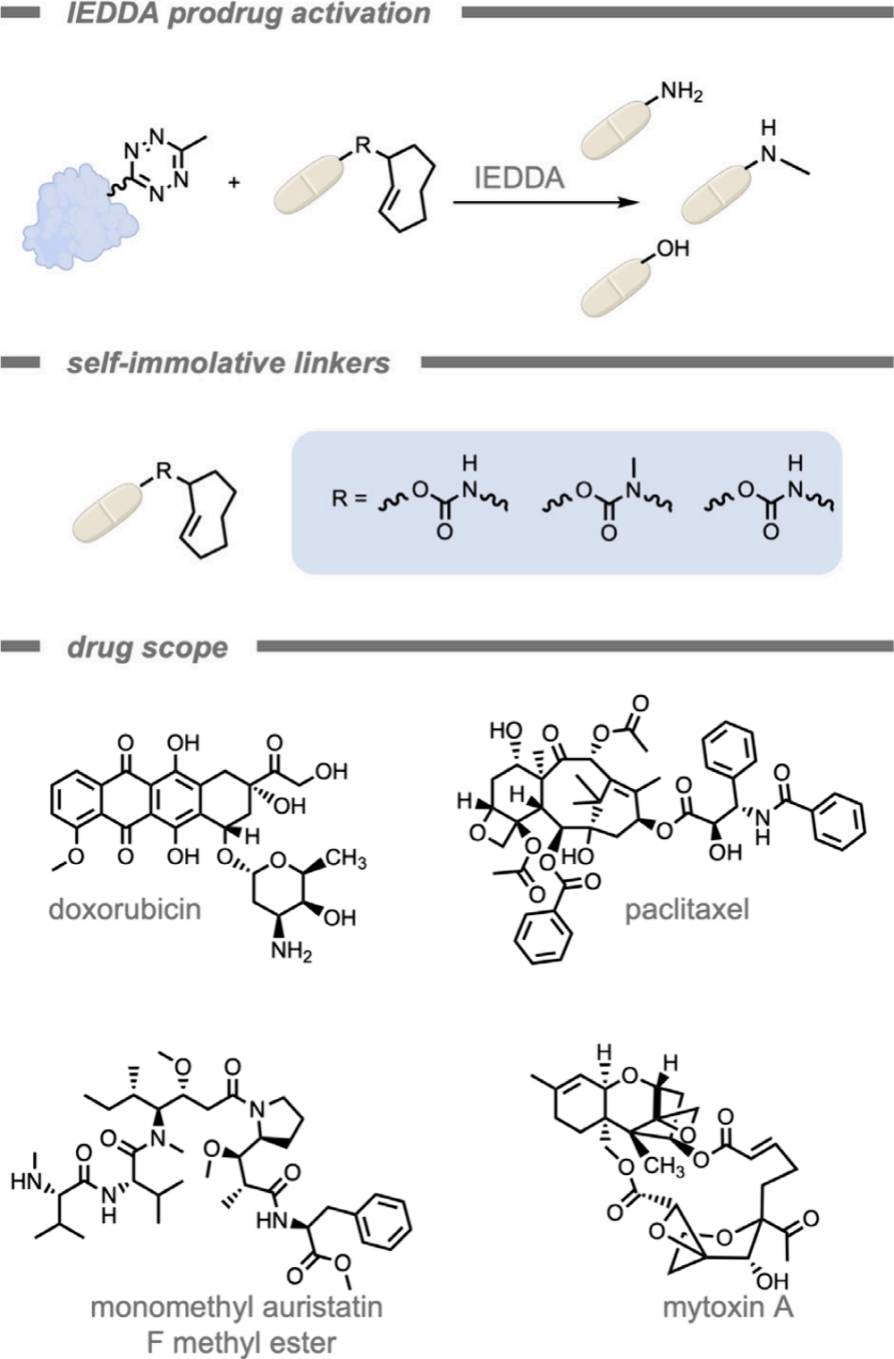
Bioorthogonal prodrug activation in cancer cells by a tetrazine-peptide enzyme-promoted supramolecular assembly.^222^

In 2023, Lo and co-workers^223^ reported the use of an EISA-approach to target intracellular or extracellular regions of the cell (Figure 26). They used ruthenium­(II) polypyridine complexes as reactive functionality. These Ru complexes can function as PSs for photodynamic therapy (PDT), show controllable near-infrared (NIR) phosphorescence, and possess triplet metal-to-ligand charge transfer emission with high emission sensitivity to the environment around the TMC.^224,225^ The Ru complexes were functionalized with a tetrazine group to react with a strained cyclooctene group on the self-assembled supramolecular structure within the cancer cell. The emission intensity and lifetime of the Ru complexes was significantly increased because of the accumulation of active groups on the supramolecular assembly. Part of the increase in emission was explained by the environmental sensitivity of the Ru complexes, yielding higher signals in the local hydrophobic microenvironment. Incubation of the complexes with the self-assembling unit as well as alkaline phosphatase to remove the phosphate group enhanced the emission intensity for the complexes from *I*/*I*_0_ = 0.7–1.0 to *I*/*I*_0_ = 12.4–109.9. The emission lifetime was increased to 0.38–0.66 μs. Moreover, changing the dosage of the self-assembling units changed the location of self-assembly, with higher dosages leading to self-assembly on the outside of the cell membrane. This subsequently led to an extracellular bioorthogonal reaction.

**Figure 26:**
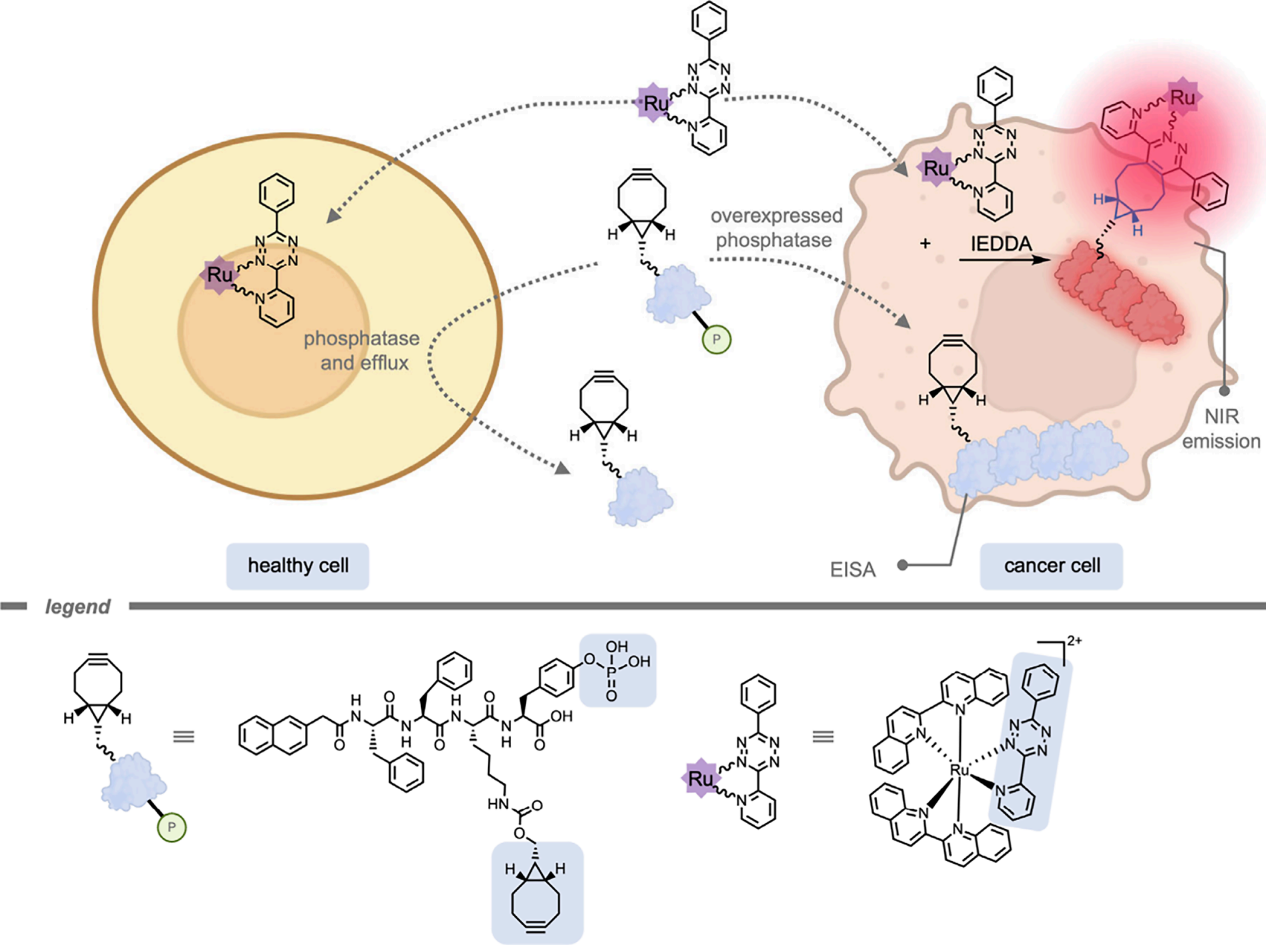
An enzyme-instructed self-assembly approach for targeted bioorthogonal reactions within cancer cells with upregulated phosphatase activity.^223^

While this review was in submission, Gao and co-workers^226^ reported the use of EISA containing tetrazine-handles on the cancer cell membrane to selectively deprotect TCO-protected melphalan and increase therapeutic efficacy toward cancer cells compared to tumor cells.

### 4.4 Reactive Oxygen Species-Induced Self-Assembly

Alongside the use of enzyme-instructed self-assemblies, other stimuli have been identified for selective targeting. For example, in 2022, Gao and co-workers^227^ demonstrated the use of ROS-induced tetrazine-bearing premonomers for selective targeting of ROS-overproducing tumor cells (Figure 27). The designed molecular precursors were composed of hydrogen peroxide (H_2_O_2_)-activatable fluorogenic BQA (a quinazolinone derivative capped with an aryl boronate group). Upon exposure to ROS, the BQA precursor was oxidized to restore the phenol, which subsequently formed an intramolecular H-bond. This intramolecular H-bond in turn led to a significant increase in planarization of the molecule. This high degree of planarization strongly enhanced π–π stacking between the precursor molecules, upon which they self-assembled into larger structures.^228^ The installed tetrazine moiety served a dual function: it acted as a fluorophore quencher, and it performed as a handle for the IEDDA reaction. In their design, an IEDDA reaction with a TCO-protected paclitaxel drug molecule allowed for the release of the active drug as well as a strong increase in fluorescent emission. Through this strategy, the emission intensity could be correlated to the administered dosage of active drug. The calculated therapeutic index for bioorthogonal activation was over 5.6-fold compared to free paclitaxel, since healthy cells were left intact as they did not accumulate tetrazine.

**Figure 27:**
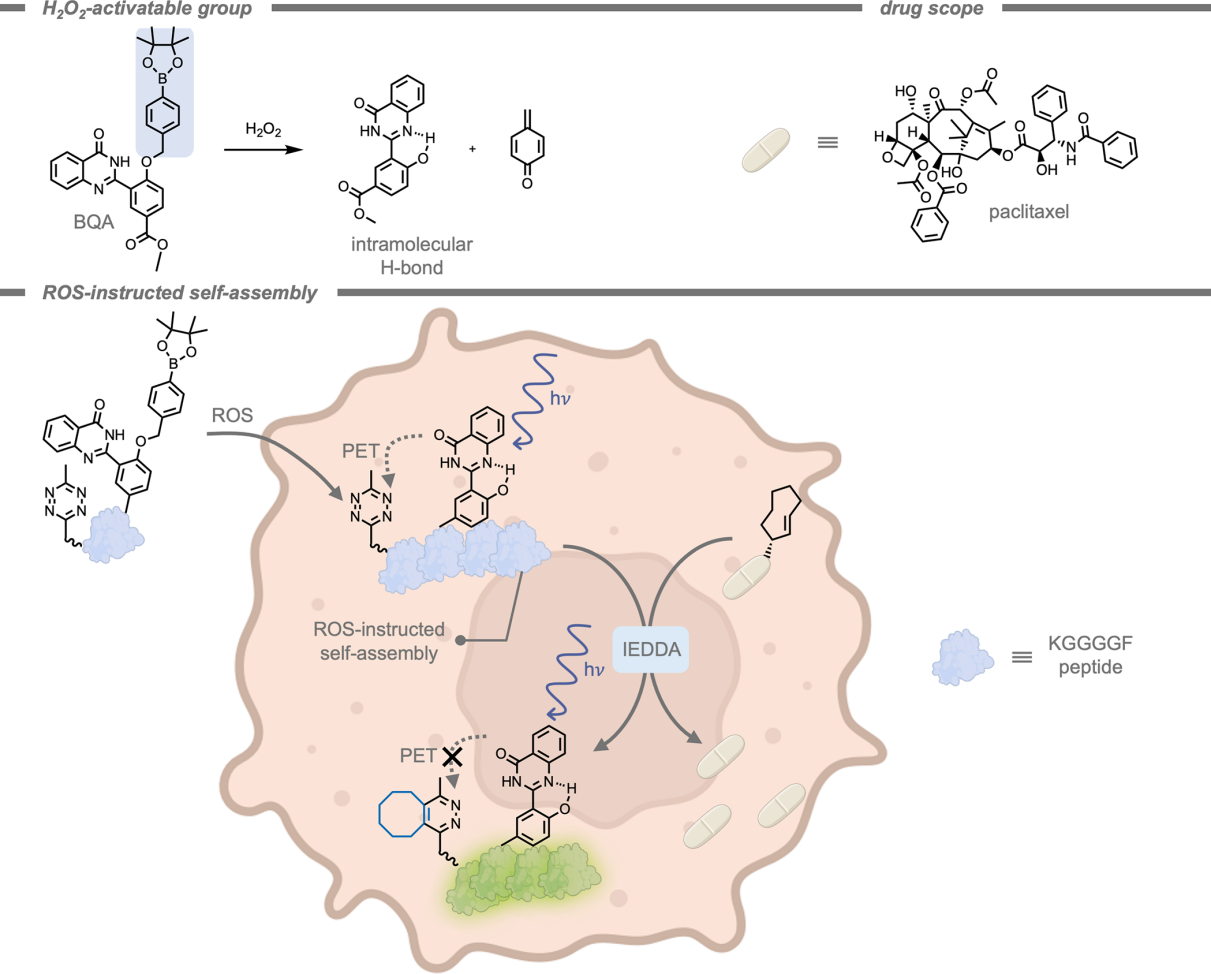
Reactive oxygen species-induced self-assembly for selective prodrug activation within cancer cells while simultaneously turning on fluorescent emission to monitor the reaction.^227^

Another stimulus-responsive self-assembly strategy for selective targeting in biological systems was reported by Cai, Gao, and co-workers^229^ in 2024. They demonstrated the use of ROS-induced self-assembly for the selective release of gamma-amino butyric acid (GABA)—a naturally occurring inhibitory neurotransmitter—inside mouse brains (Figure 28). In patients with epilepsy, evidence suggests neuro-inflammation and oxidative stress due to excessive presence of ROS. As the ROS level in epileptic lesions is increased, ROS-instructed self-assemblies formed under epileptic conditions. Tetrazine-bearing precursors were oxidized and subsequently self-assembled inside primary neurons and mouse brains. The local accumulation of tetrazine moieties at the site of interest allowed for targeted bioorthogonal release of GABA through two release mechanisms: (**i**) for the acute seizure model, the supramolecular assemblies directly enable local activation of GABA through bioorthogonal prodrug release. This leads to more direct inhibition of neuronal hyperexcitability than direct GABA supplementation. The GABA concentration in the hippocampus of kainic acid-induced acute seizure mice (∼608 μg g^–1^) was much lower than that of healthy animals (∼1105 μg g^–1^). By direct GABA supplementation, the GABA concentration was recovered to a certain extent (∼917 μg g^–1^), whereas treatment by deprotection of TCO-GABA could fully recover the GABA concentration (∼1116 μg g^–1^). Then, (**ii**) for the chronic seizure model, the authors stated that a bioorthogonal ligation reaction between tetrazine and transcyclooct-4-enol provided a reservoir of photoactivatable GABA. For chronic epileptic mice in the latent stage, the GABA concentration (∼94 μg g^–1^) was much lower than that of healthy mice. The treatment of TCO-linker-GABA prodrug and ultraviolet (UV) irradiation raised the hippocampal GABA concentration (∼961 μg g^–1^) but did not restore it fully. Only the ROS assembly assisted treatment with TCO-linker-GABA and UV irradiation fully restored the hippocampal GABA concentration (1105 μg g^–1^) to the normal value (∼1098 μg g^–1^). The GABA concentration results were consistent with the neurophysiological recordings of both acute and chronic epileptic mice.

**Figure 28:**
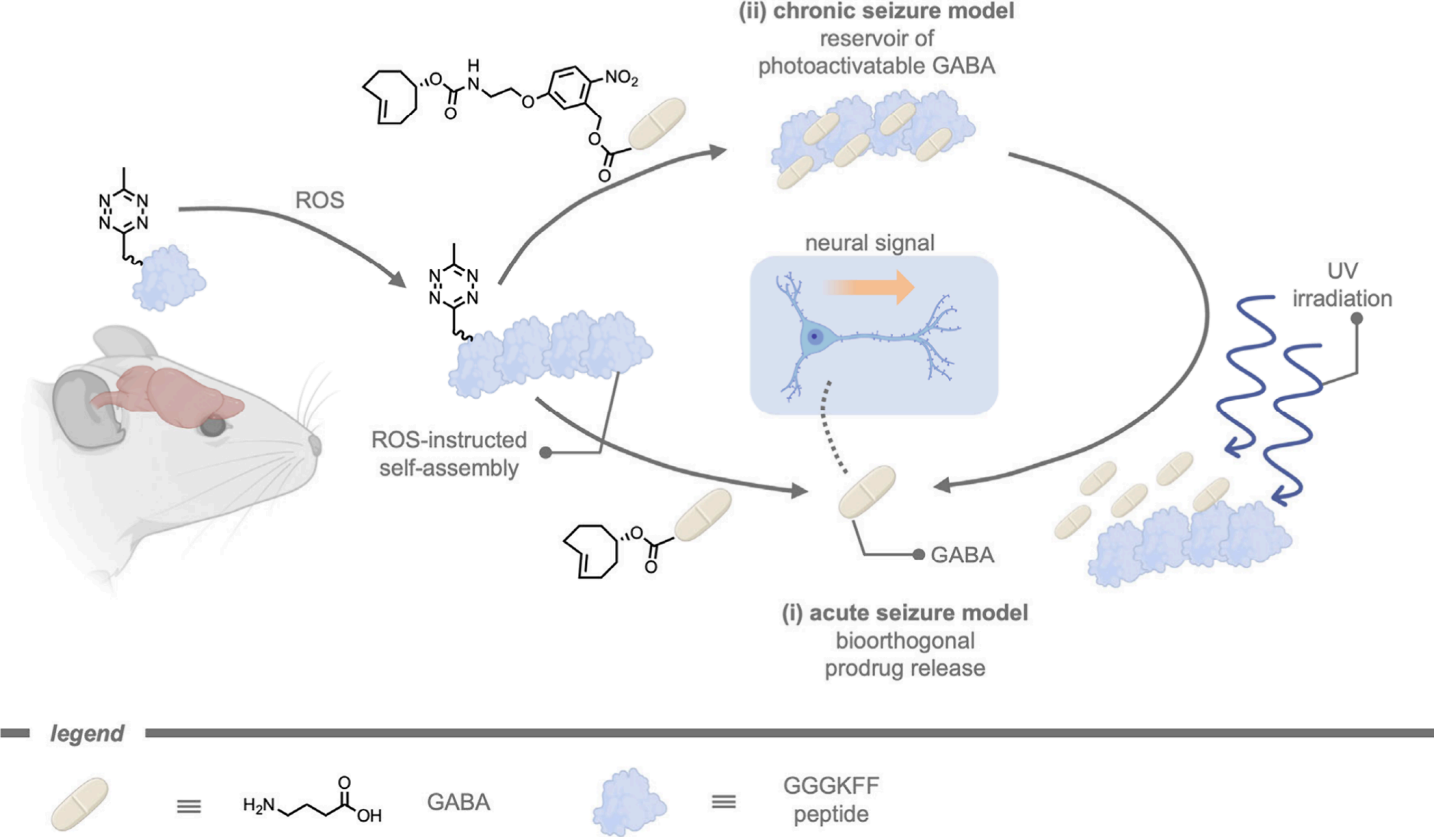
Reactive oxygen species-induced supramolecular self-assembly for bioorthogonal gamma-amino butyric acid release in mouse brains.^229^

While this review was in submission, Jiang, Gao, and co-workers^230^ reported the use of ROS-induced supramolecular assemblies for the bioorthogonal activation of a prodrug, enabling tumor-specific protein degradation in the context of pancreatic cancer.

### 4.5 Metal–Organic Frameworks and Hydrogen-Bonded Organic Frameworks

The combination of supramolecular substrate delivery systems with catalytically active moieties implemented in larger self-assembled structures has also been demonstrated, aiming for selective release and targeting. The tunable structure of MOFs allows for the integration of targeting moieties, such as phosphonium tails or antibodies.^93,231–234^ For example, Cu^I^-containing MOFs modified with antibodies for targeting of specific analytes were reported by Song, Wang, and co-workers^235^ for the application in ultrasensitive fluorescence counting immunoassays for immobilization of EGFP-tagged bacteria using the CuAAC reaction. The wide variety in possible compositions of MOF materials make them well-suited for applications in biological systems, as the composition has an influence on the stability,^236^ biocompatibility, and biodegradability of MOFs. For example, biocompatibility is influenced by the nature of the coordination metal and the organic linker, as well as the size, shape, and surface charge of the overall particle.^237^ For a comprehensive analysis on the biocompatibility and biodegradability of MOFs, the reader is referred to several reviews.^237–240^

Several MOFs with targeting ability have already been investigated for *in vitro* catalysis. For example, Qu and co-workers^241^ constructed a MOF functionalized with catalytic Cu^0^ centers and triphenylphosphonium tails for mitochondrial targeting (Figure 29). The primary function of the MOF was to provide a protecting scaffold for the nanoscale Cu particles. Subsequently, they performed *in vitro* drug synthesis of resveratrol through the CuAAC reaction from two precursor molecules that showed negligible cytotoxicity at concentrations lower than 25 μM in MCF-7 cells. As a result of the synthesis of resveratrol in mitochondria, cell viability was considerably decreased and mitochondrial damage, accumulation of oxidative stress, and release of cytochrome *c* were observed. Interestingly, direct incubation of resveratrol to MCF-7 cells resulted in a survival rate >80% and no obvious increase in oxidative stress, even at the highest tested concentration (50 μM). These results indicate that *in vitro* drug synthesis in the organelle of interest can significantly improve the efficacy and reduce side effects.

**Figure 29:**
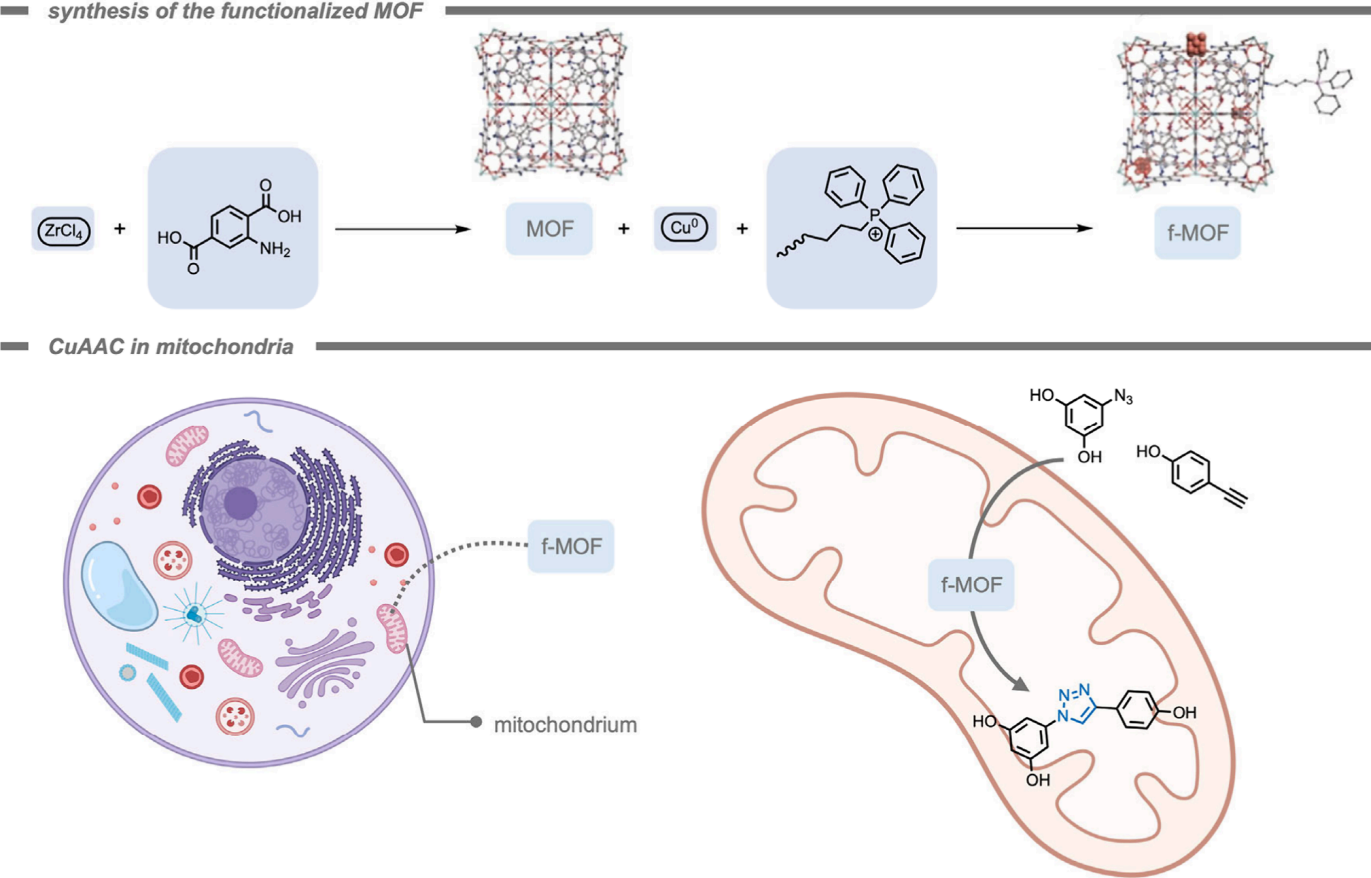
A Cu-functionalized metal–organic framework containing triphenylphosphonium tails for mitochondrial targeting and subsequent drug synthesis on location.^241^ Reproduced in part with permission from ref 241. Copyright 2019, Wiley Publishing.

Later, the same group reported a bimetallic MOF encapsulating a DNAzyme—a DNA oligonucleotide that is capable of facilitating a specific chemical reaction—for combined *in vitro* drug synthesis and gene therapy (Figure 30).^242^ The authors employed a ZIF-8 as a delivery vehicle, which degrades at the low pH of cancer cells, and subsequently releases copper ions, zinc ions, and DNAzyme. The DNAzyme functioned as a gene silencing tool by cleaving the EGR-1 mRNA. The MOF NPs acted as a protective scaffold and supplied Zn^2+^ as a cofactor for the catalytic cleavage activity of the DNAzyme. Cu catalyzed the CuAAC reaction to form a resveratrol derivative. The generated resveratrol product can kill cancer cells, and the DNAzyme activated by Zn^2+^ strongly inhibited the proliferation and metastasis of cancer cells. In the orthotopic-transplant metastatic model of nude mice, the DNAzyme@Cu/ZIF-8-dosed group exhibited the strongest tumor growth inhibition (∼0.1 g), significantly outperforming the control groups: ∼2.5 g for PBS, ∼1.9 g for DNAzyme@ZIF-8, and ∼0.7 g for complementary DNA (cDNA)@Cu/ZIF-8. This combined therapy could avoid the off-target effects of traditional chemotherapy as well as inhibit tumor metastasis by cleaving the oncogene substrate.

**Figure 30:**
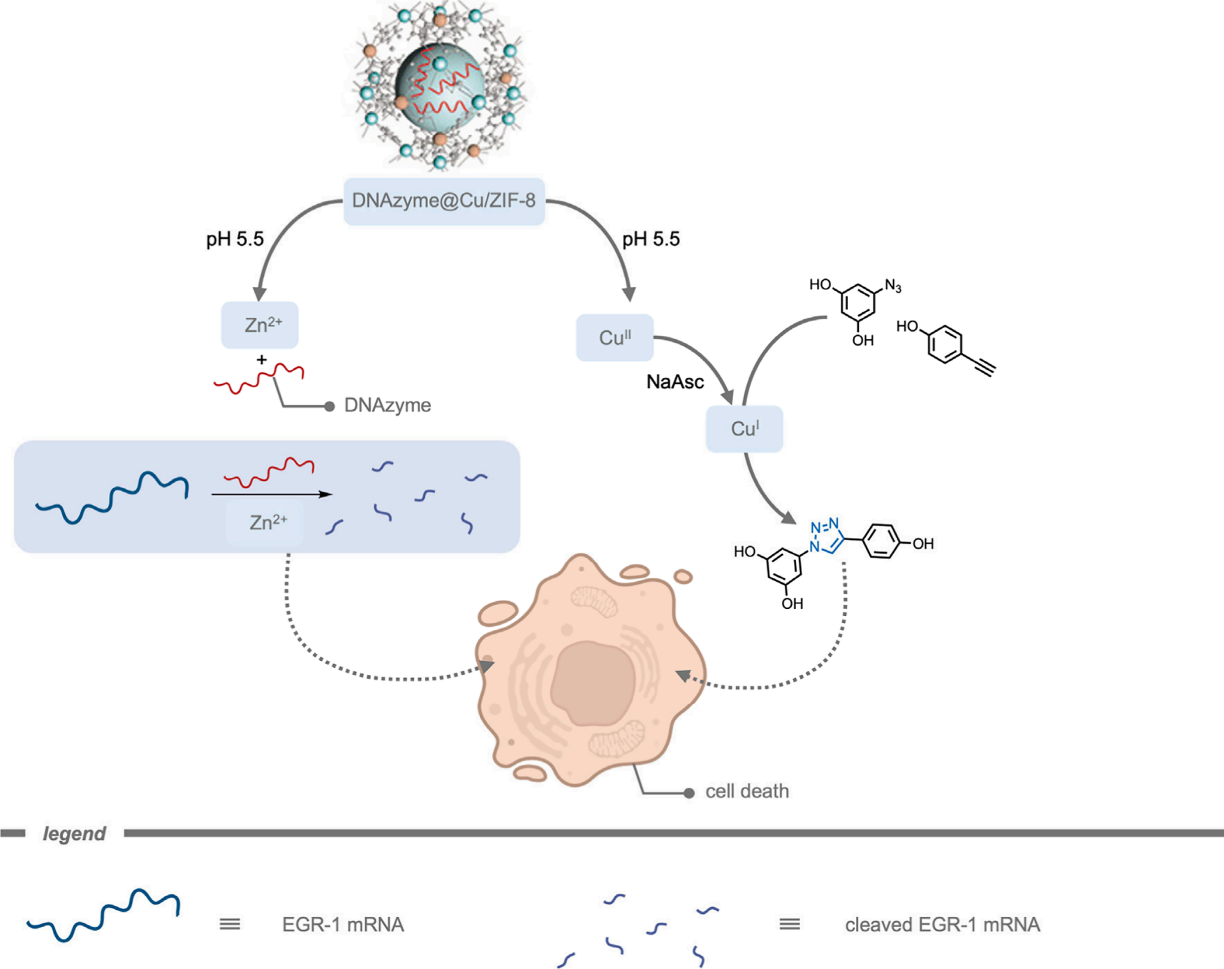
A bimetallic, acid-labile ZIF-8 metal–organic framework for *in vitro* drug synthesis and gene therapy in cancer cells.^242^ Reproduced in part with permission from ref 242. Copyright 2021, Wiley Publishing.

Inspired by this work, Liu and co-workers^243^ demonstrated PS synthesis in the mitochondrium (Figure 31). The authors developed a mitochondria-targeting PS with aggregation-induced emission properties (TPATrzPy-3+). This PS can be synthesized from two nonfluorescent precursors (TPA-alkyne-2+, and MePy-N_3_) through the CuAAC reaction. A Cu^II^-based MOF-199 was selected as an inert carrier for the PS precursors and as a Cu^I^ catalyst source to form PSs selectively in the cancer cells. After loading with the precursor molecules, the MOF-199 was coated with F-127 (a PEG-based nonionic detergent) to form PMOF NPs. The precursors showed very minor cytotoxicity toward both HeLa and 3T3 cells, while TPATrzPy-3+ showed significant ablation of both cell lines with an IC_50_ of ∼10 μM, similar to the ablation effect for HeLa cells by the PMOF NPs. Upon light irradiation, the mitochondria-targeting PS emitted red fluorescence and generated singlet oxygen (^1^O_2_) for cancer cell ablation. Under light, the PMOF NPs could selectively kill cancerous HeLa cells over 3T3 cells. Moreover, the authors demonstrated that in HeLa cells pretreated with GSH-scavenging buthionine sulphoximine, the IC_50_ increased over 50 μM, indicating that the target cancer cell ablation potency of the PMOF NPs under light was mediated by upregulated GSH levels. The upregulated GSH levels facilitated the *in situ* generation of TPATrzPy-3+. Further studies in tumor-bearing zebrafish models demonstrated that liver tumors of larvae treated by PMOF NPs shrank on average ∼40% after PDT (similar to treatment with TPATrzPy-3+ and TPATRzPy-3+@MOF NPs). In contrast, in larvae treated with precursors, illuminated only, or without any NP treatment, tumors grew by 40–100% within 2 days.

**Figure 31:**
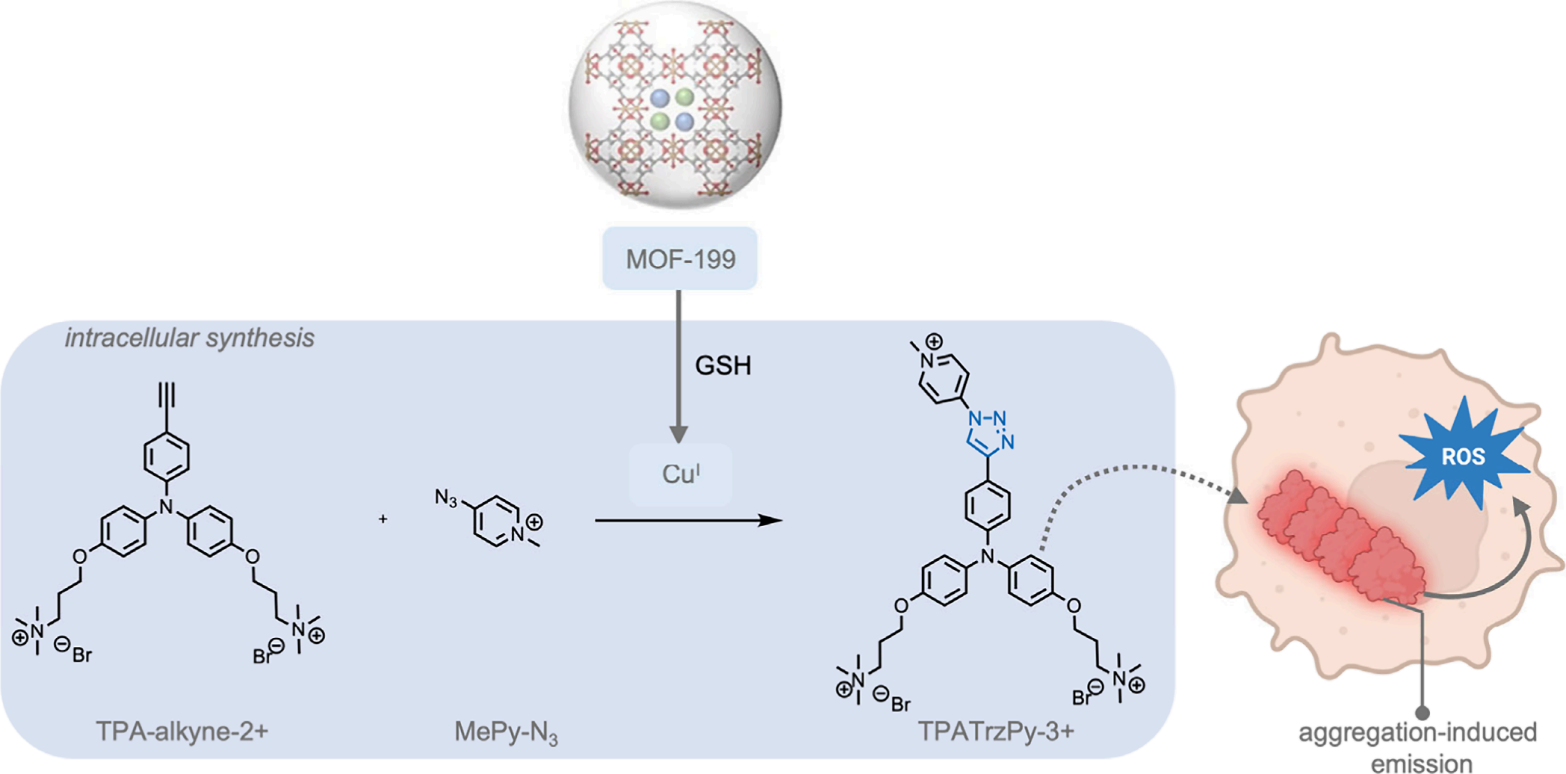
Cancer cell-induced photosensitizer synthesis in the mitochondrium through Cu^I^-catalyzed [3+2] azide–alkyne cycloaddition.^243^ Reproduced in part with permission from ref 243. Copyright 2021, Wiley Publishing.

To tackle the dosage issues generally observed with individual administration of a prodrug and a catalyst, Ren, Qu, and co-workers^244^ designed an “all-in-one bioorthogonal system” (Figure 32). The authors used Matérial Institut Lavoisier-101 (MIL-101­[Fe]) as protective scaffold for Pd NPs and then coated the MOF scaffold with a calcium carbonate (CaCO_3_) shell. On top of this shell, prodrug pro-5-fluorouracil (5FU) was incorporated as a thin layer using hyaluronic acid (HA) as the supporting polymer. In this system, the prodrug and catalyst are separated from each other by the CaCO_3_ shell to avoid premature drug release. Upon exposure to the acidic (pH ∼ 6.5) tumor environment, the CaCO_3_ shell collapsed. As a result, the prodrug was exposed to the bioorthogonal catalyst and activated specifically within cancer cells. According to the authors, this simultaneous release strategy decomplicates drug delivery to cancer cells using intravenous administration. The catalytic activity was first demonstrated for the deprotection of an allyl carbamate-protected coumarin dye in HeLa cells. The mean fluorescence intensity of the HA@CaCO_3_@MIL-101­(Fe)Pd (∼370) was significantly higher than that of the control groups (∼150). Further studies in U14 (mouse cervical cancer) tumor-bearing mice demonstrated that the pro-5FU-loaded HA@CaCO_3_@MIL-101­(Fe)­Pd platform significantly suppressed tumor growth (∼0.3 g) within 14 days compared to the control groups: ∼2.5 g for PBS, ∼2.3 g for prodrug only, and ∼1.3 g for HA@CaCO_3_@MIL-101­(Fe)­Pd without prodrug. The drug distributed in the tumor site was much higher than that in the major organs, underlining the efficacy of acidic tumor microenvironment-induced prodrug deprotection.

**Figure 32:**
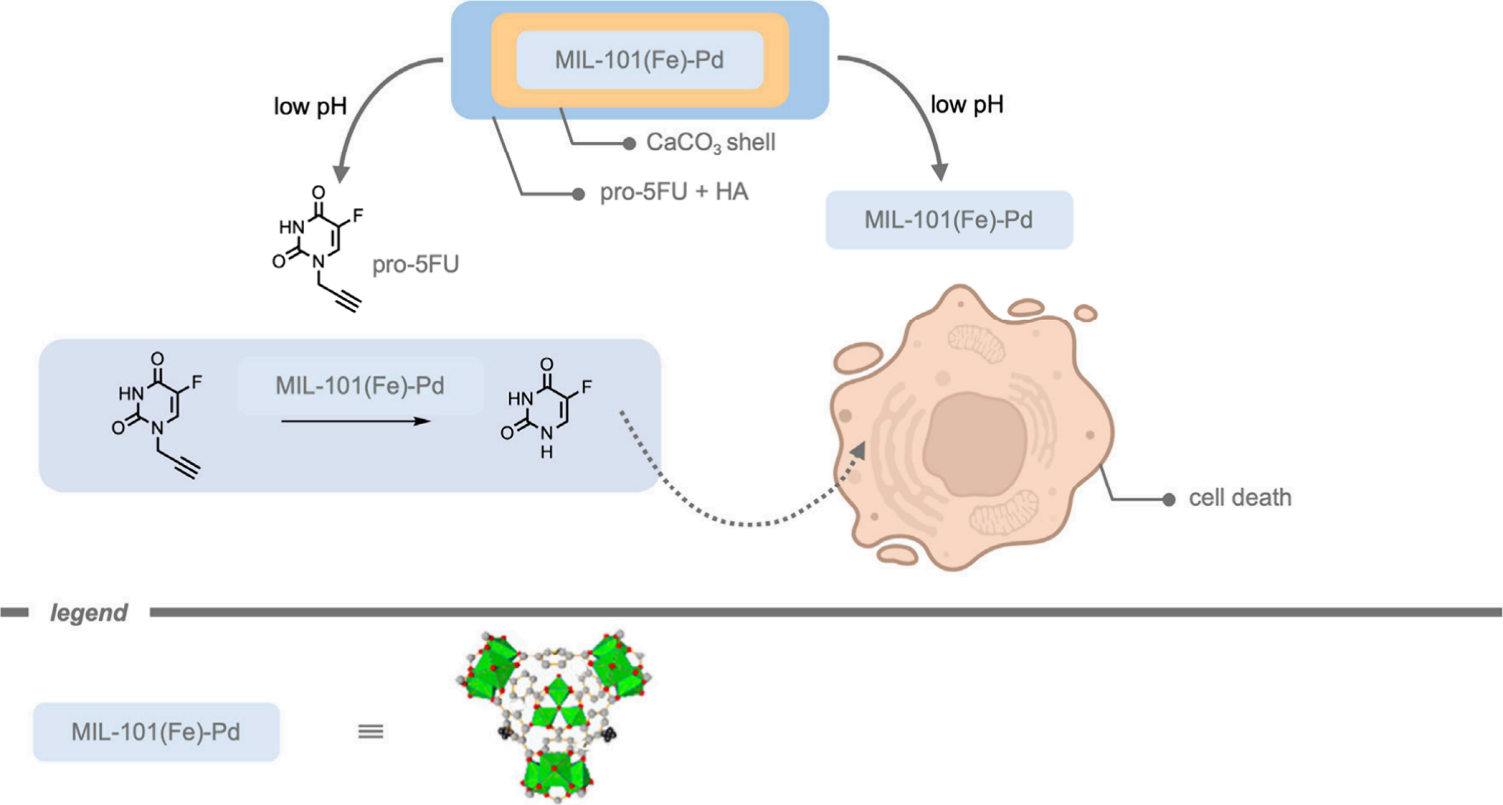
An all-in-one bioorthogonal platform consisting of a catalytically active metal–organic framework separated from the prodrug by a CaCO_3_ layer.^244^ Reproduced in part with permission from ref 244. Copyright 2022, ACS Publications.

Elaborating further on these MOF-based platforms, Qu and co-workers^245^ developed a cancer cell-selective membrane-cloaked NP MOF for prodrug activation and chemodynamic therapy (Figure 33). In chemodynamic therapy, toxic hydroxyl radicals (•OH) are generated *in situ* to kill tumor cells.^246–248^ The membrane-cloaked NPs contained biomimetic receptors and antigens, and therefore mimicked cancer cell behavior for active targeting and prolonged circulation. When MIL-53@F^–^ was cloaked with MCF-7 (human breast carcinoma) cell membrane vesicles, the uptake efficiency of the particles by MCF-7 cells was much higher than by 293T human embryonic kidney cells. The designed MIL-53 MOF, with high fluoride (F^–^) loading and pH-responsive degradation, releases F^–^ and Fe^3+^ in acidic environments. The authors demonstrated that F^–^ from MIL-53@F^–^@M triggered desilylation reactions in living cells, while Fe^3+^ generated •OH from H_2_O_2_, inducing oxidative stress and cell death. The 10-hydroxycamptothecin anticancer drug also activated nicotinamide adenine dinucleotide phosphate (NADPH) oxidase, replenishing H_2_O_2_ and enhancing therapy in living cells. Further studies in Heptatoma 22 (H22) tumor-bearing mice demonstrated that pro-camptothecin-loaded MIL-53@F^–^@H (H22 membrane-coated MOF) significantly inhibited tumor growth (∼100 mm^3^) in 14 days compared to the pro-camptothecin-loaded MIL-53@F^–^ (∼400 mm^3^), the empty MIL-53@F^–^@H (∼500 mm^3^), and the control (∼800 mm^3^), underlining the synergistic effect of the MOF platform.

**Figure 33:**
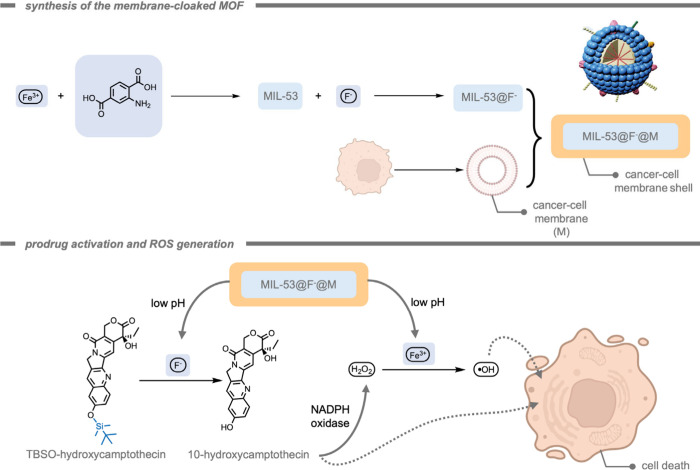
Cancer-cell selective membrane-cloaked metal–organic framework nanoparticles for prodrug activation and chemodynamic therapy.^245^ Reproduced in part with permission from ref 245. Copyright 2022, ACS Publications.

MOF materials offer easy surface chemistry engineering with the possibility of building block design for molecular recognition allowing targeted delivery. In 2022, Wang and colleagues^249^ developed an aptamer-engineered University of Oslo-66 (UiO-66) MOF with Pd NPs for bioorthogonal catalysis (Figure 34). The AS1411 aptamer binds to nucleolin on HeLa cancer cells, enhancing both cellular uptake and catalytic efficiency. The system successfully catalyzed depropargylation reactions for both activation of propargyl carbamate-protected rhodamine 110 in aqueous solution and living cells, as well as deprotection of propargyl-protected 4-hydroxytamoxifen. Deprotected 4-hydroxytamoxifen acts as a high-affinity ligand for the protein-stabilizing domain estrogen receptor 50 (ER50), which regulates protein stability and activity in cancer cells. By stabilizing ER50-fused proteins, their 4-hydroxytamoxifen-activating MOF can control the activity of bacterial effector proteins to inhibit the mitogen-activated protein kinase (MAPK) signaling pathway, offering a potential approach for cancer treatment. The authors fused the bacterial effector protein Osp-F—which dephosphorylates extracellular signal-regulated kinase 1/2 to inactivate MAPK signaling—with the destabilizing domain ER50 to suppress its activity. The authors demonstrated that Osp-F activity was restored because of the bioorthogonal activation of 4-hydroxytamoxifen.

**Figure 34:**
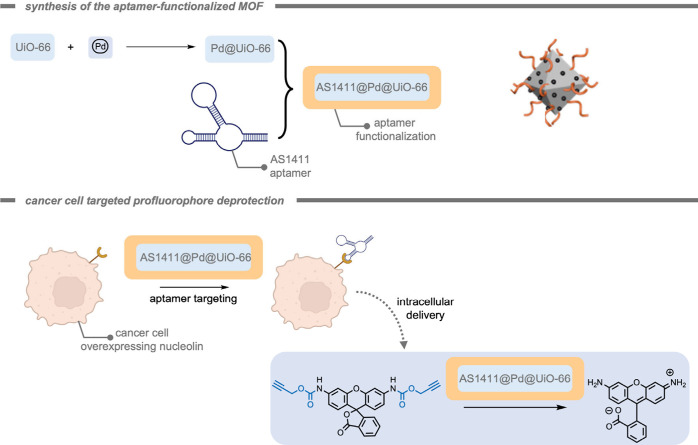
Aptamer-functionalized UiO-66 metal–organic framework containing Pd nanoparticles for intracellular bioorthogonal prodye activation.^249^ Reproduced in part with permission from ref 249. Copyright 2022, ACS Publications.

Qu and co-workers^250^ have also demonstrated the applicability of MOF systems for antitumor immunotherapy through bioorthogonal activation of Toll-like receptor 7 (TLR7) agonists and aptamers to block programmed-death ligand 1. This is an important signaling pathway between tumor cells and T cells (Figure 35). The MOF that was prepared contained catalytic Pd particles for the local deprotection of allyl carbamate-protected imiquimod. This drug has been shown to repolarize macrophages from the M2 to the M1 phenotype, thereby reprogramming the immunosuppressive tumor microenvironment. The aptamers not only function for cancer cell targeting, but also directly contribute to antitumor immunotherapy. They are dissociated from the MOF in the tumor microenvironment due to competitive binding of phosphate ions to the aptamers. Subsequently, these aptamers block the programmed-death ligand 1 checkpoint. By disrupting the interaction between the tumor cells and the T cells, these aptamers can reduce the immunosuppressive tumor microenvironment and help promote T cell activity against the cancer cells. Promising effects on the TLR7 activity were observed during *in vitro* studies on RAW264.7 macrophages, and further *in vivo* antitumor studies on single subcutaneous CT26 (colon carcinoma) tumor mice models indicated improved therapeutic efficiency of the supramolecular platform. After the primary tumor reached a size of 150 mm^3^, tumor treatment was administered every 2 days with intratumoral injection of the aptamer-Pd-MOF and intravenous injection for pro-imiquimod. Administration of aptamer-Pd-MOF significantly suppressed primary tumor growth within 14 days (∼150 mm^3^) compared to the control groups: ∼1100 mm^3^ for PBS, and ∼850 mm^3^ for aptamer-Pd-MOF. Although the effect of pro-imiquimod with Pd-MOF (without aptamer coating) was similar on the primary tumor growth (∼150 mm^3^), it was significantly less effective than the aptamer-coated MOF at suppressing distant tumor growth (∼200 mm^3^ compared to ∼50 mm^3^, respectively).

**Figure 35:**
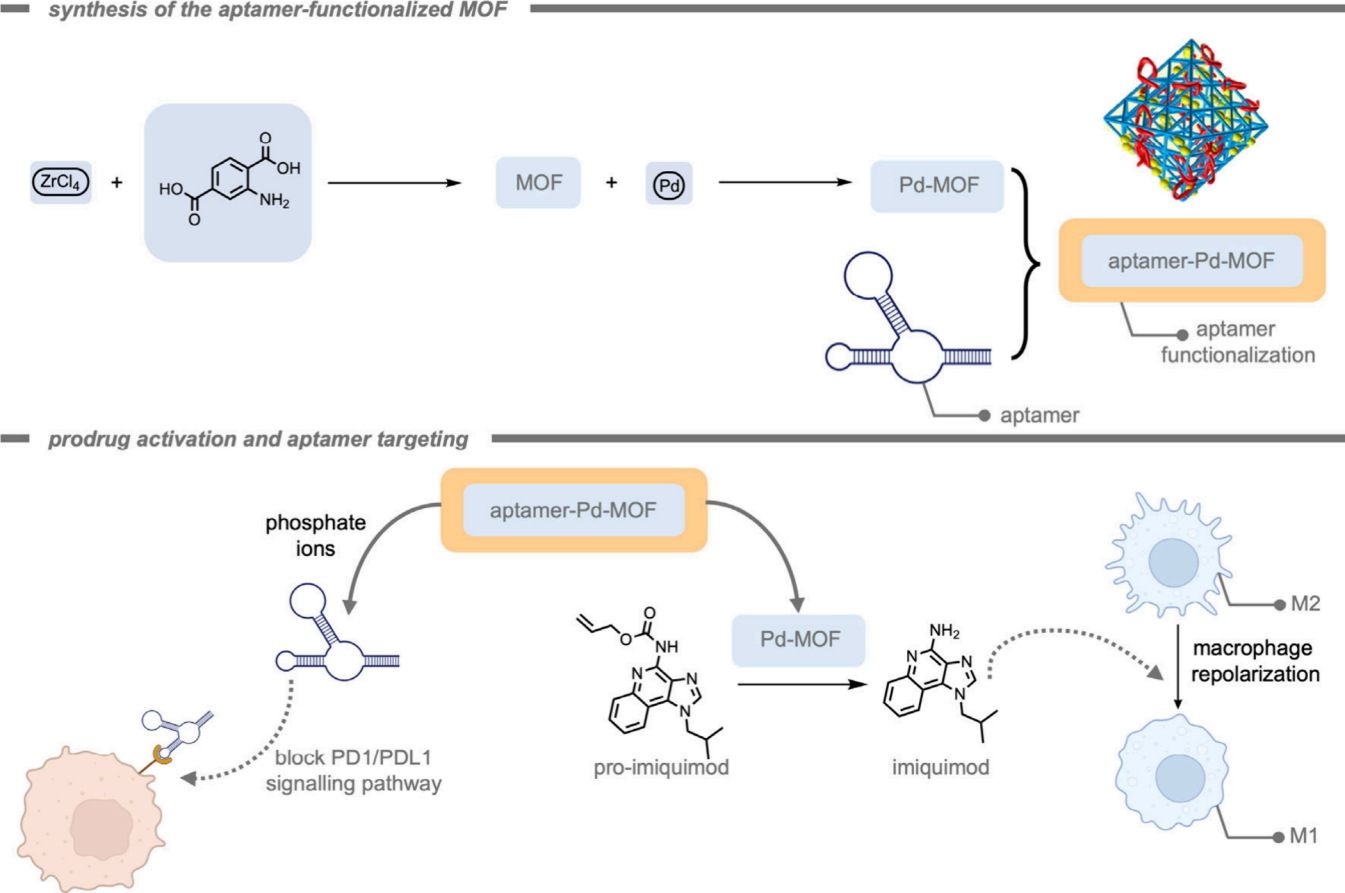
An aptamer-functionalized metal–organic framework containing catalytic Pd particles for *in vivo* prodrug activation and enhanced antitumor immunotherapy.^250^ Reproduced in part with permission from ref 250. Copyright 2023, ACS Publications.

Hydrogen-bonded organic frameworks (HOFs) are another class of supramolecular nanomaterials with high structural flexibility, biocompatibility, and porosity. These structures form through hydrogen bonding between well-designed building blocks.^251–253^ In 2023, Wang and co-workers^254^ applied these properties to design a nanoscale HOF containing a bioorthogonal catalytic center and an enzyme to enable dual catalysis within mitochondria (Figure 36). The primary aim of this platform was to generate hydrogen sulfide (H_2_S) in mitochondria, since H_2_S is a gaseous signaling molecule partaking in mitochondrial biogenesis and cellular protection against oxidative stress. A pro-H_2_S compound was protected with an aryl azide carbonate protecting group, which was catalytically photoreduced by Ru catalysts. The subsequently formed carbonyl sulfide was rapidly converted to H_2_S by cellular carbonic anhydrases. The authors obtained their HOF through self-assembly of tris­(4,4′-dicarboxylicacid-2,2′-bipyridyl) ruthenium­(II) as catalytically active compound with [1,1′-biphenyl]-4,4’′-biscarboximidamide. The obtained HOF is based on charge-assisted hydrogen bonds. Preparation of the HOF in the presence of proteins led to HOF-encapsulated protein materials, allowing for synergistic (i) bioorthogonal Ru-mediated photocatalysis and (ii) enzymatic catalysis. Moreover, these protein-loaded HOFs were selectively accumulated within mitochondria, which the authors attributed to the favorable electrostatic interaction between the cationic amidinium moiety within the HOF and the negatively charged inner membrane of the mitochondria. The cytoprotective effect of the HOF was demonstrated by incubation of SH-SY5Y (neuroblastoma) cells with 6-hydroxydopamine hydrobromide, a neurotoxin known to induce cellular oxidative stress and neurogenerative disease. This resulted in a decrease of cell viability to ∼40% compared to the control. Only pretreatment by coadministration of the catalase@HOF with pro-H_2_S and light irradiation led to a significant increase of cell viability to ∼72% compared to other controls: ∼40% for HOF+pro-H_2_S, and ∼40% for catalase@HOF. This increase demonstrates the potential for dual action of catalase scavenging ROS and the *in vitro* generation of H_2_S by this supramolecular platform. The overall stability of supramolecular structures based on hydrogen bonds (i.e., HOFs) in the highly competitive cellular environment is still largely unexplored. In this work, the Ru-containing HOFs possessed colloidal stability in deionized water up to 4 h, and the authors confirmed that the morphology and structural integrity of the HOFs was intact after pretreatment with water, cell medium, and sodium acetate buffer (pH 4.8 and 3.9) for 24 h. Moreover, model studies on protein release from GFP@HOFs under acidic or cell medium conditions showed <3% GFP release. Lastly, the authors demonstrated the enhanced mitochondrial uptake of the Ru-containing HOF compared to administration of the unassembled Ru organic linker, providing some insight into the stability of the HOF in biological environments.

**Figure 36:**
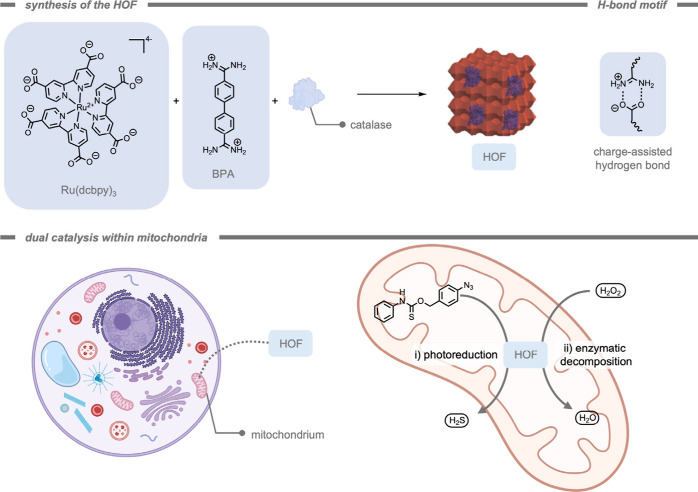
Synergistic bioorthogonal Ru-mediated photocatalysis and enzymatic catalysis by a functionalized hydrogen-bonded organic framework in mitochondria.^254^ Reproduced in part with permission from ref 254. Copyright 2023, Wiley Publishing.

Another dual action HOF-based platform was described by Qu and co-workers,^255^ who demonstrated the use of a HOF as both prodrug delivery system and catalyst to convert the prodrug into the active drug (Figure 37). Their HOF consisted of ferric porphyrin building blocks, which were reduced to the catalytically active ferrous porphyrin units in the presence of upregulated GSH concentrations commonly found in tumor microenvironments. These ferrous porphyrins can deprotect the aryl azide-protected 5FU, releasing the active anticancer drug in the presence of GSH. Under biorelevant conditions, the conversion increased 70-fold when GSH was added to the reaction mixture, indicating that GSH is crucial for drug deprotection. With GSH concentrations 10-fold higher in tumor cells than in macrophages, GSH-dependent activation led to successful catalysis in tumor cells but showed no significant activity in macrophages. Impressively, the authors demonstrated the dual release and cleavage of secondary aryl azide carbamate-protected drug 5-ethynyluracil. This therapeutic inhibits metabolic deactivation by dihydropyrimidine dehydrogenase (DPD) of 5FU, improving the clinical efficacy of 5FU. Studies in orthotopic metastatic models of nude mice demonstrated that tumor growth was significantly inhibited in the group injected with both prodrug-containing aptamer@HOF (∼0 g) compared to the controls: ∼0.9 g for PBS, ∼0.7 g for aptamer@HOF, and ∼0.2 g for 5FU-containing aptamer@HOF.

**Figure 37:**
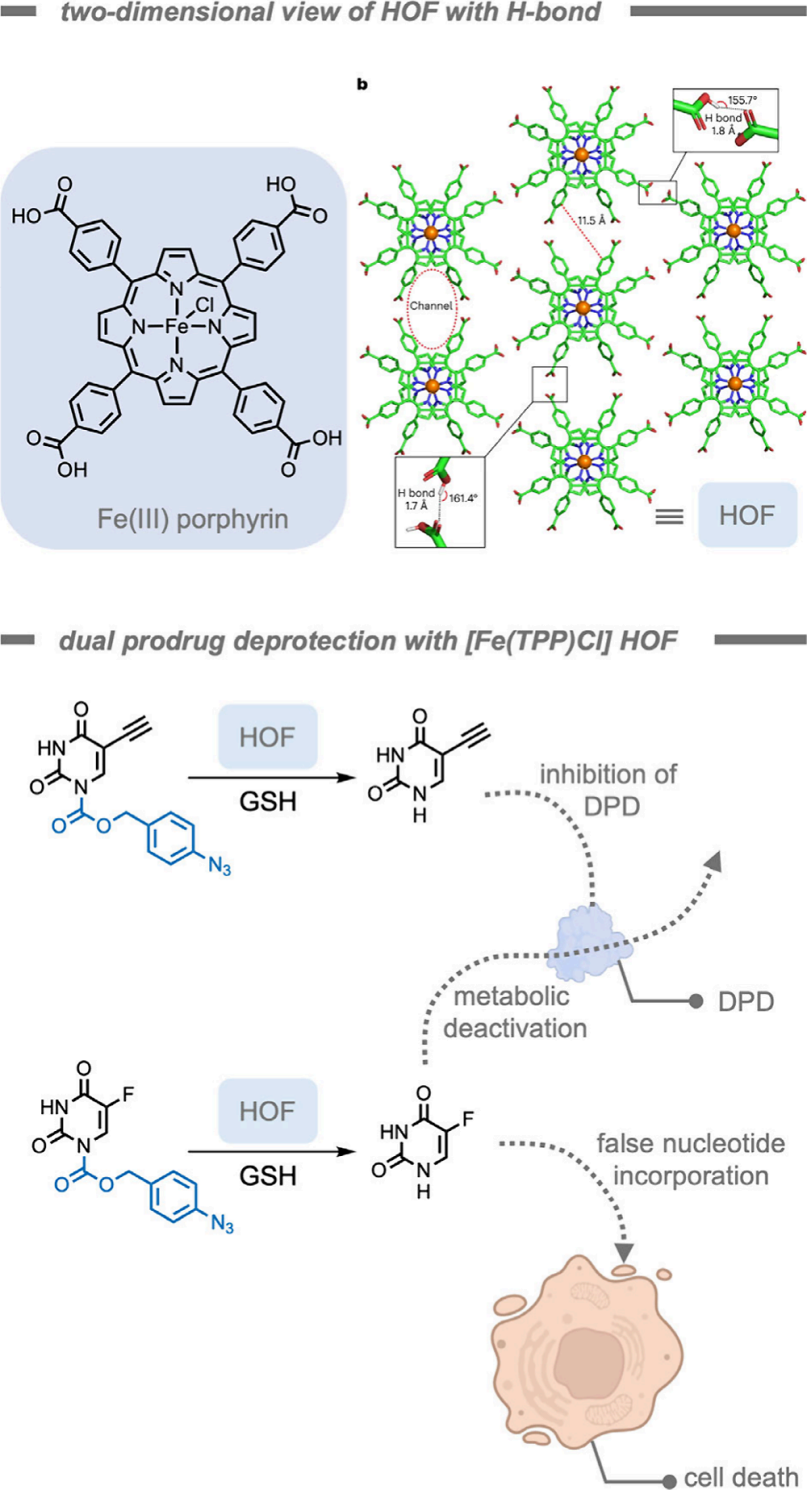
A supramolecular hydrogen-bonded organic framework to release and cleave two prodrugs.^255^ 5-Fluorouracil (5FU) is an active anticancer drug but metabolically inactivated by dihydropyrimidine dehydrogenase (DPD) in the body. 5-Ethynyluracil inhibits DPD and allows for higher clinical efficacy of 5FU. Reproduced in part with permission from ref 255. Copyright 2023, Springer Nature.

Follow-up research by the same group has led to the development of an iron porphyrin-based HOF to target mitochondria for activation of a DNA methyltransferase I inhibitor *in vivo* (Figure 38).^256^ A targeting group for tumor cell mitochondria was generated by reacting the acidic groups on the outside of the HOF with (3-aminopropyl)­triphenylphosphonium bromide (APTPP). The designed HOF catalyzed the activation of artemisinin, which can produce large amounts of ROS to damage mtDNA more effectively and kill cells. The activated artemisinin caused severe cell dysfunction and apoptosis within mitochondria. Hypomethylated mitochondrial DNA (mtDNA) is more sensitive to ROS damage. To circumvent the cell’s DNA hypermethylation defense strategy—which increases oxidative stress resistance—azide-protected procainamide was activated to inhibit DNA methyltransferase 1 (DNMT1) activity. The procainamide prodrug was loaded within the HOF structure. *In vivo* studies in orthotopic metastatic models of Balb/c mice indicated that APTPP@HOF NPs accumulated in the tumor instead of other tissues, most likely due to the EPR effect. Additionally, the tumor growth suppression effect of artemisinin in combination with the protected procainamide-loaded APTPP@HOFs (∼80 mm^3^) was significantly higher than those of the control groups within 14 days: ∼550 mm^3^ for PBS, ∼300 mm^3^ for APTPP@HOFs, ∼450 mm^3^ for APTPP@HOFs+protected procainamide, and ∼250 mm^3^ for APTPP@HOF+artemisinine. In conclusion, this HOF-based platform allowed for the synergetic delivery of a ROS-producing anticancer drug while simultaneously activating a prodrug to make the mtDNA more susceptible to ROS damage.

**Figure 38:**
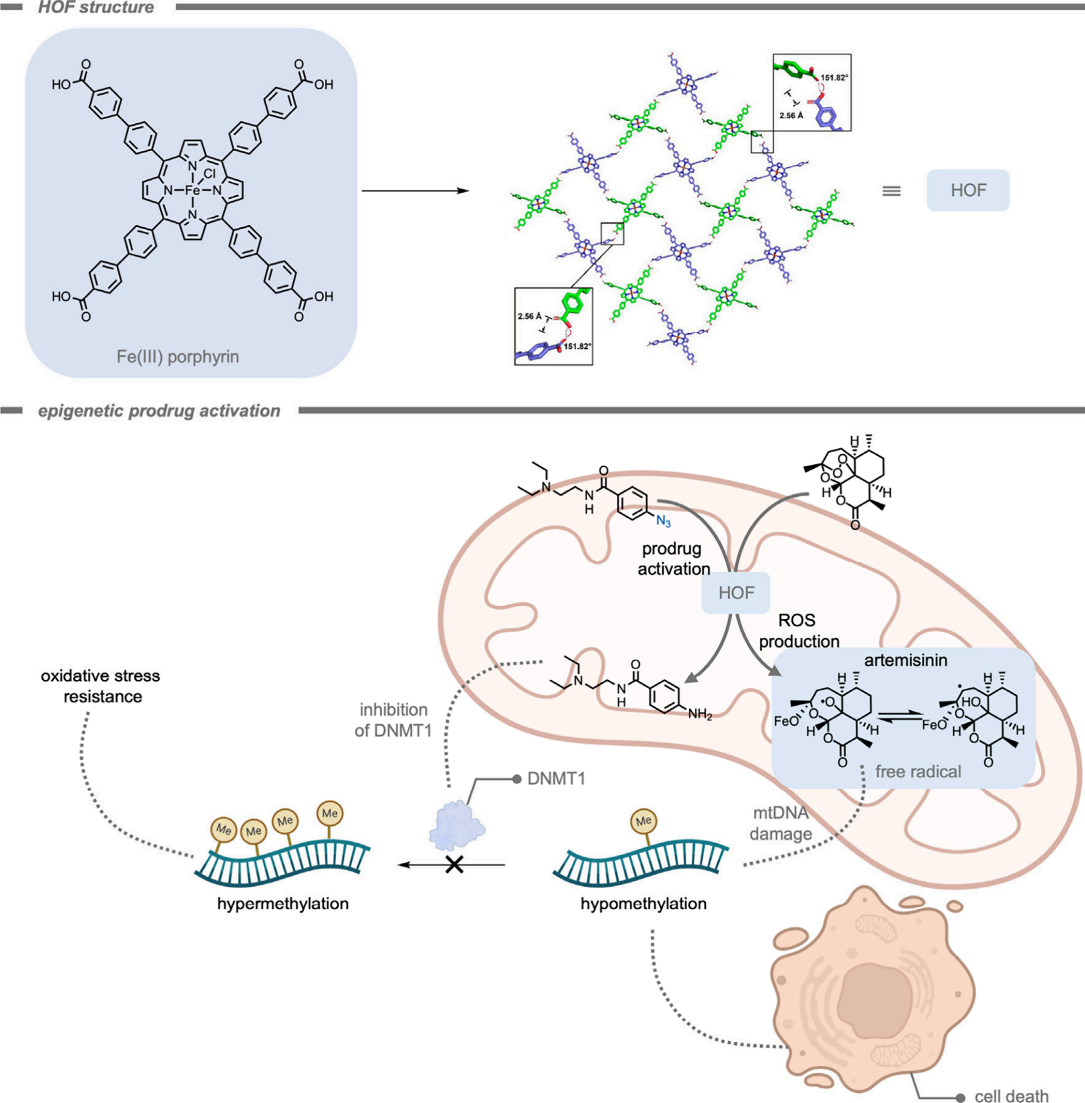
A mitochondria-targeting hydrogen-bonded organic framework for epigenetic prodrug activation and reactive oxygen species production *in situ*.^256^ Reproduced in part with permission from ref 256. Copyright 2024, ACS Publications.

While this manuscript was in submission, Hu, Willner, Wang, and co-workers^257^ developed a supramolecular system containing ultrafine Pd NPs confined within discrete MOCs, which were in turn further functionalized with a glucose oxidase and AS1411 aptamer-modified HA. Their system demonstrated improved tumor nucleolin-targeting ability as well as a 35-fold improved bioorthogonal catalytic activity (>99% in 15 min) for the depropargylation of pro-5FU in aqueous media compared to the control Pd NPs.

## 5 Supramolecular Structures for Reducing the Toxicity of Non-natural Reaction Components

The CuAAC reaction is a widely studied bioorthogonal reaction used to couple diverse complex molecules in biological settings. However, its practical application in living systems is limited by the need for large doses of copper, which leads to significant toxicity.^258^ For instance, Cu^I^ can trigger the formation of ROS, causing extensive cellular damage. To address this challenge, extensive research has focused on reducing copper dosages through strategies such as ligand design or by employing (supramolecular) NPs to improve catalyst activity, which will be discussed in the following section.

For a Cu-free click-reaction, Francis and co-workers^119^ were inspired by earlier work from Mock and co-workers^259,260^ as well as Stoddart and co-workers^261^ on CB[6]-promoted azide–alkyne cycloaddition, which was applied for rotaxane synthesis. The authors adapted this CB[6]-promoted reaction to increase the biocompatibility (Figure 39). CB[6] aligns the azide and alkyne substrates to facilitate the formation of the triazole product. The authors reported 2-[Bis­(2-hydroxyethyl)­amino]-2-(hydroxymethyl)­propane-1,3-diol (BisTRIS) to be the optimal buffer for this reaction, leading to near quantitative conversion within 24 h at 37 °C. In buffers that contained a high concentration of small ions (e.g., Na^+^, and K^+^), the speed of the reaction was significantly decreased, although the reaction still occurred. Interestingly, the CB[6]-click reaction was also demonstrated for the conjugation of drug molecules (e.g., doxorubicin-SS-propargylamine) to NPs. This conjugation proceeded to 99% conversion after 1 h at 37 °C. These conjugates could then subsequently be treated with dithiotreitol to cleave the disulfide linkage and release the doxorubicin for ∼90% after 2 h at ambient temperature. This versatile conjugation strategy can be applied to a large variety of complex bioproteins and coupling partners. For an overview of click reactions with CDs, the reader is referred to a review by Payamifar and Marjani.^262^

**Figure 39:**
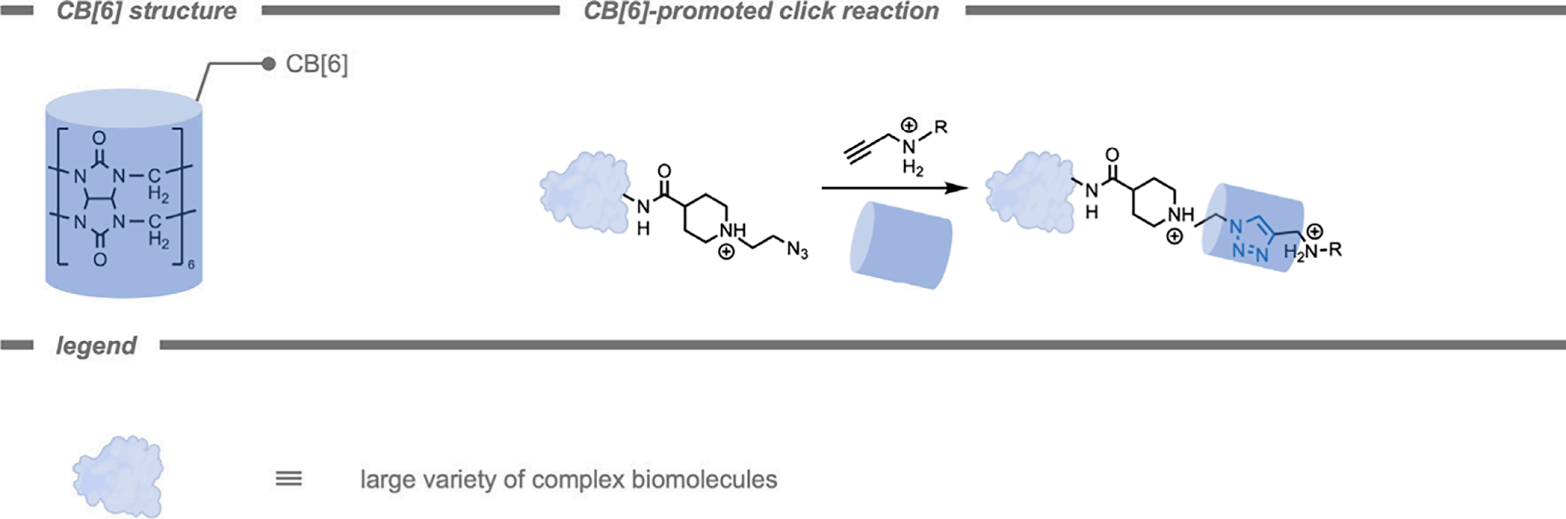
Cucurbit­[6]­uril-promoted azide–alkyne cycloaddition.^119^

In 2023, Yang, Yang, and co-workers^258^ reported a CD-based supramolecular catalyst for the CuAAC reaction (Figure 40). The authors hypothesized that the strong coordination ability of the triazole groups to Cu and the ability of the CD units to preorganize the substrates and catalyst would improve the catalytic activity in aqueous conditions. At low catalyst loadings (10 ppm) this catalyst demonstrated high efficiency (TON of 80000) in aqueous solution, while conversion and yield remained relatively stable at 90 and 80%, respectively. The authors reused the catalyst system in five consecutive CuAAC reactions, after which the yield dropped from 90 to 75%, most likely due to catalyst loss during the recycling process. Moreover, the catalyst was applied for the synthesis of fluorescent probes in extracellular catalysis. In HeLa cells, the CD-based catalyst demonstrated improved extracellular catalytic activity compared to a free Cu^I^ TMC in the click-synthesis of a coumarin-based probe that subsequently diffused in cells. Without catalyst, no fluorescence was observed.

**Figure 40:**
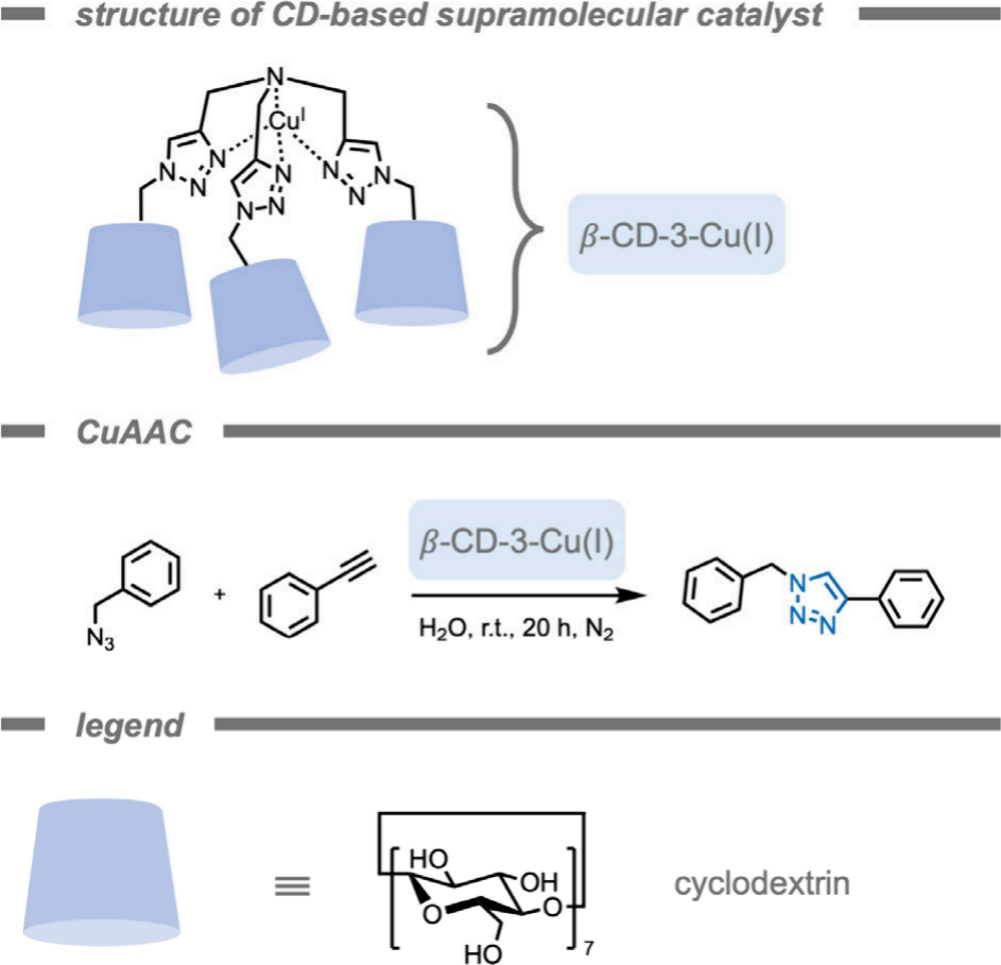
A cyclodextrin-based supramolecular catalyst for aqueous Cu^I^-catalyzed [3+2] azide–alkyne cycloadditions at low catalyst loading.^258^

An alternative strategy reported in literature to improve the efficiency of CuAAC reactions involves enhancing the bioavailabililty of endogenous Cu^I^ ions. However, this strategy is challenging, particularly in cancer cells, where fluctuations in intracellular GSH metabolism often result in varying Cu^I^ levels.^263^ Additionally, the naturally low levels of endogenous Cu^I^ in cells are typically insufficient to efficiently drive the CuAAC reaction, demanding innovative methods to boost endogenous Cu^I^ levels for bioorthogonal applications.

Pu, Ren, Qu, and co-workers^264^ demonstrated the use of MOF (ZIF-90) NPs for simultaneous loading of two drug precursor molecules and sodium ascorbate (Figure 41). The NPs specifically target tumor tissue through the adenosine triphosphate (ATP) aptamers that were attached to the surface. Upon reaching the tumor tissue, the aptamer was dissociated by specific interaction with ATP, and the NPs were subsequently decomposed due to interactions between the Zn^2+^ ions and ATP. The authors stated that sodium ascorbate efficiently converted intracellular copper species to Cu­(I), accelerating the CuAAC reaction for resveratrol drug synthesis *in vivo*. The therapeutic performance of the MOF-based system was demonstrated in 4T1 (breast cancer) tumor-bearing mice models through intravenous injection. In 14 days, only ZIF-90@P-A NPs significantly inhibited tumor growth (∼100 mm^3^) compared to the controls: ∼900 mm^3^ for PBS, ∼900 mm^3^ for ZIF-90, ∼800 mm^3^ for resveratrol precursors, and ∼750 mm^3^ for ZIF-90 + resveratrol precursors. They concluded that MOFs covered with targeting aptamers and loaded with resveratrol precursors as well as sodium ascorbate allowed for the enhancement of endogenous Cu^I^ species, facilitating intracellular drug synthesis with high anticancer efficacy.

**Figure 41:**
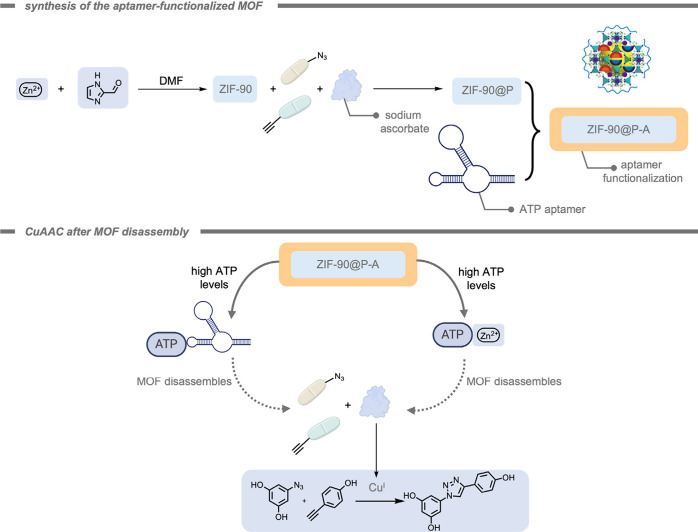
An adenosine triphosphate aptamer-functionalized ZIF-90 metal–organic framework for encapsulation of prodrugs and sodium ascorbate to enhance the Cu^I^-catalyzed [3+2] azide–alkyne cycloaddition reaction in tumor cells.^264^ Reproduced in part with permission from ref 264. Copyright 2023, ACS Publications.

In 2024, Qu and co-workers^265^ further developed the application of MOFs for the transport of copper. The authors developed ZIF-8-Pt^IV^-folic acid NPs to selectively bind Cu ions through a weak coordination bond between the imidazole ligands and Cu ions (Figure 42). In acidic environments, the Cu ions were unloaded due to reduced interactions between the imidazole and Cu. According to the authors, the NPs could selectively transport Cu to tumor cells through folic acid receptor-mediated endocytosis. Simultaneously, the Pt^IV^ prodrugs attached to the MOF were reduced to active cisplatin (Pt^II^) by the high intracellular GSH concentrations. The increased levels of Cu^I^ in the tumor cells in turn accelerated the CuAAC reaction, allowing for *in vivo* prodrug activation. The therapeutic efficacy of the ZPF platform was investigated on B16F10 (melanoma) tumor-bearing mice models. In terms of tumor growth suppression and survival rate, ZIF-8-Pt^IV^-folic acid NP-mediated prodrug activation (∼50 mm^3^) outperformed all control groups: ∼1250 mm^3^ for PBS, ∼700 mm^3^ for resveratrol precursors, ∼500 mm^3^ for ZIF-8-Pt^IV^-folic acid NPs only, ∼400 mm^3^ for resveratrol precursors + Pt^IV^, and ∼200 mm^3^ for resveratrol precursors + ZIF-8-Pt^IV^ NPs. These results highlight the potential of increasing Cu^I^ levels to increase concentrations of resveratrol—generated through the CuAAC reaction—subsequently improving the antitumor effect.

**Figure 42:**
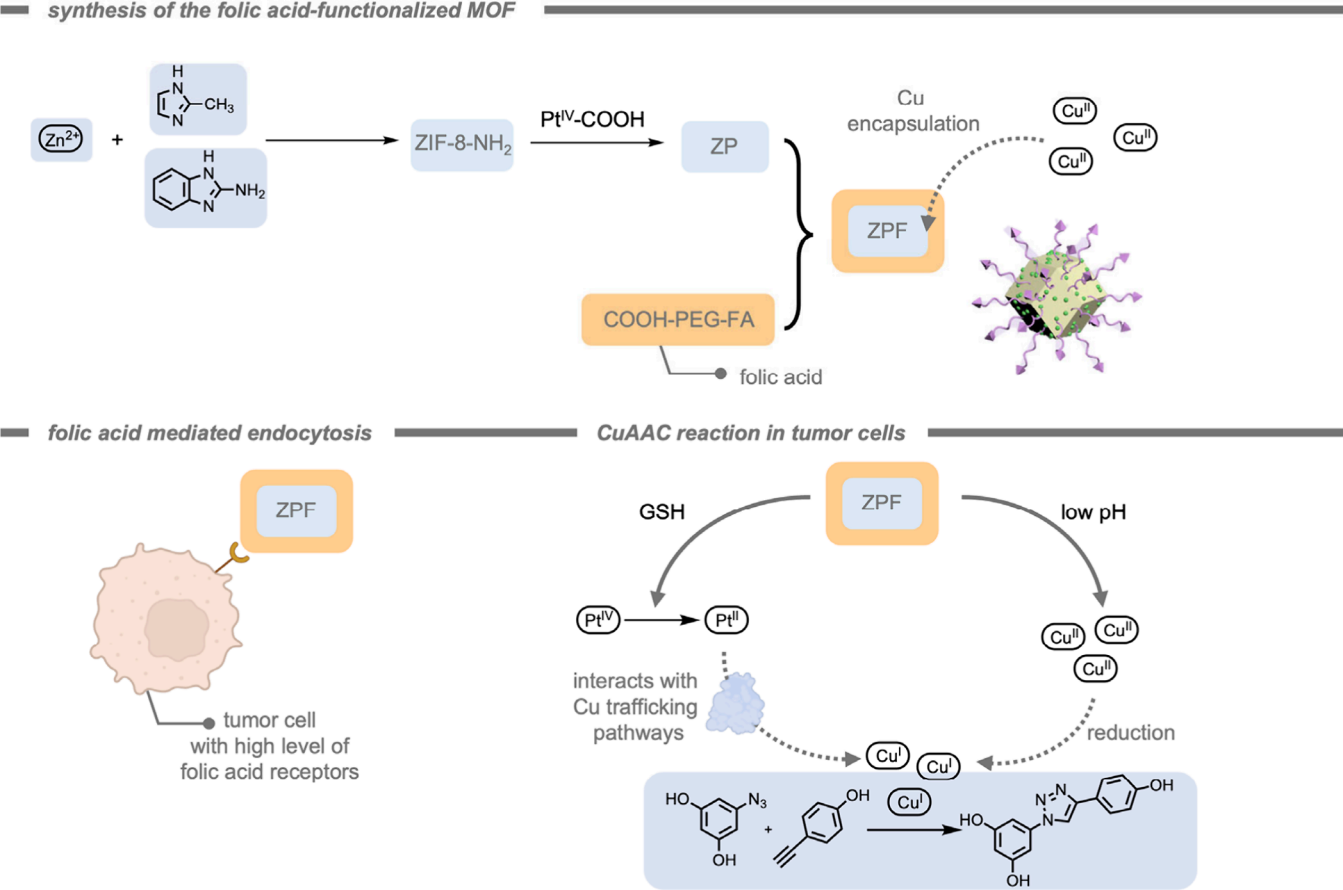
A metal–organic framework-based Cu transporter that selectively releases Cu ions within tumor cells, locally catalyzing the Cu^I^-catalyzed [3+2] azide–alkyne cycloaddition reaction.^265^ Reproduced in part with permission from ref 265. Copyright 2024, ACS Publications.

Kumar, Cho, and co-workers^266^ demonstrated the use of human platelet membrane vesicles for the encapsulation of [Fe­(TPP)­Cl] catalysts. These encapsulated catalysts were applied in treatment of dental caries caused by oral biofilm (Figure 43). Most current antimicrobial treatments are broad-spectrum agents that are nonspecific and limited in their efficiency. Platelets participate as the first line of defense against microbial infections by releasing antimicrobial agents.^267^ Bacteria bind to platelets either directly or through surface receptors (e.g., bacterial *N*-formylated peptides bind to platelet formylpeptide receptor) by forming a plasma-bridging molecule. For *in situ* synthesis of antimicrobial agents, platelets play a vital part in connecting to bacterial membrane proteins for host-defense mechanisms. The authors demonstrated the use of platelet membrane vesicles to deprotect aryl azide carbamate-protected ciprofloxacin into its active antimicrobial form on oral biofilms of *S. mutans* 3065, the main cause of tooth decay. The platelet reactor was demonstrated to be colocalized with the bacteria and the bacteria-produced extracellular matrix of macromolecules with a ∼87 μm penetration in a biofilm of ∼94 μm thick. Upon addition of pro-ciprofloxacin (1 mM), a significant decrease in the number of live bacteria (∼98%) and degradation of the extracellular matrix (∼93%) was observed, indicating *in situ* antimicrobial and antibiofilm effects through catalytic properties of the platelet reactor.

**Figure 43:**
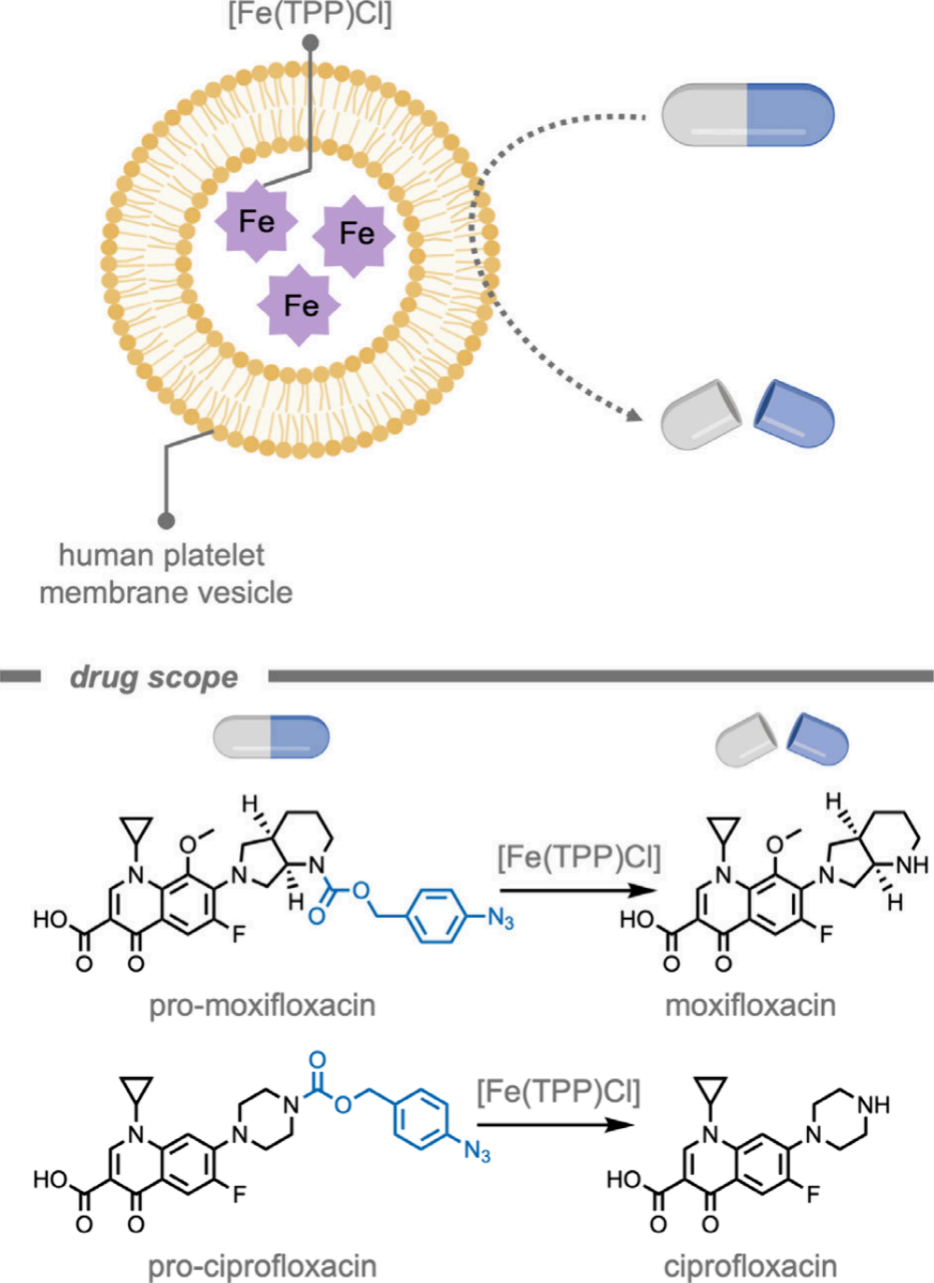
Human platelet membrane vesicle reactors encapsulating [Fe­(TPP)­Cl] catalysts for the on-site activation of pro-antibiotics.^266^

## 6 Preorganization of Substrates within a Supramolecular Structure and Confinement Effects

Over the last decades, MOCs have been investigated as a tool to control the activity and selectivity of a diverse set of catalytic reactions. The confinement effects imposed by the supramolecular structure can enhance the selectivity and/or activity of reactions by various mechanisms. For comprehensive works on the confinement effects within discrete, self-assembled hosts, the reader is referred to various reviews.^8,11,34,37,48,93,231,268–273^ Next to supramolecular organization in MOCs, supramolecular interactions can be employed to precisely position the substrate molecule with respect to the metal center, leading to higher selectivity control in chemical transformations.^95^ This preorganization effect can also lower the activation barriers for specific pathways, thereby also changing the reaction rate.

Supramolecular hosts have been employed to control the reactivity of bioorthogonal azide moieties in aqueous solutions. Azides have widespread and varied use in biological settings due to their unique reactivities. One of the pathways through which aryl azides can be employed is photolysis, in which the UV-promoted release of a N_2_ molecule results in the formation of a highly reactive singlet nitrene intermediate. This singlet nitrene can subsequently undergo three reaction pathways: (i) the amination of a X–H bond (X = C, N, O, or S), (ii) an intramolecular rearrangement to ketenimines, and subsequent nucleophilic insertion to release azepine derivatives, and (iii) intersystem crossing to the triplet nitrene, resulting in dimerization to azobenzenes. Steering the reactivity of nitrenes is highly challenging, especially in aqueous solution as water can perform a nucleophilic attack. To improve the selectivity of this reaction in aqueous solution, Biedermann, Bräse, and co-workers^274^ employed a supramolecular CB[7] host as a reaction vessel (Figure 44). The hydrophobic cavity of the CB[7] hosts protects the photogenerated intermediates for attack of water, which allowed for the selective synthesis of a carboline derivative. As this carboline derivative is blue-fluorescent when excited at ∼460 nm both in its free and encapsulated form (Φ_FL_ of 77% and 90%, and lifetimes of 21.9 and 25.4 ns, respectively), the authors demonstrated their use as blue-fluorescent labels for the staining of HeLa cells. In the solid state, the CB[7]-encapsulated carboline product revealed long-lived room temperature phosphorescence, with lifetimes up to 2.1 s. Although *in vitro* and *in vivo* use of this reaction has not yet been explored, these findings underscore the potential of supramolecular hosts to modulate the photolysis of aryl azide bioorthogonal groups to facilitate fluorescent materials for cell imaging.

**Figure 44:**
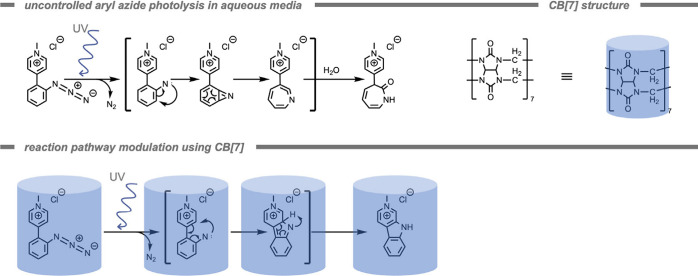
Uncontrolled aryl azide photolysis in aqueous media. Modulation of the reaction pathway of aryl azide photolysis using a cucurbit[7]­uril host.^274^

In 2020, Mancin, Salassa, and co-workers^275^ reported the use of an AuNP modified with a 1,4,7-triazacyclononane (TACN)-containing monolayer for the photocatalytic conversion of a Pt­(IV) prodrug into cisplatin (Figure 45). The protonated TACN headgroup can bind the negatively charged phosphate group on the riboflavin-5′-phosphate (FMN) photocatalyst, as well as the negatively charged carboxylate groups on the Pt­(IV) ligands. The authors proposed that the AuNP would be able to colocalize substrate and photocatalyst within the monolayer. Investigations in water demonstrated binding of FMN to the AuNP, with a saturation concentration of 50–60 μM. However, catalytic investigations in 2-(*N*-morpholino)­ethanesulfonic acid (MES) buffer did not show significant difference between the catalytic activities of FMN@AuNP and free FMN (90% conversion in 30 min of light irradiation). Selective preorganization to enhance catalysis in the presence of buffers containing ionic species is still highly challenging, as these ions are present in excess compared to the catalyst and substrate. Further studies should elucidate the preorganization of substrate and catalyst in the presence of buffers containing anionic species, as these can compete for binding to the TACN headgroups. Furthermore, the authors reported a strong background conversion of TACN AuNPs toward the photoreduction of the Pt­(IV) prodrug (50–70% conversion in 30 min of light irradiation). This finding underscores the necessity of a systematic investigation into the specific role of the AuNP scaffold for the chemical transformations of interest.

**Figure 45:**
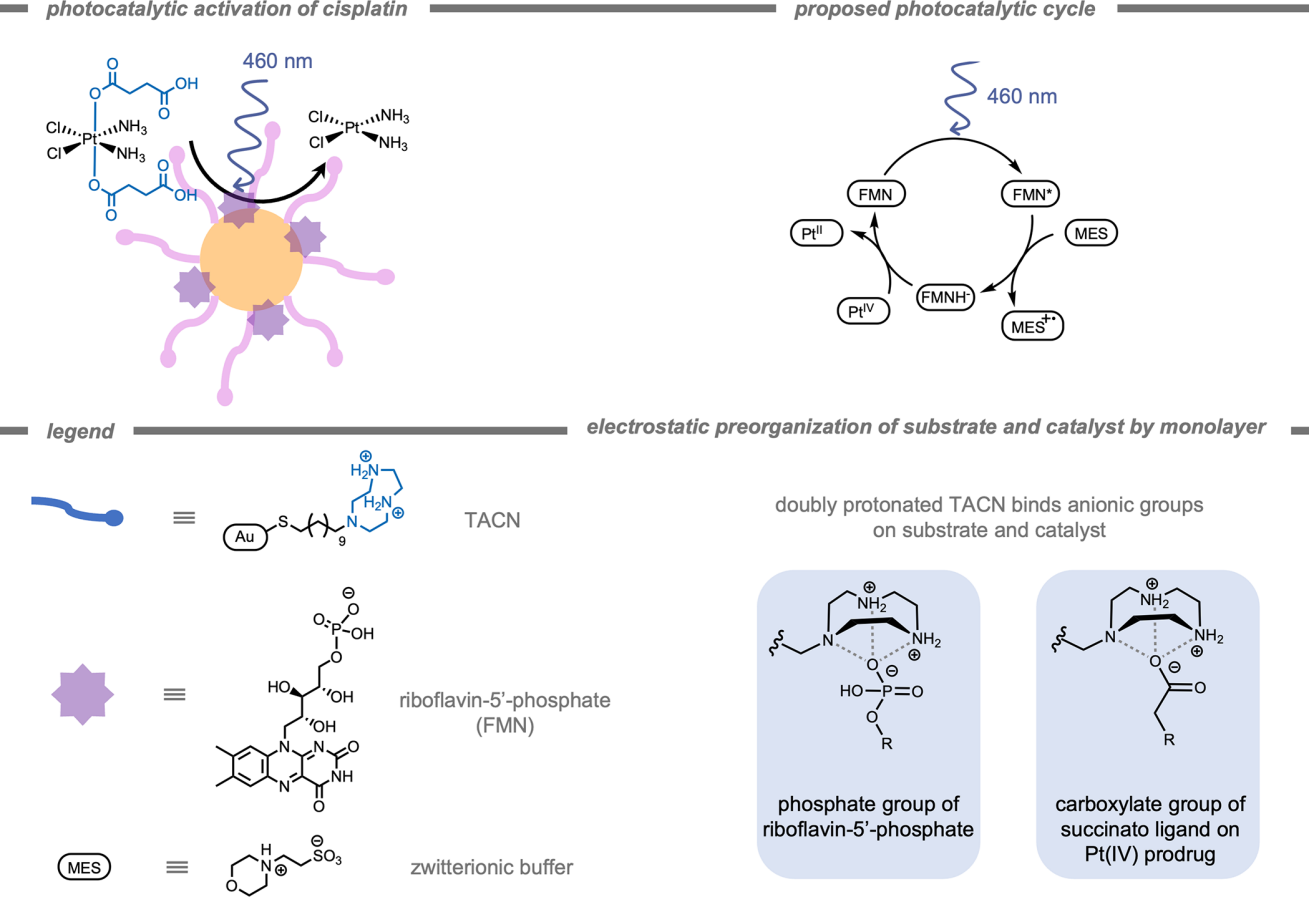
Photocatalytic activation of a Pt­(IV) prodrug on a gold nanoparticle using a preorganized riboflavin-5′-phosphate photocatalyst.^275^

By employing the unique properties of an AuNP core, Mascareñas, del Pino, and co-workers^276^ reported plasmonic-assisted NIR-driven thermocyclization in living cells. They developed a NP with an Au nanostar core, embedded within a ZIF-8 MOF. Once inside the cell, the MOF cloak allowed for efficient diffusion of the reactants to the Au core, where they were converted upon NIR-illumination, while blocking larger molecules from reaching the core. The authors demonstrated that these supramolecular plasmonic NPs can allow for the use of thermal-driven processes (e.g., an intramolecular cyclization that typically requires heating at ∼90 °C in bulk) in living cells.

In 2025, Papot, Taran, and co-workers^277^ reported the use of micelles constituted by sydnonimine-based amphiphiles to synthesize sorafenib, an FDA-approved drug, in living cells (Figure 46). Previously, they reported the use of sydnonimines to release isocyanates upon reaction with cyclooctynes, called the strain-promoted sydnonimine cycloalkyne (SPSIC) cycloaddition reaction. By generating the isocyanates inside the confined environment of the micelle core, a selective reaction with encapsulated amines for the synthesis of ureas was observed. This selective urea formation occurred despite the presence of surrounding endogenous nucleophiles. The control reaction in MeOH/H_2_O yielded only a small amount of the urea product due to significant hydrolysis of the isocyanate. The sydnonimine micelle system was then further investigated in HepG2 (hepatocellular carcinoma) tumor cells for the generation of sorafenib. Tumor cells were incubated with micelles and, subsequently, a dibenzocyclooctyne (DBCO) reagent. Addition of the DBCO reagent to the cells previously incubated with micelles loaded with the amine-containing prodrug triggered a strong antiproliferative activity, comparable to cells incubated with sorafenib. In contrast, cells incubated with free amine-containing prodrug or empty micelles did not yield significant cellular toxicity, either when incubated alone or with subsequent DBCO incubation.

**Figure 46:**
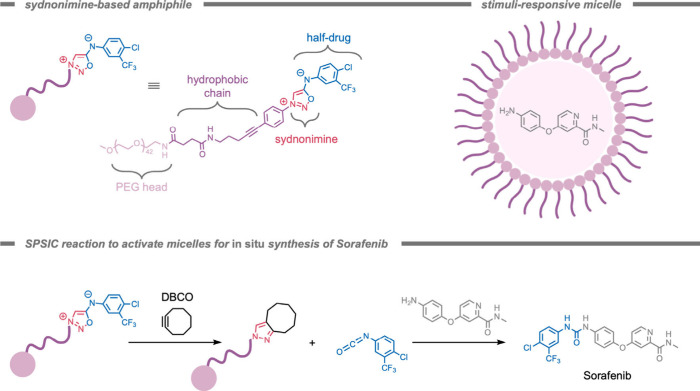
Stimuli-responsive sydnonimine-based micelles for *in vitro* synthesis of sorafenib by preorganization of a reactive isocyanate and amine inside the confined environment of the micelle core.^277^

Another example of click chemistry promoted by confinement effects in supramolecular MOCs was demonstrated by Kim and co-workers.^278^ The authors reported a significant enhancement in the reactivity between fullerenes (C_60_/C_70_) and 1,2,4,5-tetrazines inside a porous Zn-porphyrinic MOC. The IEDDA reaction between these two substrates has rarely been explored due to the low reactivity, therefore requiring harsh reaction conditions. To confine the reactants, a previously reported Zn-porphyrin-based MOC was employed, consisting of six porphyrins interconnected by eight triamines. In a solvent mixture of 5% water in organic solvents after 72 h at r.t., the conversion catalyzed by the Zn-MOC was ∼6-fold increased (∼60%) compared to the controls (∼10%). The use of supramolecular structures to improve selectivity and kinetics of bioorthogonal reactions through confinement effects and preorganization has not been widely reported in literature. Most examples mentioned in this section have not been demonstrated beyond biorelevant media. So far, substrate preorganization through hydrophobic interactions seems most promising for the implementation in aqueous systems or cells. Preorganization through electrostatic interactions is highly challenging, as ionic species can compete for the binding event in buffered systems. Although still in its infancy, substrate preorganization is a promising strategy to enhance selectivity in highly complex biological media.

## 7 Stimuli-Responsive Catalytic Sites on a Surface

Another powerful tool is controllable catalysis by stimuli-responsive supramolecular systems. These stimuli can be external (e.g., light, heat, magnetic field, electric field, or ultrasound) or internal (changes in environment, such as pH, redox gradients, biomolecule concentrations, or ionic strength).^279^ Various stimuli-responsive supramolecular systems have been developed over the past decade to regulate catalytic properties in biological systems.

Surface-modified AuNPs can host catalysts for various biorelevant reactions.^171^ For example, in 2015, Rotello and co-workers^280^ synthesized a bioorthogonal NP that contained an encapsulated [Cp*Ru­(cod)­Cl] in the monolayer of water-soluble AuNPs (Figure 47). A supramolecular CB[7] bound on the monolayer surface could reversibly control the activity of these catalysts by acting as a ‘gatekeeper’. When the CB[7] is noncovalently bound to the surface of the AuNP, the substrate could not reach the catalyst on the AuNP surface due to steric hindrance. The catalytic activity of the AuNP was completely inhibited in the presence of CB[7] with a decrease of reaction rate (expressed in intensity/min) from ∼10 to ∼0 in solution. Isothermal titration calorimetry indicated that per NP, ∼60 CB[7] molecules were bound with K_d_ = 11.3 μM. When a competitive 1-adamantylamine guest was added to the system, CB[7] detached from the AuNP surface through guest–guest exchange. As a result, the steric hindrance was removed, thereby regaining the catalytic activity of the overall system (reaction rate of ∼11 in intensity/min). The authors further demonstrated the potential of this strategy by regulating the allyl carbamate cleavage of a rhodamine pro-fluorophore as well as the deprotection of propargyl-protected 5FU. *In vitro* studies in HeLa cells demonstrated that at the highest concentration of pro-5FU (1.00 mM), cell viability remained high for the control group (∼105%) and the group incubated with CB[7]-gated AuNP (∼95%). Significant toxicity effects were observed for the groups incubated with active NPs (∼10%) as well as the group incubated with CB[7]-gated NPs + 1-adamantylamine (∼20%), indicating an efficient gating resulting in catalytic activation of prodrugs within HeLa cells.

**Figure 47:**
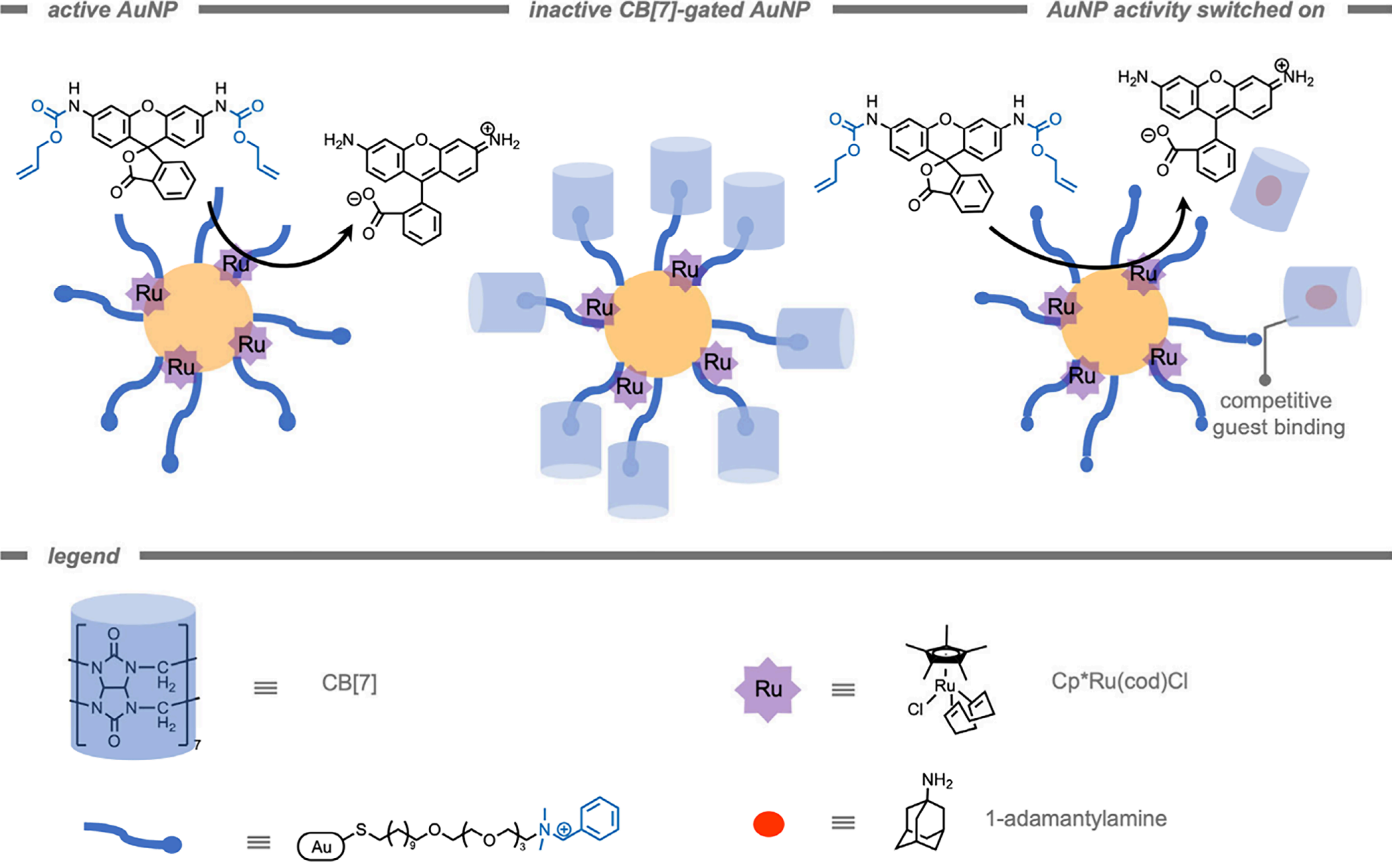
Cucurbit­[7]­uril as supramolecular ‘gatekeeper’ on a gold nanoparticle surface to regulate the rate of the cleavage of allyl carbamate protecting groups for pro-fluorophore activation.^280^

Qu and co-workers^281^ employed supramolecular complexes for reversible light-controlled bioorthogonal catalysis in living cells. The authors modified silica-Pd^0^ particles with azobenzene to protect the NP surface through the formation of a supramolecular complex by binding the β-CD building blocks to the azobenzene (Figure 48). The catalytic activity of the system was inhibited by the CD cap, because access to the catalytic site was blocked. Upon UV irradiation, azobenzene underwent E/Z isomerization, which led to release of the CD cap. The catalytic activity of the system was switched on by making the active sites accessible. The authors demonstrated this effect through deallylation of a pro-fluorophore, but also through a Suzuki–Miyaura cross-coupling reaction to synthesize a mitochondria-specific probe *in vitro*. Further studies focused on the light-gated activation of propargyl-protected 5FU. HeLa cells incubated with pro-5FU (500 μM) and CD-inhibited NPs demonstrated relatively high cell viability (∼85%) compared to the controls: ∼100% for PBS, ∼95% for CD-inhibited NPs, and ∼95% for pro-5FU. Upon irradiation with UV light, thereby switching on the catalytic activity, the cells incubated with pro-5FU and CD-inhibited NPs demonstrated a significant increase in toxicity, leading to only ∼10% cell viability. This toxicity was not inherent to the UV light or catalyst itself, this increase in toxicity was ascribed to light-gated control of the conversion of pro-5FU into 5FU. Moreover, the authors indicated that this catalyst can be switched back and forth using visible and UV light, which would allow for high precision in further therapeutic applications.

**Figure 48:**
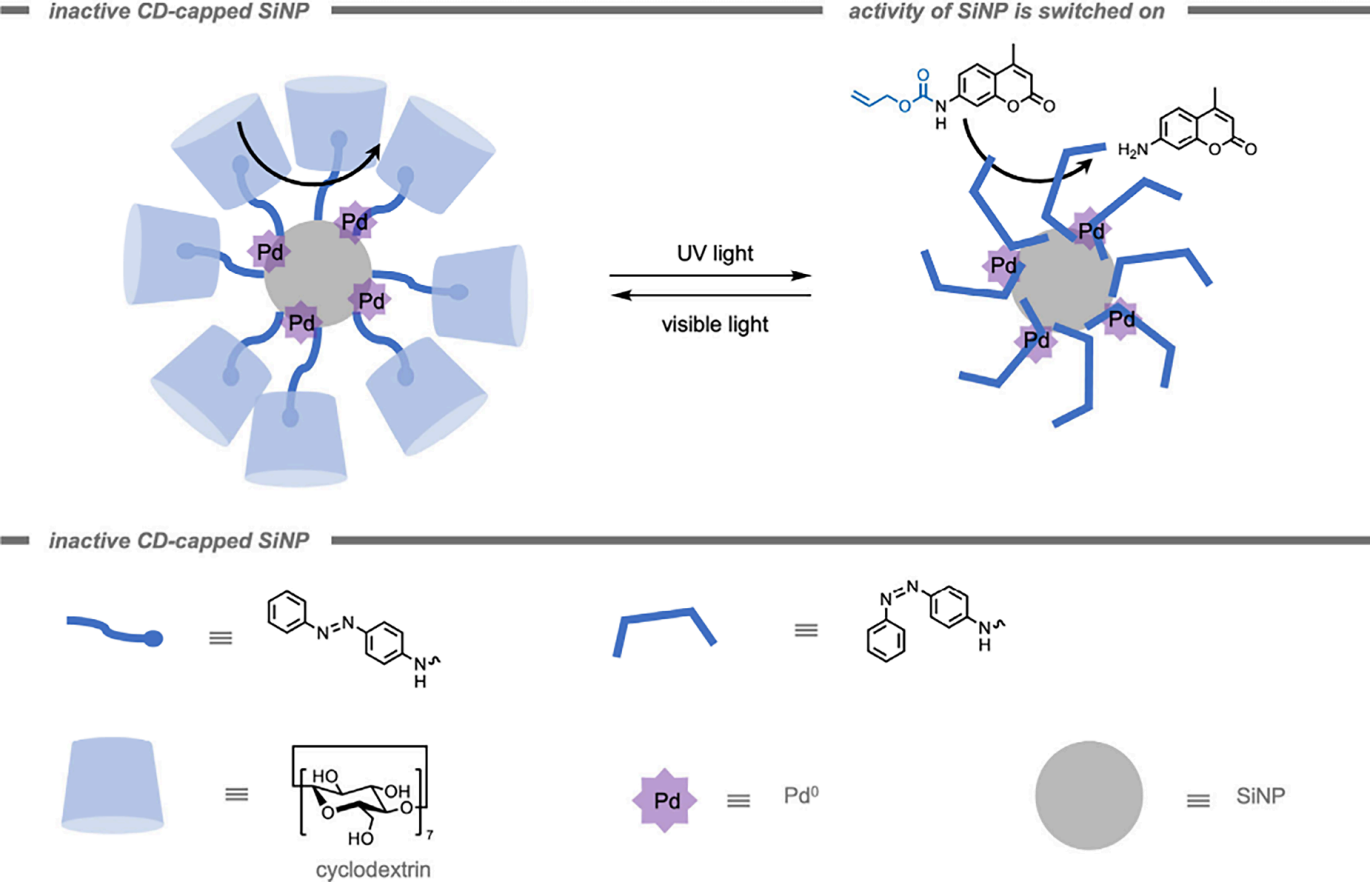
Heterogeneous Pd-catalysts modified with cyclodextrin for controlled photoresponsive bioorthogonal catalysis in living cells.^281^

Temperature can also be employed as a stimulus to control access to catalytic sites. Rotello and co-workers^282^ demonstrated a temperature-regulated supramolecular system consisting of stacked [Fe­(TPP)­Cl] porphyrins into a monolayer-functionalized AuNP (Figure 49). At room temperature (25 °C), these supramolecular porphyrin assemblies interact with the charged ligands on the monolayer, resulting in a compact structure that blocks access to the active site. However, at increased temperatures (e.g., 37 °C) these porphyrin assemblies disassembled and redistributed over the full surface of the monolayer, recovering the catalytic activity. The authors stated that the activation temperature lies within a 3 °C range and is tunable depending on the ratio of the components. The efficacy of this system was demonstrated through the thermal activation of antibacterial prodrugs to treat a bacterial biofilm. The temperature-dependent catalytic properties of the porphyrin-loaded AuNPs by deprotection of an aryl azide carbamate-protected resorufin fluorophore were probed within GFP-expressing *Escherichia coli* (*E. coli*). Negligible fluorescence was observed for biofilms treated with pro-resorufin and AuNPs at 25 °C, whereas significant fluorescence was observed within biofilms treated with pro-resorufin and AuNPs at 37 °C. Further studies demonstrated the temperature-regulated activation of an antimicrobial pro-moxifloxacin derivative. For biofilms incubated with the highest prodrug concentration (4 μM) at 37 °C, the porphyrin-loaded AuNPs significantly decreased the biofilm viability (∼15%), similar to the moxifloxacin positive control (∼12%), whereas the negative control of pro-moxifloxacin only maintained high biofilm viability (∼110%). At 25 °C, the biofilm viability of the group treated with pro-moxifloxacin and porphyrin-loaded AuNPs remained even higher (∼110%) than the negative control (∼90%) and much higher than the positive control (∼20%). Overall, these results demonstrate that confining porphyrin catalysts at the surface of these semirigid AuNPs leads to supramolecular aggregate formation, forming a deactivated state. Increased temperature leads to enhanced activity, therefore providing a temperature-responsive catalytic system that can be employed in biological environments for gated catalysis. Additionally, Rotello and co-workers^283^ have generated thermoresponsive polymer-based catalytic NPs for the deprotection of a pro-antibiotic.

**Figure 49:**
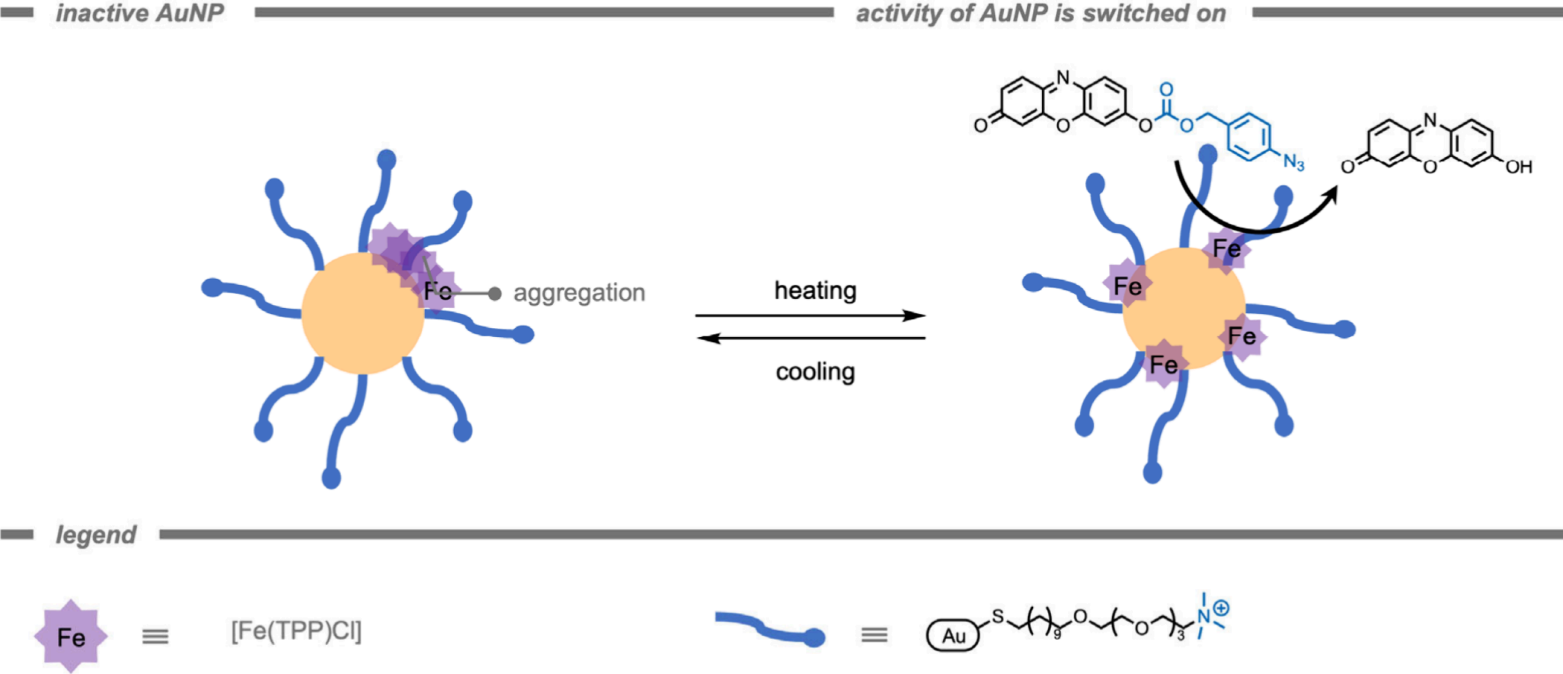
A temperature-regulated supramolecular catalytic system consisting of [Fe­(TPP)­Cl] aggregates on a monolayer of a gold nanoparticle for the catalytic reduction of aryl azides.^282^

In 2024, Liu, Zhang, Fedeli, Cicek, Rotello, and co-workers^279^ presented a stimulus-responsive catalytic system based on reversible supramolecular interactions between charged AuNPs and proteins (Figure 50). The catalytic activity of the Pd TMC-loaded AuNPs (Pd­(dppf)­Cl_2_@AuNP) was allosterically inhibited by enhanced green fluorescent protein (EGFP) by blocking access to the palladium catalyst. Using solutions with different ionic strengths could regulate the catalytic ability of the complex. This effect was attributed to the competitive electrostatic interaction by salt ions, which resulted in the dissociation of the complex and thereby increased accessibility to the Pd active site. Under biorelevant conditions, the authors demonstrated that the fluorescence intensity obtained after 2 h for the catalytic deprotection of propargyl carbamate-protected coumarin halved upon addition of 10 equiv of EGFP to the Pd­(dppf)­Cl_2_@AuNPs. Moreover, the *k*_cat_ of the positive control (Pd­(dppf)­Cl_2_@AuNPs in 5 mM phosphate buffer) was ∼2-fold higher than the negative control (Pd­(dppf)­Cl_2_@AuNP:EGFP 1:1 in 5 mM phosphate buffer). Upon addition of 50 mM NaCl the catalytic rate of the Pd­(dppf)­Cl_2_@AuNP:EGFP 1:1 group was enhanced by ∼12%, and with a further increase to 150 mM NaCl—which corresponds to a normal physiological concentration—the authors observed a ∼50% increase in *k*_cat_ relative to the negative control. Although not yet studied *in vitro* or *in vivo*, this work demonstrates the versatility of gated catalysis and their potential for applications in biosensing or prodrug activation.

**Figure 50:**
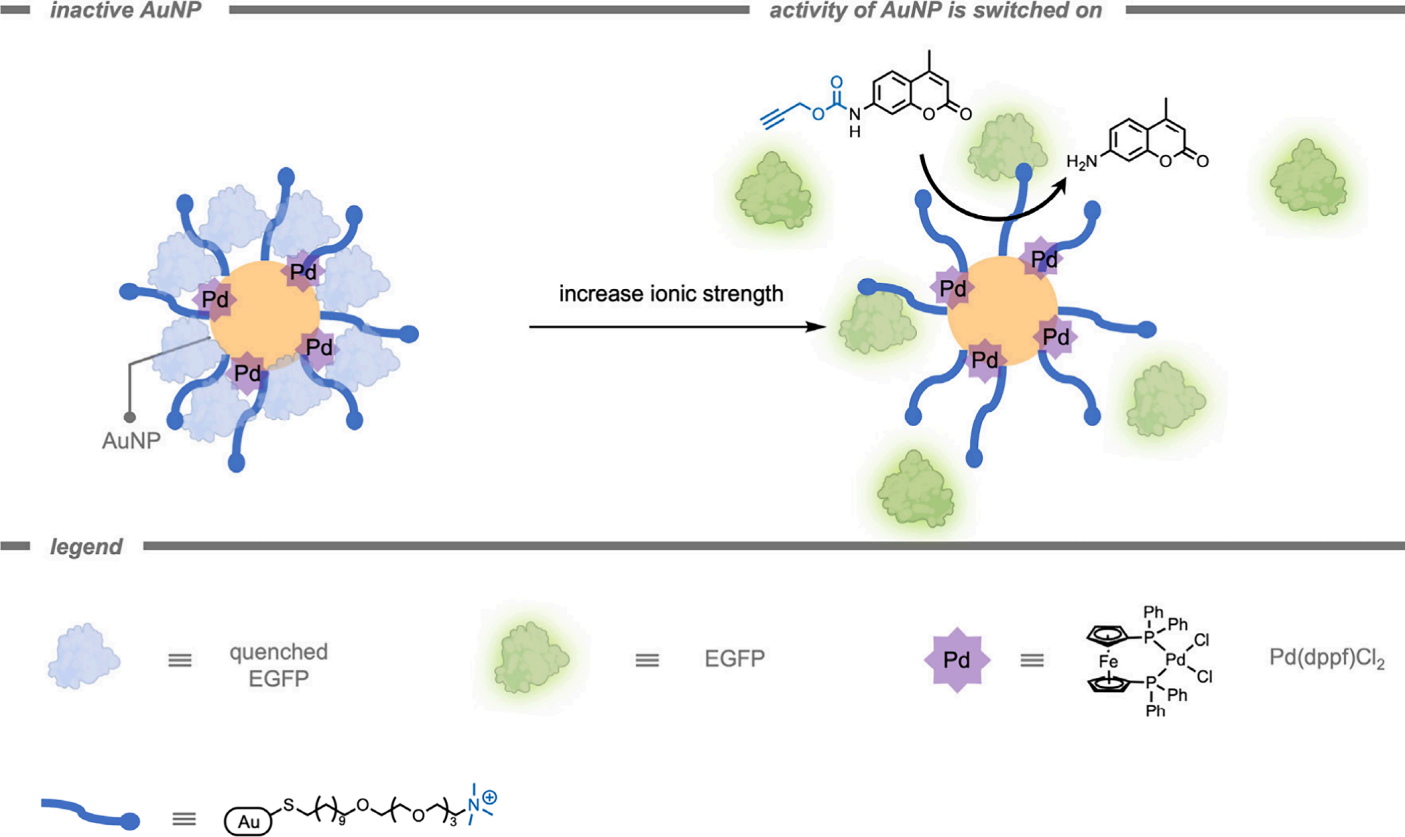
An ionic strength-regulated supramolecular catalytic system based on an enhanced green fluorescent protein–nanozyme complex for the catalytic deprotecting of fluorescent probes.^279^

In 2023, Zhang, Ren, Qu, and co-workers^284^ developed a supramolecular catalytic system that combined various supramolecular strategies discussed throughout this review (Figure 51). They reported a self-assembled nanosized Co@ZIF-8 MOF coated with a monolayer of DNA. This monolayer consisted of mDNA for active targeting of cancer cells and cDNA as switchable units to further cover the surface to inhibit catalysis. The observed inhibition stemmed from blocked access of the substrate to the Pd^0^ catalyst that resided on the surface of the MOF. Under biorelevant conditions, their catalytic system deprotected an allyl carbamate-protected coumarin fluorophore. At physiological pH, cDNA blocked the catalytic activity of the system. At low pH, cDNA folded into the i-motif and dissociated from the MOF, providing the prodye access to the active palladium catalysts. This resulted in switching on the catalytic activity. Moreover, the authors stated that the Co metal centers in the Co@ZIF-8 MOF scaffold could directly consume GSH through oxidation of molecules. This in turn led to GSH depletion, thereby preventing deactivation of the Pd catalyst. Further studies demonstrated the activation of 5FU *in vivo* by subcutaneous murine breast cancer (4T1) tumor-bearing mice. The group treated with the DNA-modified Co@ZIF-8 NPs and pro-5FU demonstrated significant tumor suppression (∼0.07 g) compared to the controls: ∼0.34 g for PBS, ∼0.31 g for pro-5FU, and ∼0.25 g for DNA-modified Co@ZIF-8 NPs. Moreover, to demonstrate that DNA-gated catalysis works under *in vivo* conditions, the authors explored the biodistribution (tumor, liver, kidney, and serum) of generated 5FU in tumor-bearing mice 5 h post injection of DNA-modified Co@ZIF-8 NPs and pro-5FU. The DNA-gated NPs produced less 5FU in kidney, liver, and serum than the non-DNA gated variant. From these results, the authors concluded that the cooperation between the *in vivo* activation of pro-5FU as well as the raised oxidative stress level mediated by the DNA-modified Co@ZIF-8 NPs enhanced the antitumor effect, while the mDNA-targeting and cDNA-gating effects were still active *in vivo.*

**Figure 51:**
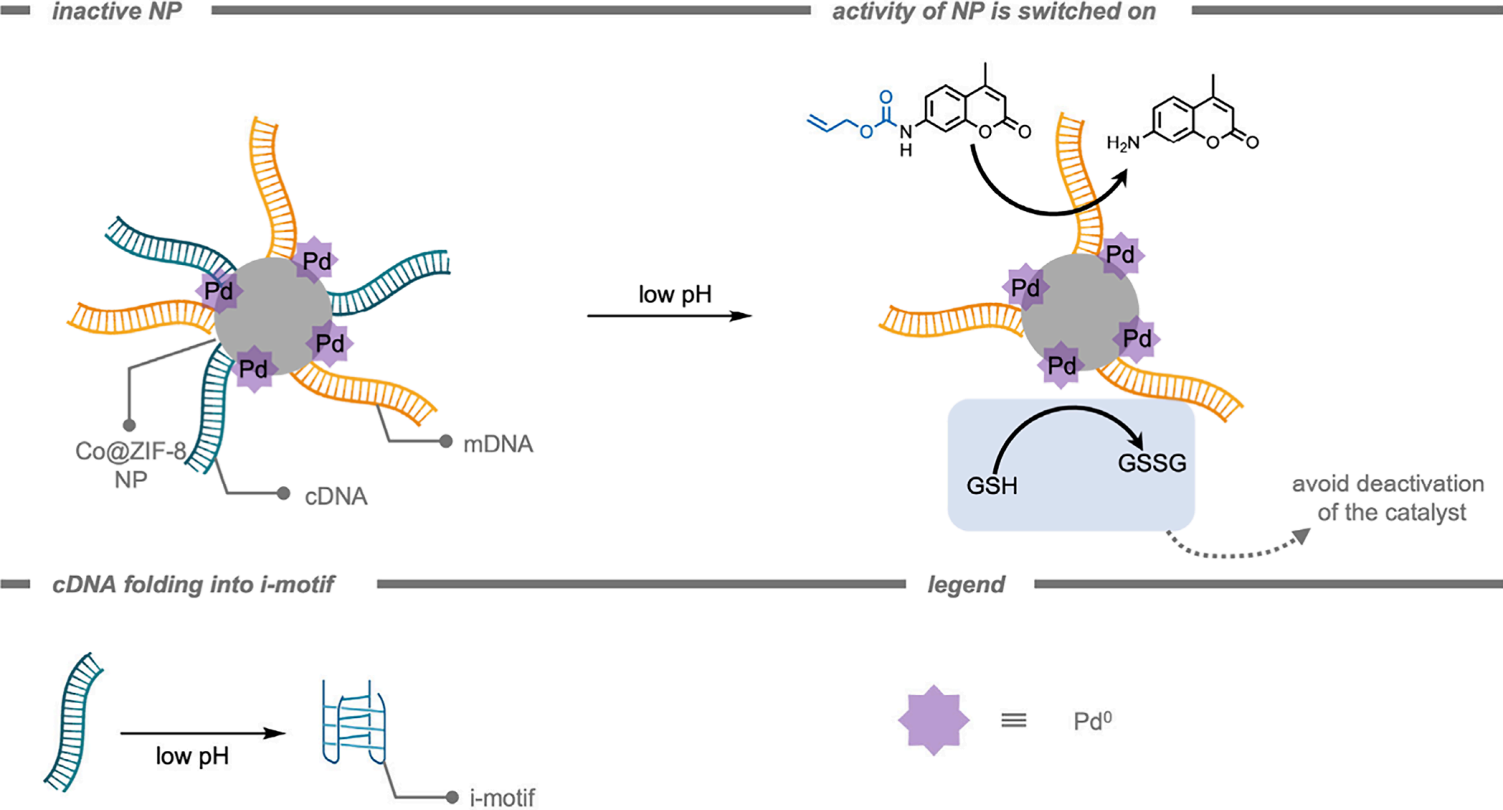
A DNA -gated nanozyme for targeted catalysis in cancer cells while inhibiting catalysis in healthy cells as well as protecting the Pd^0^ catalyst from the biological environment.^284^

Rotello and co-workers^285^ demonstrated the versatility of surface-engineered supramolecular materials containing [Cp*Ru­(cod)­Cl] for bioorthogonal catalysis (Figure 52). Their study showed that the surface functionalization of AuNPs could dictate the localization of the catalyst materials, either intra- or extracellularly. Specifically, cationic NPs that have cell-penetrating capabilities were shown to efficiently facilitate intracellular catalysis, whereas zwitterionic particles restricted the catalytic activity to the extracellular environment. At the highest AuNPs incubation concentrations (400 nM), cellular uptake in HeLa cells (20 000 cells/well) for the positively charged AuNPs (∼1300 ng/well) was significantly higher than for zwitterionic AuNPs (<100 ng/well). A similar trend was observed in RAW264.7 macrophages (20 000 cells/well), where cellular uptake of positively charged AuNPs was ∼1700 ng/well compared to ∼100 ng/well for the zwitterionic AuNPs. Further prodrug activation studies on HeLa cells indicated that cells coincubated with allyl carbamate-protected doxorubicin (4 μM) and positively charged AuNPs significantly decreased cell viability (∼40%) compared to cells coincubated with pro-doxorubicin and zwitterionic AuNPs (∼65%). These results indicate that intracellular activation of pro-doxorubicin leads to higher therapeutic efficacy than extracellular activation. This innovative approach underlines the power of surface engineering of NPs to control catalytic activity and opens new possibilities for precise targeting in biomedical applications.

**Figure 52:**
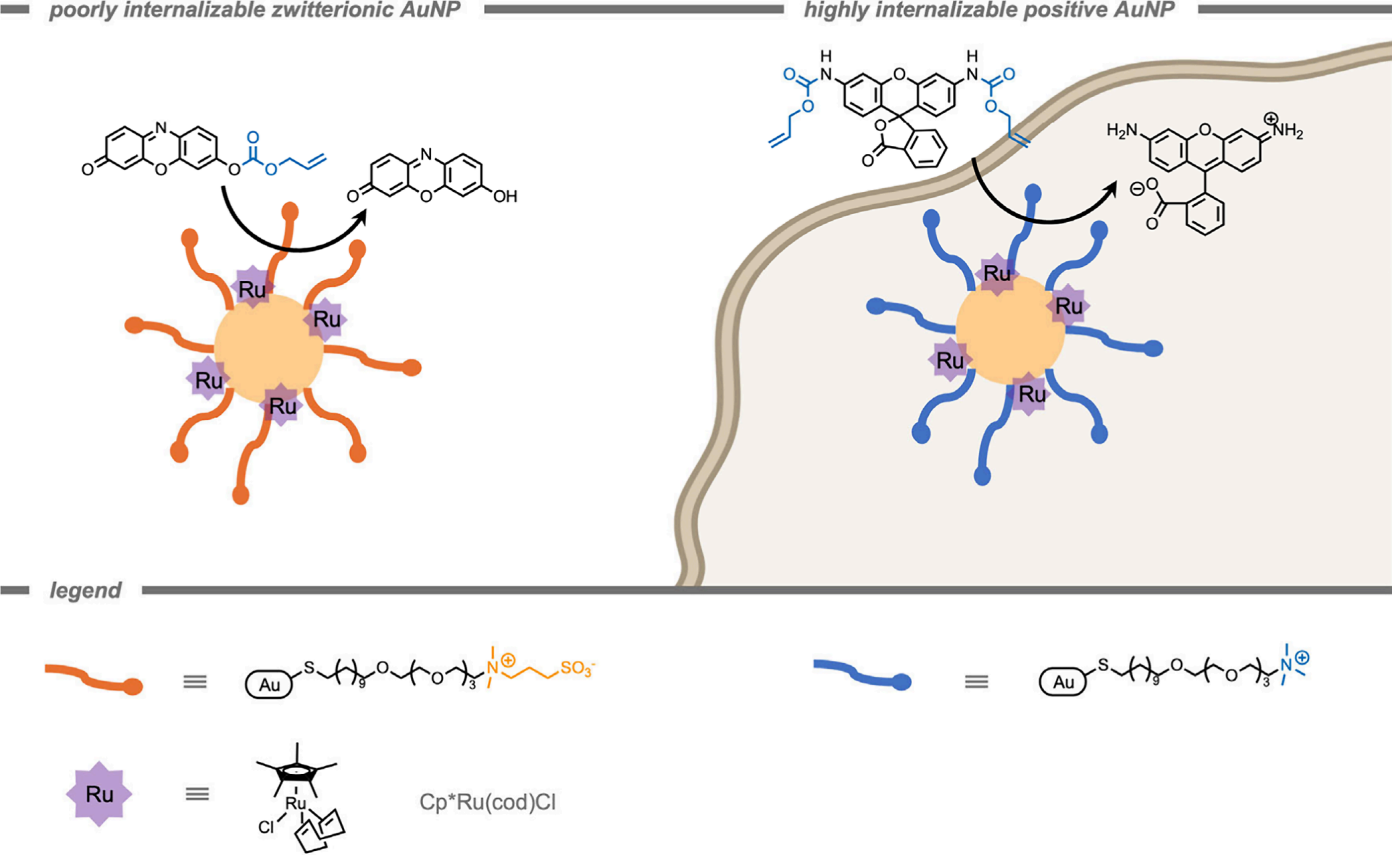
Intra- and extracellular bioorthogonal catalysis mediated by positive and zwitterionic nanozymes, respectively.^285^

While this manuscript was in submission, Rotello and co-workers^286^ reported a light-responsive alginate hydrogel containing Fe­(III) for the activation of propargyl-protected antibiotics.

These examples together show that stimuli-responsive supramolecular catalytic systems allow for enhanced control over reactions in biological media. These stimuli can be external, such as UV light and heating, or internal, such as pH or the ionic strength. Host–guest chemistry was demonstrated as a powerful tool for gatekeeping by blocking access to catalytic sites on a NP surface.

## 8 Summary and Outlook

Supramolecular strategies have significantly advanced the field of bioorthogonal transformations in biological systems. In this review, we summarized the application of supramolecular strategies to this rapidly developing field by focusing on the key advantages. First, supramolecular structures provide scaffolds that shield catalysts or substrates from deactivation by biomolecules. This typically leads to higher biocompatibility of the reaction components with the biological environment. Second, supramolecular architectures have shown promise in achieving more precise targeting of specific cell types and organelles in complex biological environments. This can reduce off-target effects from pharmaceutically active species and allow for increased concentrations at the target location. Third, the use of supramolecular systems can mitigate the use of toxic catalytic species. For example, host–guest chemistry and MOF materials have been developed to perform the CuAAC reaction without high amounts of exogenous toxic copper species. Fourth, the confinement effects inherent in these systems facilitate preorganization, leading to enhanced selectivity and improved reaction kinetics. This may be particularly interesting for reactions at dilute concentrations or reactions with highly reactive intermediates. Finally, the ability to selectively release substrates and control access to catalytic sites has paved the way for stimuli-responsive chemical (catalytic) conversions.

We have found a wide variety of supramolecular systems in the recent literature, all with unique influences on the bioorthogonal chemistry performed in biological systems. Supramolecular MOCs and polymer systems have improved the solubility and stability of catalytic species in biological environments. Larger self-assembled structures based on coordination bonds (e.g., MOFs, and HOFs) are modular in nature, and have allowed for the incorporation of multiple catalysts, substrates, or targeting units, further increasing the complexity as well as the potential applications. The use of vesicles has allowed for improved cellular uptake of catalysts, and vesicles with targeting ability to specific tumor cell types have been reported. Surface-modified catalyst-encapsulating AuNPs have demonstrated stimulus-induced catalytic activity by gatekeeping of the active sites. This wide variety in complex supramolecular systems has allowed for the development of creative strategies to combat intrinsic challenges such as low or off-target (catalyst) activity and limited selectivity in chemical conversions. While cancer treatment is one of the obvious target goals, such supramolecular strategies can potentially find application in a much broader area, including treatment of brain diseases and combat of infections.

Table 1 provides an overview of the chemical transformations discussed per supramolecular strategy throughout this review. It seems clear that a wide variety of chemical transformations have been developed in recent years to complement the key bioorthogonal reactions discussed in Section 2.1. Apart from deprotection reactions (e.g., depropargylations, azide reductions, allyl carbamate deprotections, reduction of Pt, and desilylations), bond-forming transformations such as Heck coupling, Suzuki–Miyaura coupling, and hydroarylation reactions have been explored under biorelevant conditions. Against our expectation, to our knowledge, substrate preorganization or targeting through synthetic supramolecular strategies has not been explored so far for the Staudinger ligation and the SPAAC reaction, despite them being well-documented bioorthogonal reactions.

**Table 1:** Overview of the Utilization of Five Supramolecular Strategies Per Bioorthogonal Reaction Type Mentioned Throughout This Review

reaction	i) stability enhancement	ii) targeting	iii) reducing toxicity	iv) preorganization	v) stimuli-responsive catalysis
Staudinger ligation					
CuAAC	155,167	197,241–243	258,264,265		
Azide–alkyne cycloaddition			119		
SPAAC					
IEDDA		200,214,219,221–223,226,227,229,230		278	
Depropargylation	159,163,174,184,185	204,208,217,244,249,257			279,286
Heck coupling	159				
Suzuki–Miyaura coupling	159				
Azide reduction	164	201,254–256	266		282,283
Allyl carbamate deprotection	152,166,167,175	250			280,281,284,285
Aldehyde reduction	177				
Hydroarylation	178				
Reduction of Pt(IV) to Pt(II)		215	265	275	
Desilylation		245			
Aryl azide photolysis				274	
SPSIC				277	
Thermocyclization				276	

In terms of frequency, targeting has been reported most often as supramolecular strategy throughout this review (27). This reflects the relative ease of incorporation of targeting moieties within supramolecular scaffolds, mainly due to their structural versatility and modularity. The use of supramolecular structures to provide a protective environment around a non-natural catalyst to enhance its stability and biocompatibility has also been reported relatively often (13), demonstrated by a wide variety of structures and scaffolds. Stimulus-responsive catalysis (8) and the mitigation of toxic catalytic species (6) by supramolecular structures have been reported less frequently but are highly promising strategies for the performance of fast and controlled (catalytic) chemical transformations. Although still in the early stages of development, preorganization of substrates and catalysts within a supramolecular scaffold by hydrophobic interactions (5) can allow for the use of highly reactive intermediates in biological environments and improved reaction kinetics.

Despite the impressive progress made so far, the field is just at the beginning of uncovering the opportunities, and as such, the full potential of these supramolecular approaches has yet to be realized. One fundamental challenge lies in the limited opportunities for reaction monitoring in live cells or living organisms, especially for highly complex systems. The characterization and quantification of products and byproducts remain highly challenging, limiting the information on the reaction mechanisms that occur in living systems and the catalytic nature in terms of turnover numbers and turnover frequencies.^70,287^ The influence of endogenous thiols (e.g., H_2_S, and GSH) on the catalytic aryl azide reduction should be further studied to design systems with improved control over this bioorthogonal transformation. Moreover, the dynamic nature of supramolecular systems emphasizes the need for real-time monitoring of the stability of the components and the overall system in physiological environments, but techniques to do so require further development. The interactions of NPs with the biological system are complex and depend on many parameters (e.g., size, surface morphology, surface charge, composition of metals and organic materials, and type of bonding interactions between the NP components). The stability of materials based on dynamic coordination interactions (e.g., MOFs) or hydrogen-bonding interactions (e.g., HOFs) discussed throughout this review is still underexplored in the biological environment. Development of improved monitoring techniques would allow for further understanding of the effect the supramolecular structures have on the activity and stability of the catalyst or the reaction pathway. Finally, as the field is rather interdisciplinary, the terminology used is diffuse and the field would benefit from clear definitions. Throughout recent literature, many supramolecular systems have been described in context of function using ill-defined terms (e.g., nanozyme, nanoreactor, or nanocatalyst). To improve clarity and the accessibility of knowledge in this field, we recommend that the nomenclature should refer to structural characteristics (e.g., surface-coated AuNPs, or an enzyme-encapsulating MOF).

The use of supramolecular systems to perform bioorthogonal reactions in biological systems has developed remarkably over the past decade. As the boundaries of bioorthogonal chemistry are pushed forward, further aided by artificial intelligence-driven design,^288^ supramolecular strategies will undoubtedly play a key role in shaping the future of *in vivo* catalysis and controlled (catalytic) chemical transformations in biological systems. Although the catalytic toolbox is still limited, we foresee that the field of supramolecular bioorthogonal catalysis will open a broader set of (catalytic) chemical conversions, uncovering a plethora of possibilities. As such, we look forward to seeing the development of this creative scientific field.

## Data Availability

Data sharing is not applicable to this article as no new data were created or analyzed in this study.

## Abbreviations

[Fe­(TPP)­Cl]: Iron­(III) tetraphenylporphyrin chloride
5FU: 5-fluorouracil
Ada: adamantane
ADME: absorption, distribution, metabolism, excretion
APTPP: (3-aminopropyl)­triphenylphosphonium bromide
ATP: adenosine triphosphate
AuNP: gold-based nanoparticle
BisTRIS: 2-[Bis­(2-hydroxyethyl)­amino]-2-(hydroxymethyl)­propane-1,3-diol
CaCO_3_: calcium carbonate
CB­[n]: cucurbit­[n]­uril
CD: cyclodextrin
cDNA: complementary DNA
CO: carbon monoxide
cod: cycloocta-1,5-diene
Cp*: pentamethylcyclopentadienyl
CuAAC: Cu^I^-catalyzed [3+2] azide–alkyne cycloaddition
DBCO: dibenzocyclooctyne
DNA: DNA
DNMT1: DNA methyltransferase 1
DPD: dihydropyrimidine dehydrogenase
*E. coli*: *Escherichia coli*
EC_50_: half maximal effective concentration
EC_95_: effective concentration to achieve 95% conversion
EGFP: enhanced green fluorescent protein
EISA: enzyme-instructed self-assembly
EPR: enhanced permeability and retention
ER50: estrogen receptor 50
F^–^: fluoride
FMN: riboflavin-5′-phosphate
GABA: gamma-amino butyric acid
GFP: green fluorescent protein
GSH: glutathione
H22: Hepatoma 22
H_2_O_2_: hydrogen peroxide
H_2_S: hydrogen sulfide
HA: hyaluronic acid
hERG: human ether-a-go-go related gene
HOF: hydrogen-bonded organic framework
IC_50_: half maximal inhibitory concentration
IEDDA: inverse-electron demand Diels–Alder
MAPK: mitogen-activated protein kinase
mDNA: monolayer DNA
MES: 2-(*N*-morpholino)­ethanesulfonic acid
MIL: Matérial Institut Lavoisier
MOC: metal–organic cage
MOF: metal–organic framework
mtDNA: mitochondrial DNA
NADPH: nicotinamide adenine dinucleotide phosphate
NHC: N-heterocyclic carbene ligand
NIR: near-infrared
NP: nanoparticle
^1^O_2_: singlet oxygen
PBS: phosphate-buffered saline
Pd­(dppf)­Cl: [1,1′-bis­(diphenylphosphino)­ferrocene]­dichloropalladium­(II)
PDT: photodynamic therapy
PET: photoinduced electron transfer
PS: photosensitizer
RNA: ribonucleic acid
ROS: reactive oxygen species
SCNP: single chain polymeric nanoparticle
SPAAC: strain-promoted azide–alkyne cycloaddition
SPSIC: strain-promoted sydnonimine cycloalkyne
TACN: 1,4,7-triazacyclononane
TCO: trans-cyclooctene
TLR7: toll-like receptor 7
TMC: transition-metal complex
TOF: turnover frequency
TON: turnover number
TPP: tetraphenylporphyrin
UiO: University of Oslo
UV: ultraviolet
ZIF: zeolitic imidazolate framework
